# Chemical shifts in molecular solids by machine learning

**DOI:** 10.1038/s41467-018-06972-x

**Published:** 2018-10-29

**Authors:** Federico M. Paruzzo, Albert Hofstetter, Félix Musil, Sandip De, Michele Ceriotti, Lyndon Emsley

**Affiliations:** 1 Institut des Sciences et Ingénierie Chimiques, Ecole Polytechnique Fédérale de Lausanne (EPFL), 1015 Lausanne, Switzerland; 2 Institut des Sciences et Génie Matériaux, Ecole Polytechnique Fédérale de Lausanne (EPFL), 1015 Lausanne, Switzerland

## Abstract

Due to their strong dependence on local atonic environments, NMR chemical shifts are among the most powerful tools for strucutre elucidation of powdered solids or amorphous materials. Unfortunately, using them for structure determination depends on the ability to calculate them, which  comes at the cost of high accuracy first-principles calculations. Machine learning has recently emerged as a way to overcome the need for quantum chemical calculations, but for chemical shifts in solids it is hindered by the chemical and combinatorial space spanned by molecular solids, the strong dependency of chemical shifts on their environment, and the lack of an experimental database of shifts. We propose a machine learning method based on local environments to accurately predict chemical shifts of molecular solids and their polymorphs to within DFT accuracy. We also demonstrate that the trained model is able to determine, based on the match between experimentally measured and ML-predicted shifts, the structures of cocaine and the drug 4-[4-(2-adamantylcarbamoyl)-5-tert-butylpyrazol-1-yl]benzoic acid.

## Abstract (web summary)

Solid-state nuclear magnetic resonance combined with quantum chemical shift predictions is limited by high computational cost. Here, the authors use machine learning based on local atomic environments to predict experimental chemical shifts in molecular solids with accuracy similar to density functional theory.

## Introduction

Solid-state nuclear magnetic resonance (NMR) spectroscopy is among the most powerful methods for determining the atomic-level structure and dynamics of powdered and amorphous solids. Notably, solid-state NMR directly probes the local atomic environments and thus allows for characterization without the need for long-range order. This has led to its broad use today in many fields including for instance materials and pharmaceutical chemistry. In the latter the determination of structure and packing is essential to elaborate structure–property relations for formulations in the drug development process.

A revolution in solid-state NMR has occurred with the introduction of accurate methods to calculate chemical shifts^1–3^, in particular using plane wave density functional theory (DFT) methods developed for periodic systems based on the projected augmented wave (PAW)/gauge including PAW (GIPAW) approach^4–6^. This has enabled very rapid development of chemical shift-based NMR crystallography, which is now widely used to validate structures of molecular solids and identify known polymorphs^7–26^, or more recently in combination with crystal structure prediction (CSP) protocols, to determine de novo crystal structures from powders^27–32^. Recent studies also suggest that the structural accuracy of chemical shift-based solid-state NMR crystallography is at least comparable with more traditional methods, such as single crystal X-ray diffraction^33^.

The power of the method arises from the fact that plane wave DFT with the GIPAW method is accurate enough to reproduce the exquisite sensitivity of chemical shifts to changes in local atomic environments. However, this approach also has severe limitations. The cubic scaling of the computational cost with system size prevents the application to larger and more complex crystals, or nonequilibrium structures. If one wanted to use more accurate ab initio calculations, the expense is prohibitive.

Machine learning (ML) is emerging as a new tool in many areas of chemical and physical science, and potentially provides a method to bridge the gap between the need for high accuracy calculations and limited computational power^34–38^. Notably, prediction of chemical shifts for the specific case of proteins in solution using methods based on large experimental databases, using traditional^39–46^ or machine learning approaches^47–49^, have  been considerably successful in predicting shifts based on local sequence and structural motifs, and are widely used today. While there are some examples of machine learned experimental and ab-initio chemical shifts of liquid and gas phase molecules^50–54^, to date there is only one example of machine learning being applied to calculations of chemical shifts in solids, which deals with the specific case of silicas^55^. Molecular solids are characterized by the combinatorial complexity and diversity of organic chemistry, the subtle dependence on conformations, and the long- and short-range effects of crystal packing, which leads to a considerably broader range of chemical environments and possible chemical shieldings than found, e.g., in proteins. All of these aspects, compounded by the fact that there is no extensive database of experimental chemical shifts for molecular solids, make this class of systems particularly challenging for machine learning.

**Fig. 1:**
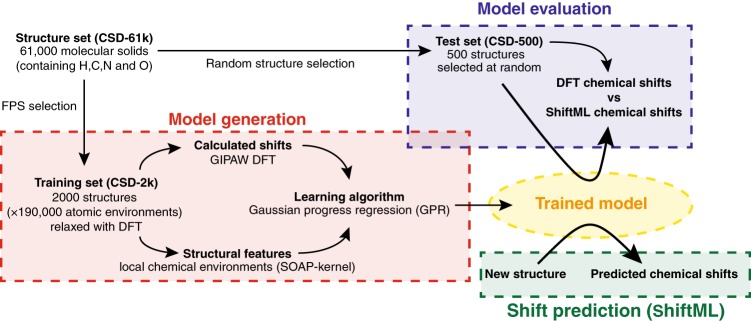
Scheme of the machine learning model used for the chemical shift predictions

Here, we develop a machine learning framework to predict chemical shifts in solids which is based on capturing the local environments of individual atoms, and which makes it well suited for the prediction of such local properties. The protocol is schematically illustrated in Fig. 1. In the absence of a database of experimental shifts, and given that experiments alone do not provide a 1:1 mapping between chemical shifts and a single atomic configuration, we train the model on DFT calculated chemical shifts for structures taken from the Cambridge Structural Database (CSD)^56^ chosen to be as diverse as possible, and then show that the method can predict chemical shifts in a test set with *R*^2^ coefficients between the chemical shifts calculated with DFT and with ML of 0.97 for ^1^H, 0.99 for ^13^C, 0.99 for ^15^N, and 0.99 for ^17^O, corresponding to root-mean-square-errors (RMSEs) of 0.49 ppm for ^1^H, 4.3 ppm for ^13^C, 13.3 ppm for ^15^N, and 17.7 ppm for ^17^O. Predicting the chemical shifts for a polymorph of cocaine, with 86 atoms in the unit cell, using the ML method takes less than a minute of central processing unit (CPU) time, thus reducing the computational time by a factor of between 5 to 10 thousand, without any significant loss in accuracy as compared to DFT.

Most significantly, even though no experimental shifts were used in training, we show that the model has sufficient accuracy to be used in a chemical shift-driven NMR crystallography protocol to correctly determine, based on the match between experimentally measured and ML-predicted shifts, the correct structure of cocaine, and the drug 4-[4-(2-adamantylcarbamoyl)-5-tert-butylpyrazol-1-yl]benzoic acid (AZD8329). We also show that it is possible to calculate the NMR spectra of very large molecular crystals. For this we calculate the chemical shifts of six structures from the CSD with between 768 and 1584 atoms in the unit cells.

## Results

### Training and validation using DFT calculated shifts of known crystal structures

Machine learning models should by definition be trained on the property that is to be predicted. Here, that corresponds to experimental chemical shifts. However, for molecular solids there are currently only around 100 compounds with reliable crystal structures and for which assigned ^1^H or ^13^C shifts have been published, despite the rapidly increasing activity of NMR in crystal structure determination. This is at least an order of magnitude too few structures to hope to determine a reliable prediction model. In this light, we note that today GIPAW chemical shift calculations can accurately reproduce experimental shifts^13,57^. Thus we propose to develop a machine learning model to predict chemical shifts by training the model on a database made up of GIPAW calculated shifts from a large and diverse set of reference crystal structures. If the model can then accurately predict GIPAW chemical shifts, we hypothesize that it should also be in good agreement with experimental shifts. We also note in this context that even if there was a database of experimental shifts, there would be a challenge to machine learning related to the fact that the experiment reports on structures that include dynamics or distributions, making the connection between shifts and environments ambiguous. Learning using GIPAW calculated shifts does not suffer from this problem.

The approach we take to predicting chemical shifts in molecular solids is illustrated in Fig. 1. We use the Gaussian process regression (GPR) framework^58^ to predict the chemical shift of a new atomic configuration based on a statistical model that identifies the correlations between structure and shift for a reference set of training configurations, for which the chemical shifts have been determined by a GIPAW DFT calculation. The predicted chemical shielding for a given atom is given by

1 $${\mathrm{\sigma }}\left( {X} \right) = \mathop {\sum }\limits_i \alpha _ik\left( {{X},{X}_i} \right),$$

where *X* and ${X}_i$ correspond, respectively to a description of the chemical environment of the atom for which we are making a prediction, and that of one of the training configurations. The weights $\alpha _i$ are obtained by requiring that Eq. (1) is consistent with the values computed by DFT for the reference structures. The essential ingredient that differentiates one GPR-based framework from another is the kernel function $k\left( {{X},{X}_i} \right)$, which describes and assesses the similarity between atomic environments, and provides basis functions to approximate the target properties.

Here, our model relies on the smooth overlap of atomic positions (SOAP) kernel^59,60^, in which any atomic environment is represented as a three-dimensional neighborhood density given by a superposition of Gaussians, one centered at each of the atom positions in a spherical neighborhood within a cut-off radius *r*_c_ from the core atom. This framework, combined with GPR, has been used to model the stability and properties of a number of different systems^35,59,60^, and has been extended to the prediction of tensorial properties^61^. We can see that this choice of kernel should be particularly well adapted to predicting chemical shifts, since it describes the local environments around each atom without any simplification, and this is indeed what the chemical shift also probes, as it is determined by the screening of the nucleus from the main magnetic field by the electron density at the nucleus. Note that it should be possible to tune and train other ML methods to accurately predict chemical shifts of molecular crystals. While these possibilities will be explored in future work, the model we present here is already accurate enough to substitute for DFT calculations in chemical shift-based NMR crystallography.

As shown in Fig. 1, in the absence of an experimental database of shifts the model is developed by using a reference training set of structures for which chemical shifts are calculated with GIPAW DFT. To obtain a model which is robust and general, the training set should be as large, as reliable, and as diverse as possible. We first extract from the CSD a large set of about 61,000 structures, corresponding to all the structures in the CSD with fewer than 200 atoms, in order to make DFT chemical shift calculation affordable, and containing C and H and allowing for N and/or O, to reduce the space to organic molecular crystals (we call this set CSD-61k, see Supplementary Methods for details on the structures selection). Given that performing a GIPAW calculation for all of these structures would be prohibitively demanding, we then select a random subset of 500 structures (CSD-500, see Supplementary Note 1 and Supplementary Dataset 2) that are representative of the chemical diversity in the CSD, and we use it to test the accuracy of our model. For cross-validation and training, instead, we select 2000 structures (corresponding to about 185,000 atomic environments) out of the CSD-61k using a farthest point sampling algorithm^62,63^ (CSD-2k, see Supplementary Note 2 and Supplementary Dataset 1). This step ensures near-uniform sampling of the conformational space, improving the quality of the model when using a relatively small number of reference calculations.

To avoid including spurious environments in the model, e.g., environments which might not be well described by DFT, we also automatically detect and discard from the training set atomic environments with values of the DFT calculated shifts that are anomalous based on a cross-validation procedure described in the Supplementary Methods. Note that using this unbiased statistical analysis we detected only a small fraction of environments as outliers (e.g., 211 out of 76,214 for ^1^H, or 0.3%). This is discussed in detail in the Supplementary Methods. We observe that the performance of the model degrades noticeably if one does not use this procedure. This pruning as well as the parameter optimization procedure, described below, were done exclusively using cross-validation on the CSD-2k set. (Notably the test sets were not subject to any curation.)

In order to reduce the computational cost of the training and testing procedures we then finally remove from the training set all the symmetrically equivalent environments. In case of ^1^H, this reduced the size of the training set from 70,000 to about 35,000 different atomic environments. (Details of the selection method and the members of the different sets used are given in the Supplementary Methods and Supplementary Note 3.)

All the atomic positions of the structures in the training and testing sets were relaxed with DFT, using the Quantum Espresso suite^64–66^, prior to calculation of the chemical shieldings using the GIPAW DFT method^4,5^. Note that the DFT relaxation ensures “reasonable” geometries will be used even for crystal structures containing errors (e.g., improbable ^1^H positions). Parameters for the DFT calculations are given in the Supplementary Methods. The calculated chemical shieldings *σ* are converted to the corresponding chemical shifts *δ* through the relationship *δ* = *σ*_ref_ − *σ*. Here, we used a *σ*_ref_ of 30.8 ppm (for ^1^H) and 169.5 ppm (for ^13^C), found through linear regression between the calculated and experimental chemical shifts for cocaine.

**Fig. 2:**
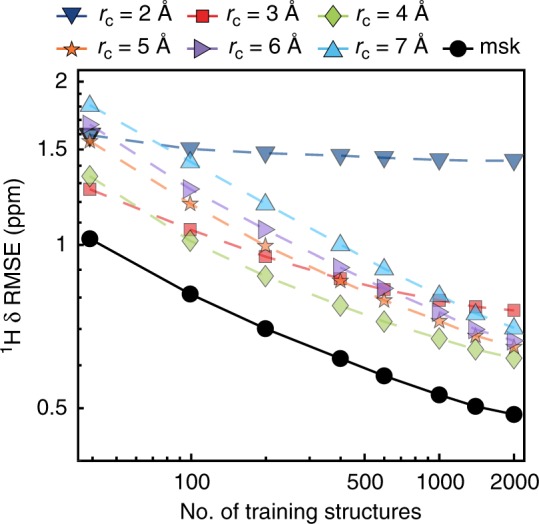
^1^H chemical shift prediction error of the trained model for the CSD-500 set. The RMSE prediction error between chemical shifts calculated with ShiftML and GIPAW DFT is shown for different local environment cut-off radii, and for the multi-kernel (labeled as msk), as a function of the training set size

Figure 2 shows the chemical shift error between the DFT calculations and the ML predictions for the CSD-500 set, which is representative of the expected accuracy for the entire CSD-61k. The figure shows the overall prediction accuracy for ^1^H chemical shifts as RMSE in ppm between the shifts calculated with DFT and with the protocol described above, which we refer to in the following as ShiftML, as a function of the cut-off radius (*r*_c_) and as a function of the number of training structures included from CSD-2k. The effect of the different cut-off radii is clearly visible. For example, for *r*_c_ = 2 Å the prediction error for a small training set (<10 structures or <100 atomic environments) can be smaller than for the larger radii, but does not improve significantly with increasing size of the training set. On the contrary, for *r*_c_ = 7 Å we observe a relatively large prediction error for a small training set, but even with 2000 structures (35,000 environments), the prediction error is still decreasing. A similar behavior is observed for the prediction errors of the ^13^C, ^15^N, and ^17^O chemical shifts (see Supplementary Figures 5–8).

The observed differences in the behavior of the prediction error with respect to *r*_c_ clearly indicates the influence of the different extents of the local environment on the chemical shift. Short-range interactions are sufficient to explain the rough order of magnitude of the shift, but long-range interactions are required to learn about the higher order influences of next-nearest neighbors on shifts. However, for long-range interactions, a much larger number of environments is needed in order to determine the correlation between environment and shift.

We exploit these differences to generate a combined SOAP kernel consisting of a linear combination of the single local environment kernels^35^, with weightings of 256 (*r*_c_ = 2 Å), 128 (*r*_c_ = 3 Å), 32(*r*_c_ = 4 Å), 8 (*r*_c_ = 5 Å and *r*_c_ = 6 Å), and 1 (*r*_c_ = 7 Å). This weighting was determined by rough optimization around values inspired by previous experience^35^, and by cross-validation on the CSD-2k training set (as described in the Supplementary Methods). It is clear that learning with the combined kernel leads consistently to lower prediction errors than any of the single kernels, although the improvement in performance varies between nuclei (see Supplementary Figures 5–8).

**Fig. 3:**
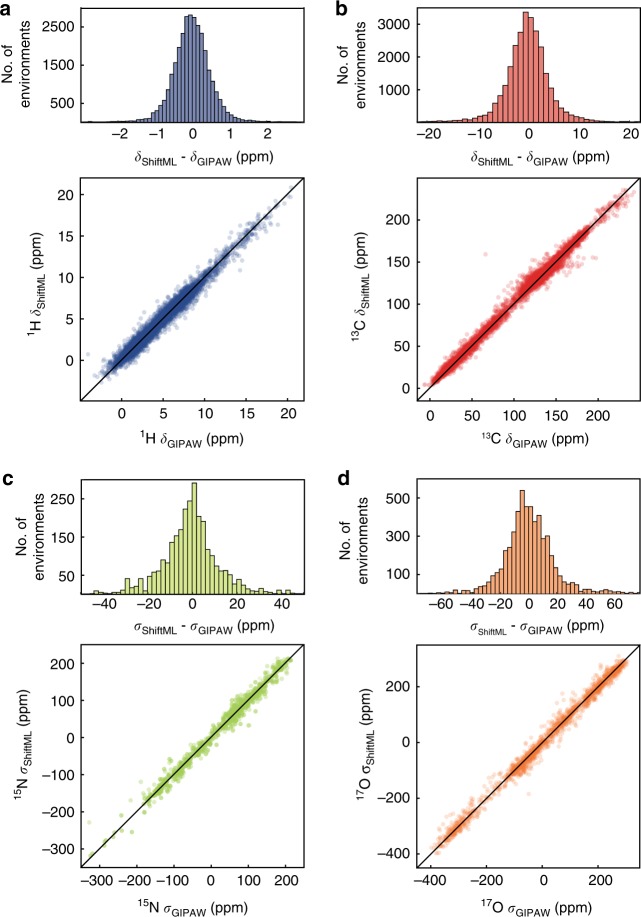
Comparison of predictions from ShiftML and GIPAW DFT. Histograms and scatterplots showing the correlation between ^1^H (**a**), ^13^C (**b**), ^15^N (**c**), and ^17^O (**d**) chemical shifts (shieldings) calculated with GIPAW and ShiftML. The black lines indicate a perfect correlation

Figure 3a–d shows correlation plots between ^1^H, ^13^C, ^15^N, and ^17^O chemical shifts calculated by DFT and by ShiftML for the CSD-500 set trained on the whole CSD-2k combined kernel. Using the combined kernel, we reach an error between ShiftML and DFT calculated chemical shifts of 0.49 ppm for ^1^H (4.3 ppm for ^13^C, 13.3 ppm for ^15^N, and 17.7 ppm for ^17^O). This is very comparable with reported DFT chemical shift accuracy for ^1^H of 0.33–0.43 ppm^13,57^, while requiring a fraction of the computational time and cost: less than 1 CPU minute compared to ~62–150 CPU hours for DFT chemical shift calculation on structures containing 86 atoms (around 350 valence electrons) (see Supplementary Figure 4). For the other nuclei, the ML accuracy is slightly lower than reported values (1.9–2.2 ppm for ^13^C, 5.4 ppm for ^15^N, and 7.2 ppm for ^17^O)^13,57^, which is not surprising as there are (currently) significantly fewer training environments for the heteronuclei than for ^1^H.

The *R*^2^ coefficients between the chemical shifts calculated with DFT and with ShiftML are 0.97 for ^1^H, 0.99 for ^13^C, 0.99 for ^15^N, and 0.99 for ^17^O.

Note that the CSD-500 set used for testing is selected randomly from CSD-61k and not curated. Indeed, we find that many of the atomic environments in the CSD-500 set with a relatively high prediction RMSE possess either unusual cavities inside their crystal structure, possibly indicating an organic cage surrounding noncrystalline solvent or other atoms, or exhibit strongly delocalized π-bonding networks. While there is no theoretical reason preventing the machine learning model from correctly describing such environments, they are rare and not well represented within the training set. CSD-500 thus constitutes a fairly demanding test set.

### Predicting shifts for polymorphs

**Fig. 4:**
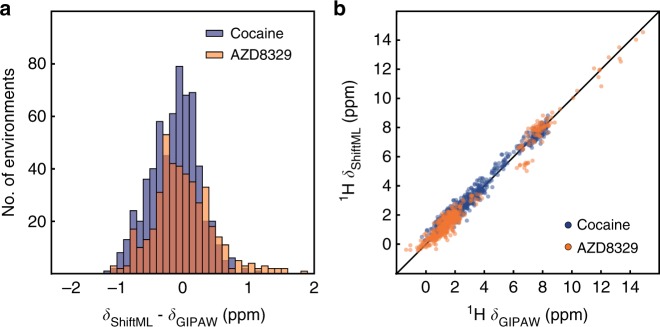
Comparison of predictions from ShiftML and GIPAW DFT for polymorphs of cocaine and AZD8329. **a** Histogram showing the distribution of the differences between ^1^H chemical shifts calculated with GIPAW and with ShiftML for the polymorphs of cocaine (blue), and the polymorphs of AZD8329 (orange). **b** Scatterplot showing the correlation between ^1^H chemical shifts calculated with GIPAW and ShiftML for cocaine (blue) and AZD8329 (orange). The black line indicates a perfect correlation

Having evaluated the power of the trained model to predict the diverse CSD-500 set, we now look at the capacity to predict potentially subtler differences by looking at a set of polymorphs of a given structure. Figure 4 shows the correlation between the ^1^H shifts calculated by GIPAW DFT and by ShiftML for 30 polymorphs of cocaine and 14 polymorphs of AZD8329, all of which were previously generated with a CSP procedure^16,27^. The figure clearly shows that ShiftML is able to accurately predict the differences in ^1^H chemical shift for different polymorphs.

We find a chemical shift prediction error (RMSE) between GIPAW DFT and ShiftML for ^1^H for the cocaine polymorphs of 0.37 ppm and for AZD8329 of 0.46 ppm. Note that these values are slightly less than for the CSD-500 set, which might be expected when looking at these two fairly typical organic structures, and suggesting that the randomly selected CSD-500 indeed provides a good overall benchmark.

Note that for these cases the DFT structure optimization and GIPAW chemical shift calculation were done with a different DFT program (CASTEP)^67^, which suggests that ShiftML is robust with respect to small deviations from the fully optimized structures. (As shown in the Supplementary Figure 2, performing the prediction using Quantum Espresso consistently leads to a comparable prediction accuracy.)

For the heteronuclei we obtain an RMSE between GIPAW DFT and ShiftML for cocaine of 3.8 ppm for ^13^C, 12.1 ppm for ^15^N, and 15.7 ppm for ^17^O. For AZD8329 the ^15^N and ^17^O RMSEs are proportionally larger (17.7 and 54.7 ppm), and we attribute this to the fact that the molecule contains a rather unusual C–O…H–N /C–O…H–O H-bonded dimer structure, for which the learning is thus even sparser than for the heteronuclei in general. To illustrate the unusual nature of this motif, we note that the calculated ^17^O shifts using DFT also change by up to 50 ppm for structures relaxed either by the CASTEP protocol used in ref. ^30^, or the Quantum Espresso protocol used here (the RMSE between ML and DFT for the Quantum Espresso relaxed structures is reduced to 10.9 and 11.5 ppm for ^15^N and ^17^O, respectively). The RMSE of 4.0 ppm for ^13^C for AZD8329 is in line with the other systems.

### Predicting experimental shifts and structure determination

Further, the significance of the method is illustrated by comparison to experimentally measured shifts. This comparison is particularly important since the training protocol did not involve any experimentally measured chemical shifts. We find that the predicted shifts are accurate enough to allow crystal structure determination for both cocaine and AZD8329 from powder samples in a chemical shift-driven NMR crystallography approach.

**Fig. 5:**
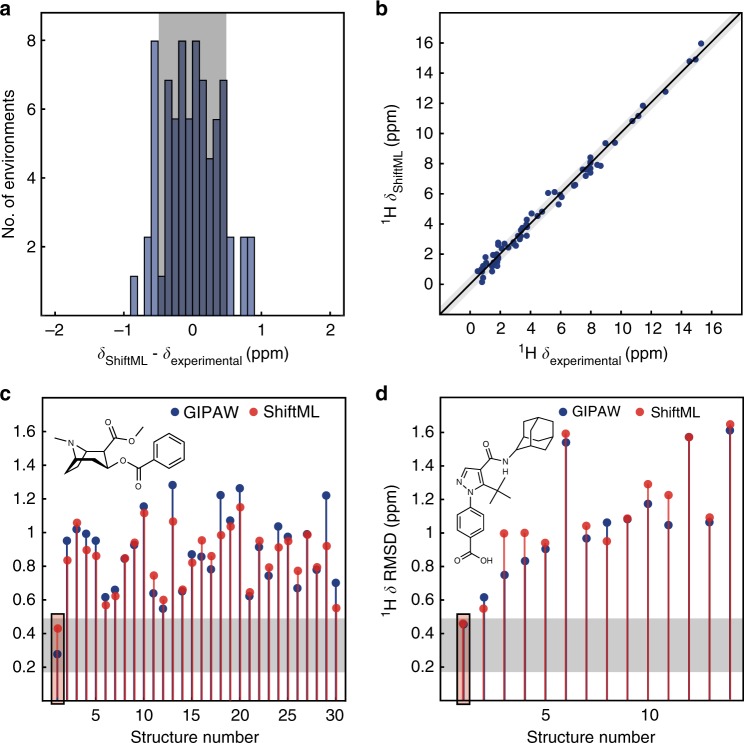
Comparison of ShiftML to experimentally measured shifts. **a** Histogram showing the distribution of differences between experimentally measured ^1^H chemical shifts and ^1^H chemical shifts calculated with ShiftML for six different crystal structures (see Supplementary Methods for the structures and numerical values of the shifts). **b** Scatterplot showing the correlation between these experimentally measured ^1^H chemical shifts and shifts calculated with ShiftML. **c**, **d** Comparison between calculated and experimental ^1^H chemical shifts for the most stable structures obtained with CSP for cocaine (**c**) and AZD8329 (**d**). For each candidate structure an aggregate RMSE is shown between experimentally measured shifts and shifts calculated using either GIPAW (blue) or ShiftML (red). The gray zones represent the confidence intervals of the GIPAW DFT ^1^H chemical shift RMSD, as described in the text^13^, and candidates (in **c** and **d**) that have RMSEs within this range would be determined as correct crystal structures using a chemical shift-driven solid-state NMR crystallography protocol

**Fig. 6:**
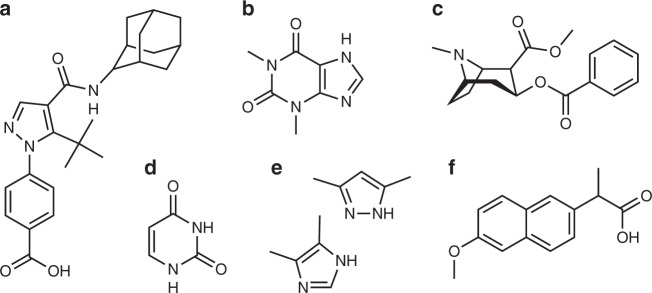
Chemical structures of the six molecules used to evaluate the correlation between experimentally measured ^1^H chemical shifts and the shifts calculated by ShiftML. The structures are given as AZD8329 (**a**), theophylline (**b**), cocaine (**c**), uracil (**d**), 3,5-dimethylimidazole and 4,5-dimethylimidazole (**e**) and naproxen (**f**)

Figure 5a, b shows the correlation between experimentally measured ^1^H chemical shifts and the ^1^H chemical shifts calculated by ShiftML for crystal structures of the six molecules shown in Fig. 6 (numerical values of the experimental chemical shifts, the crystal structures, and the shifts calculated with ShiftML are given in the Supplementary Methods and Supplementary Dataset 8). The comparison between experimental and calculated ^1^H chemical shifts for all crystal structures (for a total of 68 shifts) gives an error (RMSE) of 0.39 ppm and a *R*^2^ coefficient of 0.99. This compares very favorably to the equivalent agreement found between GIPAW DFT and experiment which for this set of structures is a RMSE of 0.38 ppm.

Figure 5c, d shows in blue the RMSE between DFT calculated and experimental ^1^H chemical shifts for the 30 polymorphs predicted by CSP to have the lowest energy for cocaine and the 14 *cis* polymorphs of AZD8329. For both molecules the only structure in agreement with the GIPAW DFT calculations, to below a ^1^H DFT chemical shift confidence interval of 0.49 ppm^13^, is the correct crystal structure. In the same plots we overlay the result where the experimental shifts are now compared to shifts predicted with ShiftML. Note that the RMSE between experiment and the predicted chemical shifts follows the same trends as for the DFT calculated shifts, and that here again the only structures below the confidence interval of 0.49 ppm are the two correct crystal structures. Note, that the cut-off of 0.49 ppm with respect to experiment has been evaluated for GIPAW DFT chemical shifts^13,57^ and to rigorously repeat the CSP procedure for the ML method, the accuracy should be re-evaluated using more extensive benchmarking of ShiftML to experiment, which will be the subject of further work.

### Predicting shifts for large structures

**Fig. 7:**
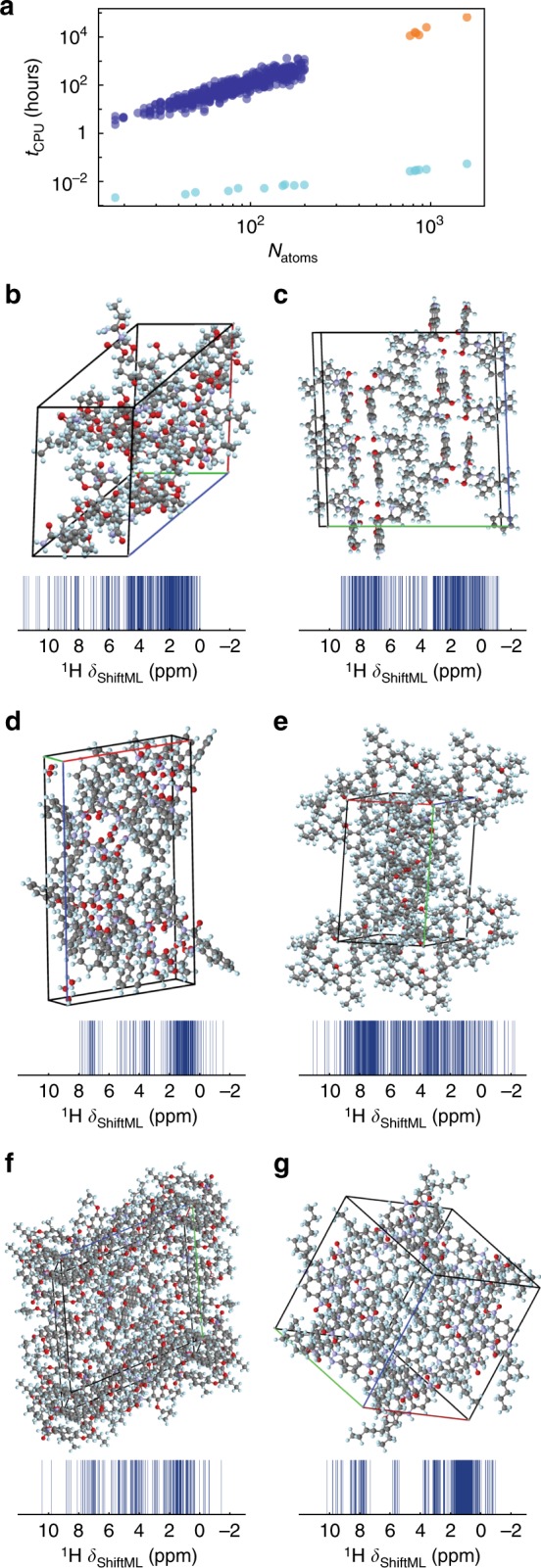
Chemical shift calculation times and large structures. **a** DFT GIPAW calculation time (blue) and ShiftML prediction time (turquoise) for different system sizes. The GIPAW DFT calculation time for the six large structures (orange) is estimated from a cubic dependence on the number of valence electrons in the structure (see Supplementary Methods). **b**-**g** 3D-shemes and ^1^H NMR spectra predicted with ShiftML, of the six large molecular crystals with CSD refcodes: **b** CAJVUH^69^, *N*_atoms_ = 828, **c** RUKTOI^70^, *N*_atoms_ = 768, **d** EMEMUE^71^, *N*_atoms_ = 860, **e** GOKXOV^72^, *N*_atoms_ = 945, **f** HEJBUW^73^, *N*_atoms_ = 816, and **g** RAYFEF^74^, *N*_atoms_ = 1584

Finally, we note that the accuracy of the method does not depend on the size of the structure, and that the prediction time is linear in the number of atoms. For the structures we calculate here the prediction time actually appears nearly constant, because it is dominated by the loading time of the reference SOAP vector (see Fig. 7a). We have used this method to calculate the NMR spectra (shown in Fig. 7b–g) for six structures from the CSD having among the largest numbers of atoms per unit cell (containing only H, C, N, and O), with between 768 and 1584 atoms per unit cell. (See Supplementary Figure 10 for the chemical formula). The values of the predicted chemical shifts are given as CSD-6 in the Supplementary Note 4. Figure 7a shows the comparison between the GIPAW calculation time and the required ML prediction time. We estimate that the whole calculation would require around 16 CPU years by GIPAW. ShiftML requires less than 6 CPU minutes to calculate the shifts for all the compounds.

## Discussion

We have presented a ML model based on local environments to predict chemical shifts of molecular solids containing HCNO to within current DFT accuracy. The *R*^2^ coefficients between the chemical shifts calculated with DFT and with ShiftML are 0.97 for ^1^H, 0.99 for ^13^C, 0.99 for ^15^N, and 0.99 for ^17^O. The approach allows the calculation of chemical shifts for structures with ~100 atoms in less than 1 min, reducing the computational cost of chemical shift predictions in solids by a factor of between five to ten thousand compared to current DFT chemical shift calculations, and thereby relieves a major bottleneck in the use of calculated chemical shifts for structure determination in solids.

Far from being just a benchmark of a machine-learning scheme, the method is accurate enough to be used to determine structures by comparison to experimental shifts in chemical shift-based NMR crystallography approaches to structure determination, as shown here for cocaine and AZD8329. The ML model only scales linearly with the number of atoms and, for the prediction of individual structures, is dominated by a constant I/O overhead. Here it allows the calculation of chemical shifts for a set of six structures with between 768 and 1584 atoms in their unit cells in less than 6 min (an acceleration of a factor 10^6^ for the largest structure).

The accuracy of the method is likely to increase further with the size of the training set, and subsequently with the future evolution of the accuracy of the method used to calculate the reference shifts used in training (here DFT), or by using experimental shifts if a large enough set were available. A web version based on the protocol described here is publicly available at http://shiftml.epfl.ch.

The model used here can easily be extended to organic solids including halides or other nuclei, and to network materials such as oxides, and these will be the subject of further work.

## Methods

### Computational details

For the SOAP kernels^59,60^, each atomic environment is represented as a three-dimensional neighborhood density given by a superposition of Gaussians, one centered at each of the atom positions in a spherical neighborhood within a cut-off radius *r*_c_ from the core atom. The Gaussians have a variance $\varsigma$^2^, and a separate density is built for each atomic species. The kernel is then constructed as the symmetrized overlap between the amplitudes representing *X* and *X′*. This degree of overlap thus measures the similarity between the environments *X* and *X′*.

The SOAP and GPR parameters are given in the Supplementary Methods. SOAP-based structural kernels contain several adjustable hyper-parameters, which are discussed in ref. ^60^. However, we have not systematically explored the full parametric space here, instead we chose reasonable values of the parameters without extensive fine-tuning, based on previous experience^35^ and with some optimization by cross-validation on the CSD-2k training set (see Supplementary Methods for details). We also combine kernels computed for different cutoff radii to capture the contributions to shifts from different length scales^35^, as is described in detail above. The calculations of the local environment, the similarity kernel and the weighted correlations were done using the glosim2 package^68^.

In summary, the Supplementary Information contains details on the structure selection, the crystal structure prediction procedure, the DFT calculations, the GPR method, the SOAP kernels, the FPS algorithm, the detection procedure of unusual environments, the NMR crystallography approach, the DFT calculation time estimates, the prediction parameters optimization, the learning curves and the evaluation curves for ^1^H, ^13^C, ^15^N, and ^17^O. Additionally we also provide the ShifML predicted and GIPAW chemical shieldings for all cocaine and AZD8329 polymorphs as well as the geometries, the assigned experimental chemical shifts and the chemical shifts calculated with GIPAW DFT and ShiftML for the comparison to experimentally measured shifts. The Supplementary Information also contains the chemical formula and predicted chemical shieldings of the CSD-6 set predicted with ShiftML, the Refcodes for CSD-2k and CSD-500 and the relaxed geometries and GIPAW DFT calculated chemical shifts of all investigated crystal structures.

### Code availability

The machine learning code to calculate the SOAP environments, the kernels, and the chemical shifts is called glosim2, and is publicly available at https://github.com/cosmo-epfl/glosim2. The DFT codes used to optimize geometry and calculate chemical shifts are available from the corresponding developers.

## Electronic supplementary material

Supplementary Information

Description of Additional Supplementary Files

Supplementary Data 1

Supplementary Data 2

Supplementary Data 3

Supplementary Data 4

Supplementary Data 5

Supplementary Data 6

Supplementary Data 7

Supplementary Data 8

Supplementary Data 9

## Data Availability

The authors declare that the data supporting the findings of this study are available within the paper and its supplementary information. In particular, all crystallographic structures used are referenced in the Supplementary Information and are publicly available at the Cambridge Structural Database. The relaxed crystal structures with the chemical shieldings calculated by GIPAW DFT and ShiftML are included in the supplementary information files and in the Supplementary Datasets.
