# Supplementary material for: Chemical shifts in molecular solids by machine learning
Source: Nat Commun. 2018 Oct 29;9:4501. doi: 10.1038/s41467-018-06972-x (PMC6206069; doi:10.1038/s41467-018-06972-x)
Supplement: Supplementary file 1 — Supplementary Information [file 41467_2018_6972_MOESM1_ESM.pdf]

Supplementary Information

Chemical Shifts in Molecular Solids by Machine Learning

Paruzzo *et al.*

Supplementary Methods

Crystal Structures.

All the crystal structures of CSD-61k and CSD-500 were obtained from the Cambridge Structural Database (CSD).[1] A total of 88,648 structures was downloaded from the CSD, using two different selection criteria: the maximum number and the type of atoms contained in the unit-cell. We selected only structures with a maximum of 200 atoms, containing either (i) only H and C or (ii) H, C and one heteroatom between N and O or both. From this set we extracted a subset of 61,012 (CSD-61k) structures by removing (i) structures with missing protons, and (ii) structures where the distance of at least one pair of atoms was smaller than the sum of their covalent radii minus 0.3 Å. In addition, structures containing partial occupancy were resolved by keeping only the first of the atoms with partial occupancy. If we were not able to resolve the disorder, the entire structure was not included. The disorder was assumed to be removed, if the number of atoms, for each atom type, was an integer multiple of the number of atoms given in the chemical formula. Note, that as we sorted through more that 60,000 structures, the whole procedure was automatized and we didn't manually select the most stable structure for a given disorder. However, here we are not looking for ground state structures but instead only for physically reasonable structures to expand our data-set. The remaining structures were then used to create both the training (CSD-2k, given as Supplementary Dataset 1) and the testing set (CSD-500, given as Supplementary Dataset 2) for the ^{1}H, ^{13}C, ^{15}N and ^{17}O chemical shift prediction as described in the main text. The test set (CSD-500) was created by randomly picking 500 structures from the CSD-61k excluding the structures already selected for the training set.

Crystal Structure Prediction.

Here we use a set of possible polymorphs predicted by CSP for cocaine and the drug 4-[4-(2-adamantylcarbamoyl)-5-tert -butylpyrazol-1-yl]-benzoic acid (also referred as AZD8329). General details on the CSP protocol can be found in ref. 2. In chemical shift based NMR crystallography, the CSP trial polymorphs are tested against experimental parameters (^{1}H chemical shifts) to determine the experimental crystal structure.

In this work we used 30 possible polymorph structures of cocaine and 14 trial structures of AZD8329 generated with CSP. The 30 structures of cocaine were obtained from the Electronic Supporting Information (ESI) of ref. 3, and correspond to the most stable polymorphs obtained with CSP. Crystal structures of AZD8329 were obtained from the ESI of ref. 4, and correspond to the 14 most stable predicted polymorphs with the *cis* conformation of the amide bond. From the same sources we obtained chemical shifts for each structure calculated with GIPAW[5,6] using the DFT program CASTEP[7] and the experimental chemical shifts. Labels for the different polymorphs of each structure are based on their DFT calculated energy, with 1 being the most stable trial polymorph of a given molecule.

DFT Calculations.

All the DFT calculations were carried out using the DFT program Quantum ESPRESSO.[8,9] For all structures in the CSD-2k and CSD-500 databases we first carried out geometry optimization using plane wave DFT. We used ultrasoft pseudopotentials with GIPAW[5,6] reconstruction, H.pbe-kjpaw_psl.0.1.UPF, C.pbe-n-kjpaw_psl.0.1.UPF, N.pbe-n-kjpaw_psl.0.1.UPF and O.pbe-n-kjpaw_psl.0.1.UPF from the USSP pseudopotential database [http://www.quantum-espresso.org/pseudopotentials].[10] The optimizations were done with the generalized-gradient-approximation (GGA) density functional PBE,[11] using a wave-function energy cut-off of 60 Ry, a charge density energy cut-off of 240 Ry and without k-points. The Grimme van der Waals dispersion correction[12] was included in order to account for van der Waals interactions. The geometry optimization was done relaxing all atomic positions while keeping the lattice parameters fixed.

A single point energy (scf) was then computed for the relaxed geometry, using higher wave-function and charge density energy cut-offs which were set to 100 Ry and 400 Ry respectively. For this calculation we also used a Monkhorst-Pack grid of *k*-points[13] corresponding to a maximum spacing of 0.06 Å^{-1} in the reciprocal space. The *k*-points and energy cut-off values were optimized to ensure convergence of the electron density. Finally, we calculated the chemical shielding $\square_{DFT}$ using the GIPAW method, with the same parameters as used in the scf calculation.

All the relaxed geometries, together with the GIPAW DFT calculated chemical shifts, are given in extended-xyz format.

Note that using a convergence threshold of in the scf calculation of 1e^{-8} Ry leads to a residual random error on the macroscopic contribution to the shifts of the order of 0.1 ppm. Fully converged results can be achieved with a threshold of 1e-^{12}-1e-^{14} Ry.

Machine Learning.

We model the isotropic chemical shielding as a function of the local environment A using a Gaussian Process Regression framework, that assumes that chemical shift values predicted by the model can be written as

$$\sigma(A) = f(A) + \varepsilon, \quad (1)$$

where the function f is a Gaussian Process[14] and ε represents the error of the prediction, which is modeled as independent identically distributed Gaussian variates, with variance σ_n^2. Following the Gaussian Process Regression framework, the isotropic chemical shielding function becomes:

$$\sigma(A) = \sum_{i=1}^{N} \alpha_i k(A, X_i)^{\zeta}, \quad (2)$$

where $\{X_i\}_{i=1}^{N}$ is a training set of *N* reference local environments for which the isotropic chemical shieldings are known, k is a kernel function measuring the covariance between local environments and ζ is a hyperparameter controlling the sensitivity of the kernel.

The weights can be computed by inverting the kernel matrix $K_{ij} = k(X_i, X_j)$ computed between the reference configurations, including a regularization that depends on an estimate of the intrinsic uncertainty in the fit, due to errors in the training set, the limitations of the model or the reduced number of training configurations

$$\alpha_i = \sum_j [K^{\zeta} + \sigma_n^2 1]^{-1}{}_{ij}\, \sigma(X_j). \quad (3)$$

To assess the correlation between local atomic environments *A* and *B*, we use the SOAP kernel[15] defined by the rotationally invariant overlap between smooth representations of their atomic density:

$$k(A,B) = \int_{SO(3)} \left| \int_{\mathrm{R}^3} \rho_{\mathrm{A}}(\vec{\mathbf{r}}) \rho_{\mathrm{B}}(\hat{R}\vec{\mathbf{r}}) \mathrm{d}\vec{\mathbf{r}} \right|^2 d\hat{R}, \quad (4)$$

where the density is built as a superimposition of Gaussians having width ς, centered on the atoms within a cutoff distance of the central atom in the environment

$$\rho_A(\vec{\mathbf{r}}) = \sum_{i \in A} exp\, [\| \vec{\mathbf{r}} - (\vec{\mathbf{r}}_{\mathbf{i}} - \vec{\mathbf{r}}_{\mathbf{A}}) \|^2 / 2\varsigma^2] f_c(|\vec{\mathbf{r}}_i - \vec{\mathbf{r}}_{\mathbf{A}}|). \quad (5)$$

The details of the construction, and the extension to the case with many atomic species, are given in refs. 16 and 17.

Farthest Point Sampling Algorithm.

Given that a GPR model is essentially an interpolation procedure between the reference configurations, it is crucial that training points are chosen to cover as uniformly as possible the space of structures for which one wants to perform predictions. To achieve this uniform sampling we use a farthest point selection algorithm[18,19] to sort the CSD-61k in descending order of "diversity". Essentially, we select a first structure at random, and then pick the others in the sequence such that

$$k = \underset{k \in CSD-61k}{argmax} \; \underset{j \in selection}{min} |X_k - X_j|, \quad (6)$$

where the distance is the kernel-induced distance associated with an average SOAP kernel for the entire structure.[16] The CSD-2k set corresponds to the first 2,000 configurations identified with this procedure.

Detection of Unusual Environments.

The quality of the training set is essential to ensure the optimal performance of a machine learning algorithm. However, the individual curation of the 2,000 molecular crystals of the CSD-2k dataset would be very time consuming and cumbersome. Note, that the 2,000 molecular crystals correspond to around 35,000 symmetrically non-equivalent atomic environments for ^{1}H alone and the following detection procedure is applied directly to the individual atomic environments instead of the whole molecular crystals.

We automate this detection procedure by assessing the 'instability' of the prediction of the shielding of a given local environment using the difference between the predictions of several GPR models and the reference DFT-shielding.

We define this indicator as:

$$\varepsilon(X) = \frac{1}{M} \sum_{i=1}^{M} (y_i(X) - y(X)), \quad (7)$$

where each of the *M* models is made using a 2-fold split of the shuffled training set that does not include the structure *X*. In total we generate *M*=40 models, where each is generated using a different random shuffling of the data. Environments with a large value of $|\varepsilon(X)|$ are not well-described by the rest of the training set within the SOAP-GPR framework. Note, that the error would cancel out in the case of random noise within the prediction, while a large value of $|\varepsilon(X)|$ corresponds to a systematic error in the predicted chemical shielding, that could be associated to the limitations listed below. We define local environments to be unusual when $|\varepsilon(X)|$ is larger than three times the standard deviation of $|\varepsilon(X)|$ over the whole training set, and we then do not use them for training.

We perform this elimination procedure on the CSD-2k dataset using a single kernel for each element (r_c = 4.5 Å for ^{1}H, 4 Å for ^{13}C, 4 Å for ^{15}N and 3 Å for ^{17}O). The hyperparameters of the single kernels used in the elimination procedure were determined using a grid search and 3-fold cross validation on the uncleaned CSD-2k training set. The ^{1}H environments excluded with this approach are shown in Supplementary Figure 1, while further details for ^{1}H and the other nuclei are listed in the Supplementary Note 3.

It is interesting to see that in several cases we can trace the unusual behavior of the environment to subtle errors in the DFT calculations, or to physical phenomena that are ill described within our DFT model (metallic systems, zwitterions,…). However, note that we are not systematically removing such structures and that the training set still contains many structures with the listed features.

Most of the environments detected as "unusual" are part of zwitterionic structures or charged structures (such as VIWYEH, ZACSOO or EKUJIF). Others are metallic structures ($E_{\mathrm{LUMO}} - E_{\mathrm{HOMO}}$ = 0), such as HAZQUV, QUICNA02, DMEBQU01 or AYUKIP, or have a partially empty unit cell (QAHVUQ). An intrinsic limit of this procedure is the fact that it might detect structures with uncommon functional groups as "anomalies" (e.g. TIMCHX, which is an aziridine – a three membered heterocycle with one amine group, or FIGMAJ which has a cubane group), due to the fact that these structures are not well represented by the used training set. However, with increasing training size, we expect these structures to be better represented and they will not be detected as anomalies anymore.

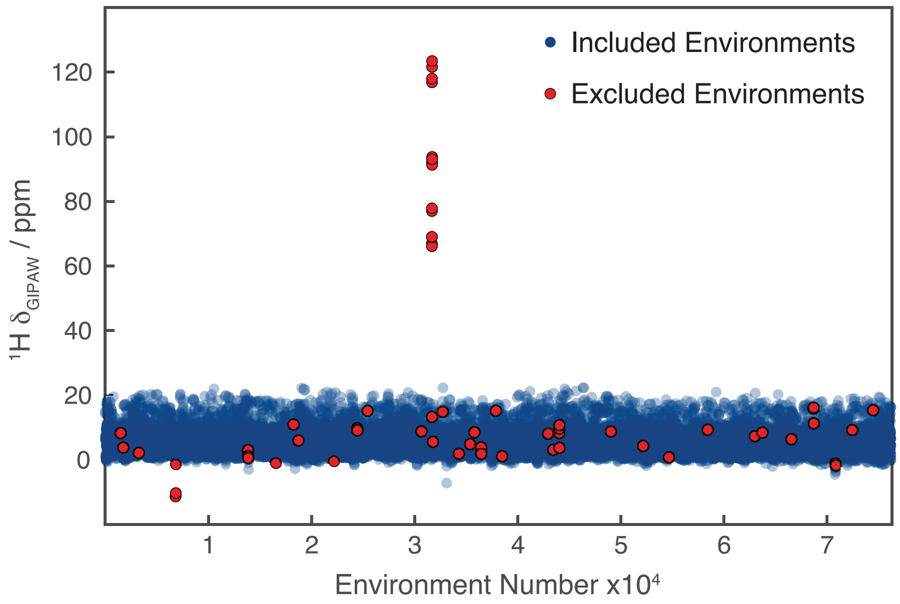

Supplementary Figure 1. ^{1}H chemical shifts of the 76,214 environments in the CSD-2k set. The environments excluded using the unusual structures detection procedure described in the text are shown in red.

NMR Crystallography.

To validate the accuracy of the chemical shifts calculated with ShiftML, we replicated the last step of the protocol for the *ab initio* crystal structure determination of powdered solids[3,4,20] using predicted shifts. This step consists in the comparison between experimental and predicted ^{1}H chemical shifts for the candidate crystal structures selected from a crystal structure prediction method. We perform this analysis for cocaine and form 4 of AZD8329.[3,4] The value □$_{ref}$ for the conversion between chemical shieldings to chemical shifts is calculated for each structure with a linear regression between calculated and experimental shifts, imposing a slope equal to 1. This procedure is done independently for the ^{1}H chemical shieldings calculated with DFT and ShiftML. The geometry of the structures predicted with CSP, as well as their chemical shift values calculated with GIPAW and the experimental chemical shifts of the observed polymorphs were obtained from refs. 3 and 4. Remarkably, the high accuracy shown in Figure 4 was obtained using crystal structures with only ^{1}H positions relaxed and DFT chemical shift calculations carried out using a different program (CASTEP) to the one we used to build our training set (Quantum Espresso). Supplementary Figure 2 shows the results obtained for cocaine and AZD8329 after all-atom optimization and calculation of GIPAW chemical shifts with Quantum Espresso. Here we show fewer structures compared to Figure 4, due to the fact that we limit ourselves to calculate DFT chemical shifts of structures with less than 250 atoms. This selection removes structures 15 for cocaine and structures 2, 11 and 14 for AZD8329. The accuracy is consistent with that reported in Figure 4, although the all-atom optimization leads to some significant structural differences compared to the only ^{1}H relaxed structures, especially for AZD8329. We find a chemical shift prediction error (RMSE) for ^{1}H for cocaine of 0.40 ppm and for AZD8329 of 0.51 ppm, which is very comparable to the expected GIPAW DFT accuracy. For the heteronuclei we obtain, for cocaine and AZD 8329 respectively, 3.5 and 3.4 ppm for ^{13}C, 9.3 ppm and 11.0 ppm for ^{15}N and 12.2 ppm and 11.5 ppm for ^{17}O.

Experimental chemical shifts were referenced to the ^{1}H resonance observed for adamantane at 1.87 ppm with respect to TMS. We used assigned chemical shifts values and we account for rotational dynamics of the methyl groups by averaging the chemical shift values of the three ^{1}H positions to a single value for each methyl group. For AZD8329 the chemical shifts of the CH_2 groups were also averaged. The RMSE calculation was carried out in MATLAB using a home-written script. The chemical structures of cocaine and AZD8329, together with the assignment of the experimental chemical shifts are shown in Supplementary Figure 3 and Supplementary Table 1. ^{1}H chemical shieldings calculated with GIPAW and ShiftML are given as separate .cs files (Supplementary Datasets 3-6), named according to the corresponding structure and method used for DFT calculations. Each file contains the ^{1}H chemical shifts calculated with GIPAW (first column) and predicted with ShiftML (second column) ordered according to Supplementary Table 1. Quantum Espresso relaxed structures are given as xyz files together with the DFT calculated chemical shieldings for all atoms.

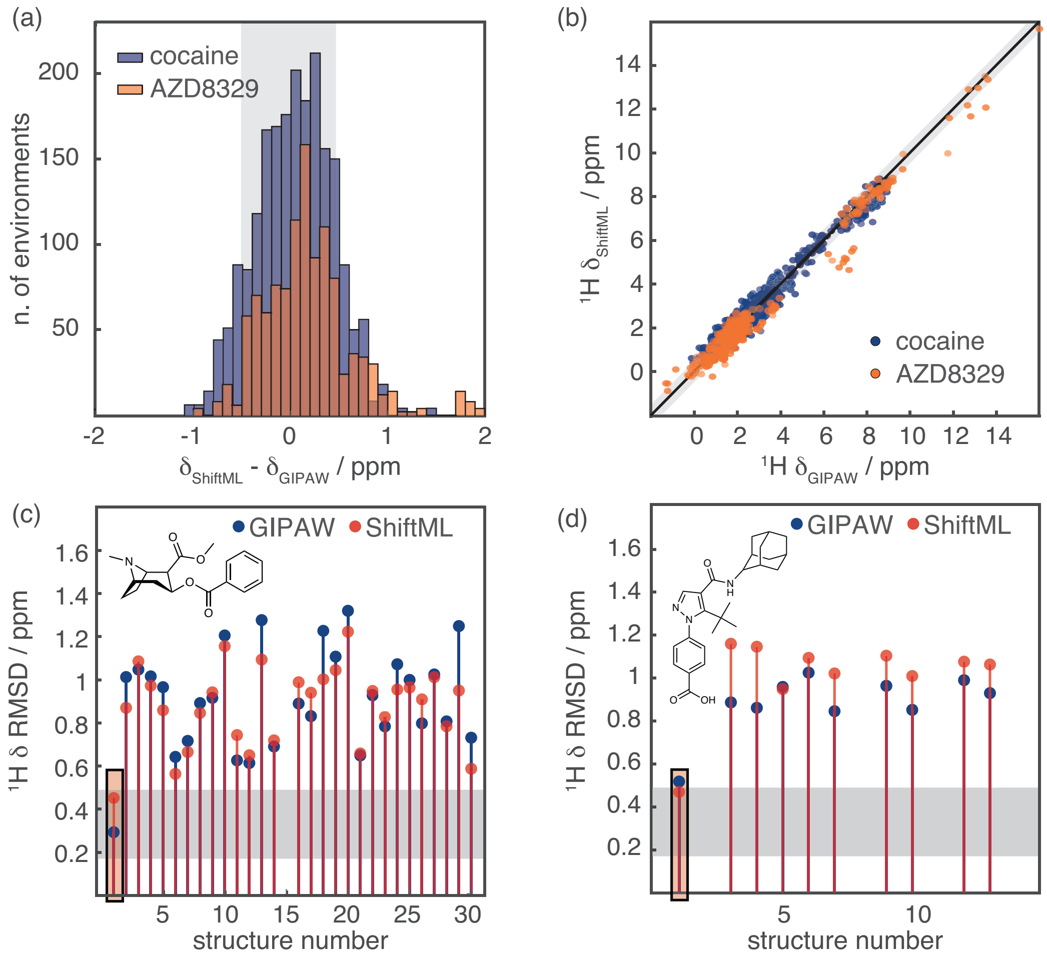

Supplementary Figure 2. NMR crystallography of cocaine and the form 4 of AZD8329. (a) Histogram showing the distribution of the differences between chemical shifts calculated with GIPAW and ShiftML. The blue bars were calculated for the polymorphs of cocaine, and the orange ones for the polymorphs of AZD8329. (b) Scatterplot showing the correlation between GIPAW and ShiftML chemical shifts for cocaine (blue) and AZD8329 (orange). The black line indicates a perfect correlation. (c-d) Comparison between calculated and experimental ^{1}H chemical shifts for the most stable structures obtained with CSP for cocaine (c) and form 4 of AZD8329 (d). Chemical shifts were calculated using GIPAW (blue) and ShiftML (red). The highlighted bars correspond to the candidates that would be selected as correct crystal structures using the chemical shift based solid-state NMR crystallography protocol. In (a-d) the grey zones represent the confidence intervals of the ^{1}H chemical shift RMSD, as described in the text.[20]

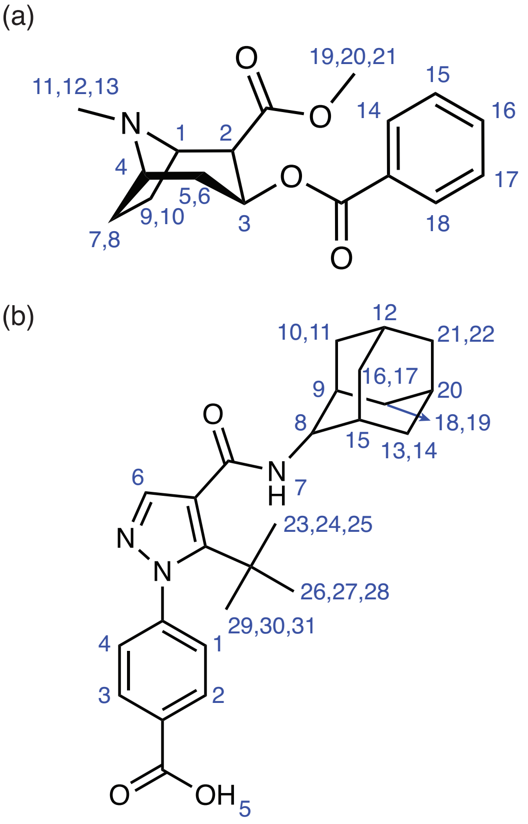

Supplementary Figure 3. Chemical structure of cocaine (a) and AZD8329 (b) and the labelling scheme used here.

Cocaine		AZD8329	
Atom Label	^{1}H δ (ppm)	Atom Label	^{1}H δ (ppm)
1	3.76	1	6.92
2	3.78	2	8.69
3	5.63	3	9.01
4	3.32	4	8.47
5	3.49	5	15.37
6	3.06	6	7.73
7	2.91	7	9.64
8	3.38	8	2.90
9	2.56	9	1.78
10	2.12	10,11	1.88
11,12,13	1.04	12	1.8
14	8.01	13	1.6
15	8.01	14	0.44
15	8.01	15	1.54
17	8.01	16,17	1.88
18	8.01	18,19	0.8
19,20,21	3.78	20	1
		21,22	1.74
		23,24,25, 26,27,28, 29,30,31	0.73

Supplementary Table 1. Experimental chemical shifts of cocaine and the form 4 of AZD8329. The labelling scheme is given in Supplementary Figure 3. When more than one atom corresponds to a single chemical shift value, their values were average

DFT Calculation Times

Supplementary Figure 4 shows the CPU time needed for part of the GIPAW DFT calculations done for this work. The calculations shown in Supplementary Figure 4a were done on polymorph 1 of the cocaine dataset, which contains 86 atoms per unit-cell, while the one in Supplementary Figure 4b were done on 500 structures of the CSD-2k set. In Supplementary Figure 4a the calculation time is plotted as a function of the number of Monkhorst-pack k-points per axis for three different energy-cut-off (E_{cutoff}) values: 40 Ry (blue), 70 Ry (red), 100 Ry (yellow). When increased, these two parameters improve the accuracy of the calculation, but at the same time they drastically increase the computational time needed to carry out the calculation. Supplementary Figure 4b shows the CPU time for the GIPAW chemical shift calculations (blue dots) and for the DFT structure optimizations (green squares) as a function of the number of valence electrons (N_e) per unit-cell. For the GIPAW chemical shift calculations the energy-cut-off was 100 Ry, using a Monkhorst-pack grid with a k-point spacing of 0.06 Å^{-1}. For the DFT structure optimizations the energy-cut-off was 60 Ry and no k-points were used. The red line shows the best fit between the number of valence electrons and the required CPU time for the GIPAW chemical shift calculations as $t_{\text{CPU}} = aN_e^2 + bN_e^3$, where the N_e^3 scaling accounts for the general DFT scaling and the N_e^2 describes the scaling of the matrix inversion, which dominates for small system sizes. The best fit parameters are given as 8.83e-04 (a) and 1.02e-06 (b).

Currently the machine learning model has only been rigorously tested and applied for structures optimized with DFT. Also slight structural changes away from the equilibrium geometry of a molecular crystal have been shown to result in significant changes in the chemical shifts.[21] For this reason, the predictive accuracy of ShiftML for non-equilibrium structures has not yet been quantified. This will be the subject of further work. However, Supplementary Figure 4b clearly shows that the computational cost for the structure optimization is negligible compared to the computational cost of the GIPAW chemical shift calculations.

For structures with $N_e \approx 100$ the GIPAW shift calculations require around 10x more CPU time as the DFT structure optimization, and for $N_e \approx 1{,}000$, 80x more CPU time is required. The run-times of all DFT calculations are given in the Supplementary Dataset 7.

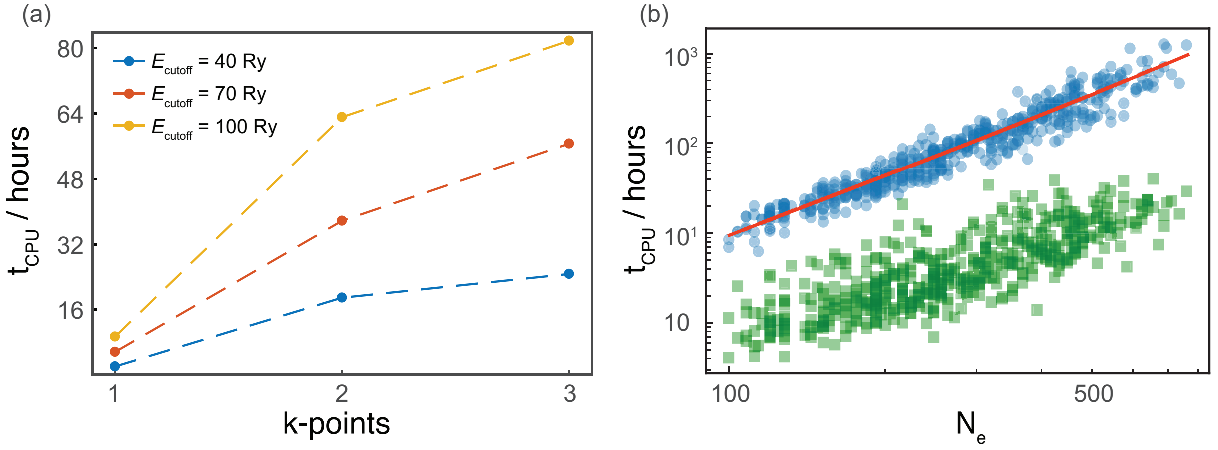

Supplementary Figure 4. CPU time for NMR chemical shift calculations using the GIPAW method. (a) The CPU time is shown as function of the DFT accuracy, determined by the plane-wave cutoff energy $E_{\mathrm{cutoff}}$and the number of k-points in each dimension for polymorph 1 of cocaine. The charge density energy cut-offs were set to $E_\rho = 4E_{\mathrm{cutoff}}$. (b) The CPU time is shown as function of increasing system size in CSD-2k. The green squares and blue dots show individual geometry optimization and GIPAW chemical shift DFT calculations, respectively. The red line shows the best fit between the number of valence electrons and the required CPU time as $t_{\mathrm{CPU}} = aN_e^2 + bN_e^3$ (8), with $a = 0.0162$ and $b = 5.91e - 06$.

ShiftML Prediction Times

The ShiftML run-times shown in Figure 5a are given in the Supplementary Dataset 7.
The ShiftML prediction times scale linearly with the number of atoms per unit cell. However, for all the structures investigated here (from 20 to 1,500 atoms per unit-cell) the required prediction time is dominated by a constant pre-factor associated with the used training set.
Prior to the prediction step, the SOAP reference vector between the test and the training structures is created. This step should be linear in the size of the test-structures, but is currently dominated by the size of the training set. As a result, this takes around one CPU minute for any of the investigated structures here.
The actual subsequent chemical shift prediction, which is linear in the number of atoms within the test-structure, requires at most 10-20 CPU seconds for the large investigated structures.
Note that prior to the chemical shift predictions, the single kernels for all the atomic species must be loaded into virtual memory and the multiscale kernel created. On one CPU this currently takes around 45 minutes. Note, that this has to be done only once, independently of the number and size of the test-structures that are subsequently calculated.

Prediction Parameters, Leaning and Evaluation Curves

Supplementary Table 2 and 3 shows the parameters used for the single and the multi-scale kernel predictions respectively. Using these parameters, we obtained the curves shown in Figure 2 in the main text and the ones shown in Supplementary Figures 5, 6, 7 and 8. Supplementary Figures 5 and 6 show the RMSE and MAE learning curves for ^{1}H, ^{13}C, ^{15}N and ^{17}O for the different local environment cut-off radii, and for the multi-kernel. The training was done on up to 1500 randomly selected frames, while testing on 400 structures selected randomly from the CSD-2k set excluding the structures already selected for the training set. For each point, the random sampling was repeated N times (where N is equal to 300, 255, 215, 170, 130, 85, 45 and 5 respectively for training set sizes of 40, 100, 200, 400, 600, 1000, 1400 and 1500 structures) Supplementary Figure 7 and Supplementary Figure 8 show the results of the predictions of the chemical shifts of the CSD-500 set as a function of the cut-off value and the size of the training set. The parameters for the multi-scale kernel prediction were optimized using 3-fold cross validation on the CSD-2k set. The results for ^{1}H are listed in Supplementary Table 4.

Atom	Cut-off (r_c)	Gaussian width (ς)	l_{max}	n_{max}	σ_n	ζ
^{1}H	2	0.3	9	9	0.1	2
	3	0.3	9	9	0.1	2
	4	0.4	9	9	0.1	2
	5	0.4	9	9	0.1	2
	6	0.5	9	12	0.1	2
	7	0.5	9	12	0.1	2
^{13}C	2	0.3	9	9	0.01	2
	3	0.3	9	9	3.0	2
	4	0.4	9	9	5.0	2
	5	0.4	9	9	3.0	2
	6	0.5	9	12	1.0	2
	7	0.5	9	12	1.0	1
^{15}N	2	0.3	9	9	0.5	2
	3	0.3	9	9	1.0	2
	4	0.4	9	9	0.1	2
	5	0.4	9	9	0.1	2
	6	0.5	9	12	0.1	2
	7	0.5	9	12	0.05	2
^{17}O	2	0.3	9	9	0.5	2
	3	0.3	9	9	5.0	2
	4	0.4	9	9	5.0	2
	5	0.4	9	9	5.0	2
	6	0.5	9	12	1.0	2
	7	0.5	9	12	7.0	2

Supplementary Table 2. Kernel and GPR parameters. The GPR parameters (σ_n and ζ) are the ones used in single kernel predictions.

Atom	Multi-Scale Kernel Weights						σ_n	ζ
	r_c = 2 Å	r_c = 3 Å	r_c = 4 Å	r_c =5 Å	r_c = 6 Å	r_c = 7 Å		
^{1}H	256	128	32	8	8	1	0.1	2
^{13}C	256	512	64	8	8	1	2.0	2
^{15}N	256	128	32	8	8	1	0.1	2
^{17}O	256	128	32	8	8	1	5.0	2

Supplementary Table 3. Kernel weights and GPR parameters used for multi-scale kernel prediction.

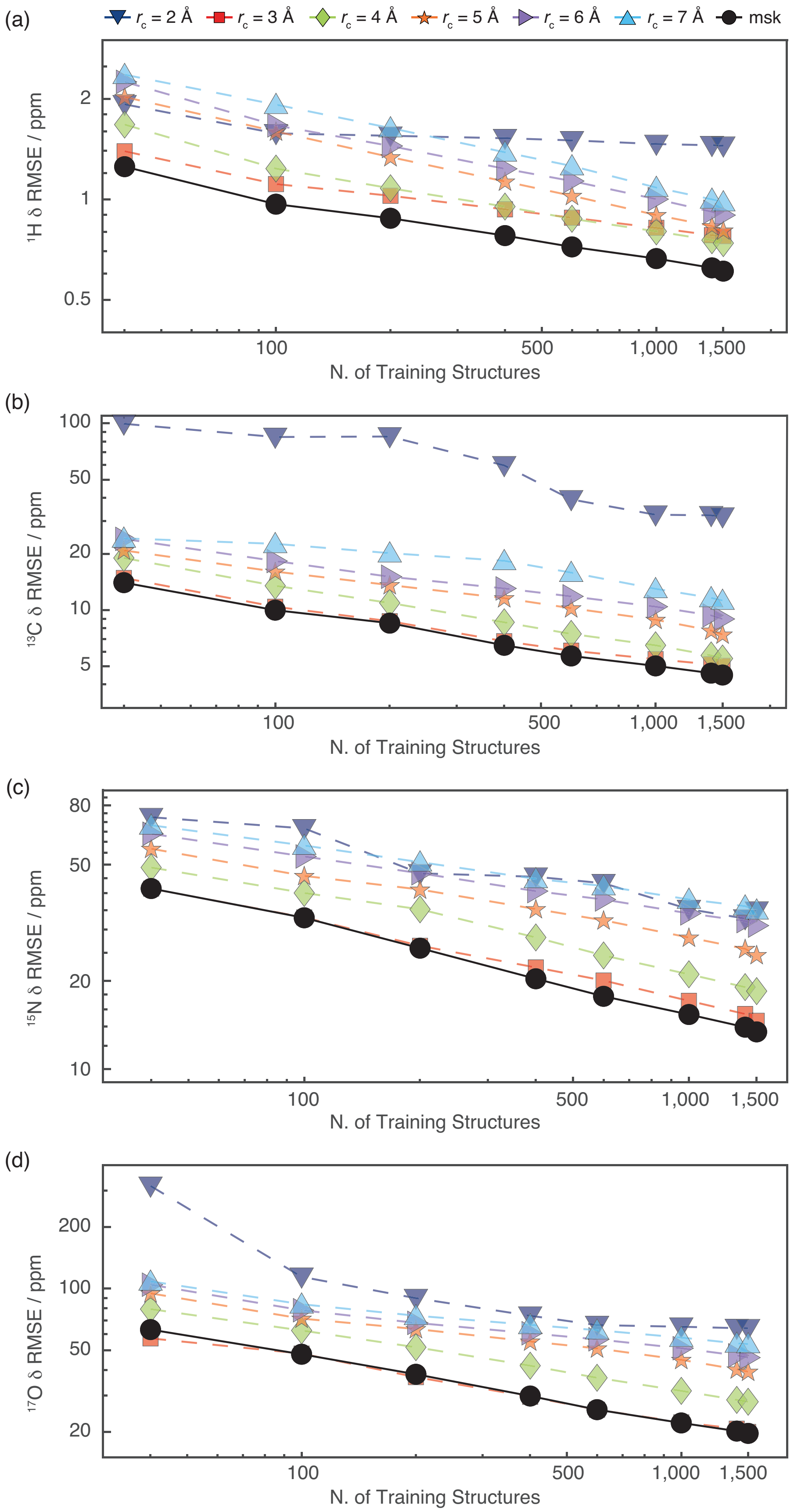

Supplementary Figure 5. RMSE learning curves showing the error between chemical shifts calculated with DFT and ShiftML. The curves are for ^{1}H (a), ^{13}C (b), ^{15}N (c) and ^{17}O (d) chemical shieldings. The multi-kernel learning-curve is labelled as msk.

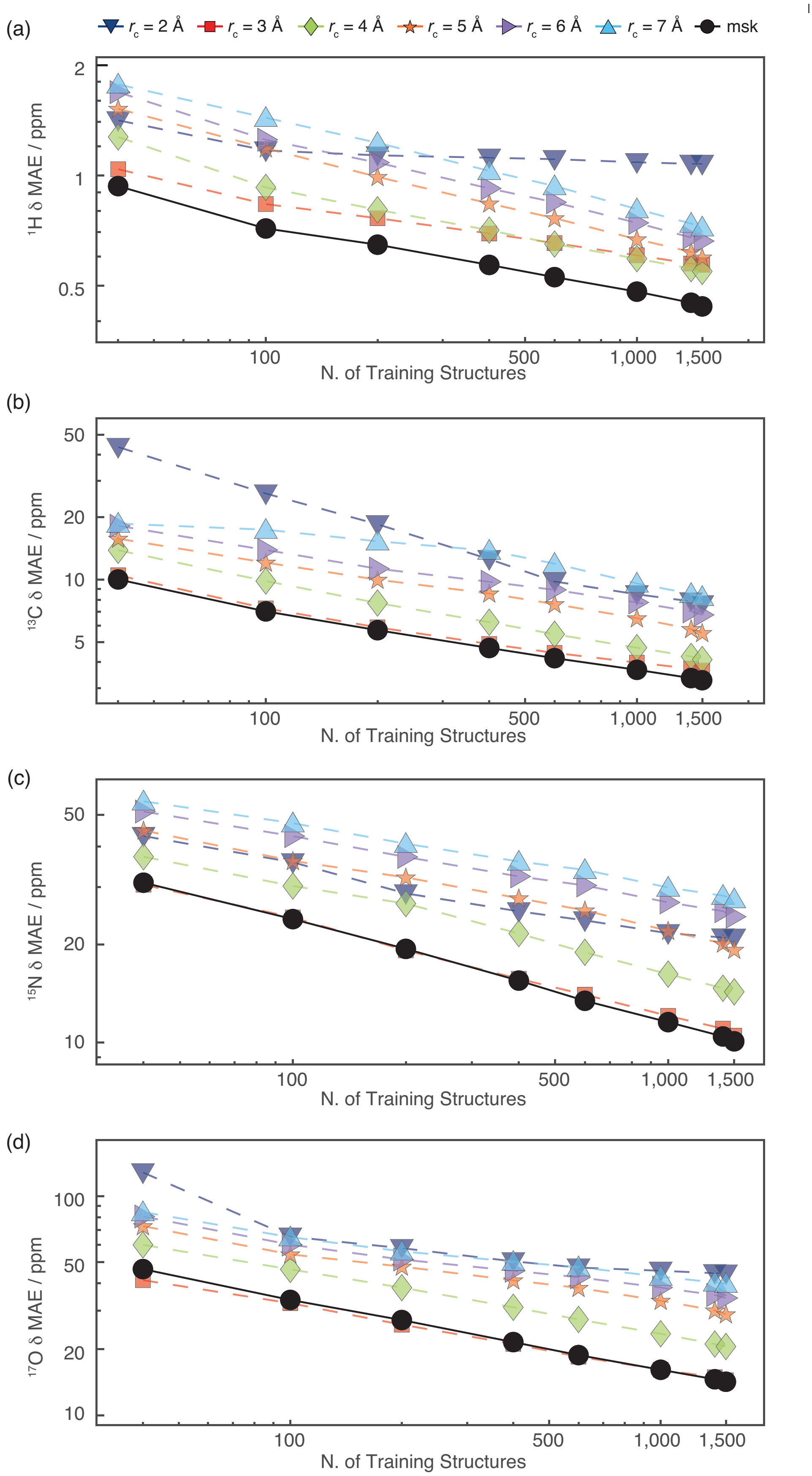

Supplementary Figure 6. MAE learning curves showing the error between chemical shifts calculated with DFT and ShiftML. The curves are relative to ^{1}H (a), ^{13}C (b), ^{15}N (c) and ^{17}O (d) chemical shieldings. The multi-kernel learning-curve is labelled as msk.

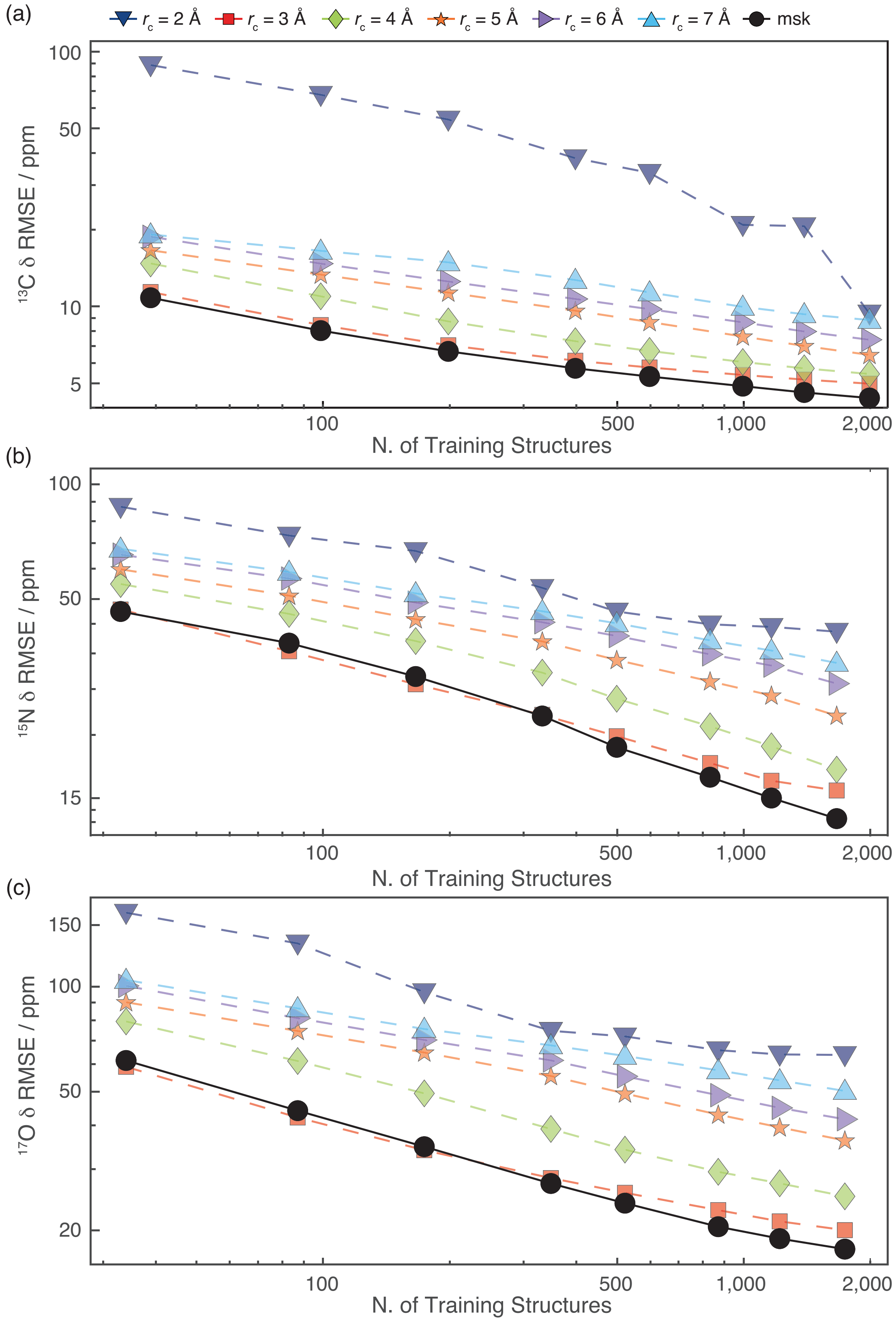

Supplementary Figure 7. RMSE evaluation curves showing the error between chemical shifts calculated with DFT and ShiftML. The curves are relative to ^{13}C (a), ^{15}N (b) and ^{17}O (c) chemical shieldings. The errors were measured for different training set sizes, and evaluated on the CSD-500 test set. The RMSE evaluation curves were acquired as described in the paper. The multi-kernel learning-curve is labelled as msk.

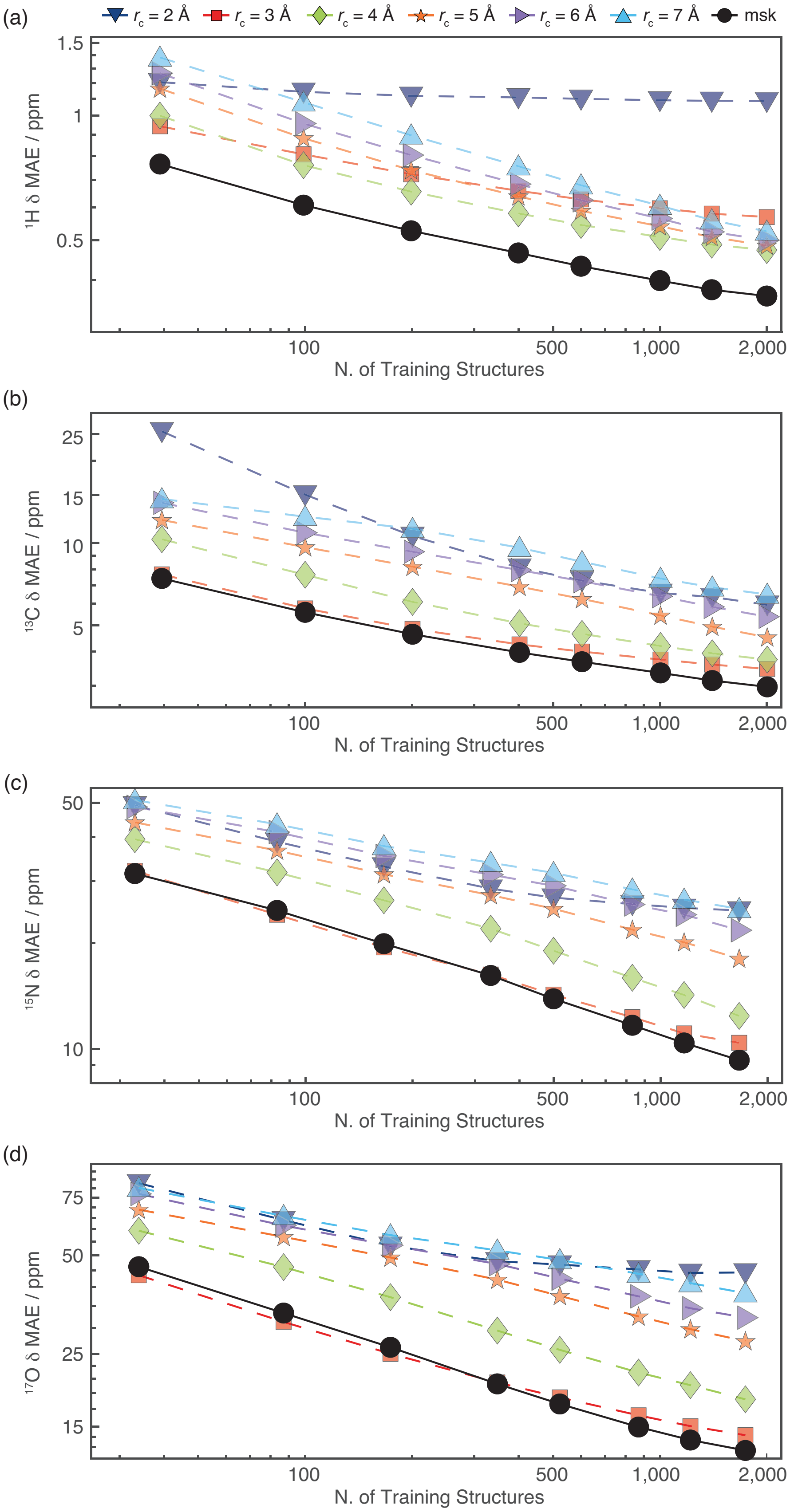

Supplementary Figure 8. MAE evaluation curves showing the error between chemical shifts calculated with DFT and ShiftML. The curves are relative to ^{1}H (a), ^{13}C (b), ^{15}N (c) and ^{17}O (d) chemical shielding. The errors were measured for different training set sizes, and evaluated on the CSD-500 test set. The MAE evaluation curves were acquired as described in the paper. The multi-kernel learning-curve is labelled as msk.

Multi-Scale Kernel Weights						ζ	σ_n	MAE (ppm)	RMSE (ppm)	R^2	SUP (ppm)
r_c =2Å	r_c =3Å	r_c =4Å	r_c =5Å	r_c =6Å	r_c =7Å						
**256**	**128**	**32**	**8**	**8**	**1**	**2**	**0.1**	**0.453**	**0.632**	**0.969**	**6.543**
256	256	32	8	8	1	2	0.1	0.453	0.632	0.969	6.654
256	64	32	8	8	1	2	0.1	0.454	0.634	0.969	6.443
256	128	32	16	8	1	2	0.1	0.455	0.634	0.969	6.477
256	256	32	8	16	1	2	0.1	0.455	0.634	0.969	6.616
256	256	32	16	8	1	2	0.1	0.455	0.634	0.969	6.598
256	128	32	8	16	1	2	0.1	0.455	0.635	0.969	6.505
256	256	64	8	8	1	2	0.1	0.456	0.635	0.969	6.659
256	128	64	8	8	1	2	0.1	0.456	0.636	0.969	6.574
256	256	32	16	16	1	2	0.1	0.457	0.636	0.969	6.559
256	256	64	8	16	1	2	0.1	0.457	0.636	0.969	6.628
256	256	64	16	8	1	2	0.1	0.457	0.637	0.969	6.613
256	128	32	16	16	1	2	0.1	0.457	0.637	0.969	6.438
256	64	32	16	8	1	2	0.1	0.457	0.637	0.969	6.374
256	64	32	8	16	1	2	0.1	0.457	0.638	0.969	6.411
256	128	64	8	16	1	2	0.1	0.458	0.638	0.969	6.542
256	256	64	16	16	1	2	0.1	0.459	0.638	0.969	6.579
256	128	64	16	8	1	2	0.1	0.458	0.638	0.969	6.520
256	64	64	8	8	1	2	0.1	0.459	0.639	0.968	6.507
256	128	64	16	16	1	2	0.1	0.460	0.640	0.968	6.485
256	64	32	16	16	1	2	0.1	0.460	0.640	0.968	6.340
256	64	64	8	16	1	2	0.1	0.461	0.642	0.968	6.479
256	64	64	16	8	1	2	0.1	0.461	0.642	0.968	6.449
256	64	64	16	16	1	2	0.1	0.463	0.644	0.968	6.417
256	128	32	8	8	1	4	0.1	0.467	0.649	0.967	6.337
256	64	32	8	8	1	4	0.1	0.467	0.650	0.967	6.307
256	128	32	8	16	1	4	0.1	0.469	0.652	0.967	6.275
256	128	32	16	8	1	4	0.1	0.469	0.652	0.967	6.245
256	256	32	8	8	1	4	0.1	0.470	0.652	0.967	6.355
256	256	32	8	16	1	4	0.1	0.471	0.653	0.967	6.298
256	128	64	8	8	1	4	0.1	0.471	0.653	0.967	6.215
256	256	32	16	8	1	4	0.1	0.471	0.654	0.967	6.282
256	64	32	16	8	1	4	0.1	0.470	0.654	0.967	6.209
256	128	32	16	16	1	4	0.1	0.470	0.654	0.967	6.186
256	256	64	8	8	1	4	0.1	0.472	0.654	0.967	6.217
256	64	32	8	16	1	4	0.1	0.470	0.654	0.967	6.254
256	256	32	16	16	1	4	0.1	0.472	0.654	0.967	6.226
256	256	64	8	16	1	4	0.1	0.472	0.655	0.967	6.184
256	128	64	8	16	1	4	0.1	0.472	0.655	0.967	6.178
256	256	64	16	8	1	4	0.1	0.473	0.655	0.967	6.166
256	128	64	16	8	1	4	0.1	0.472	0.655	0.967	6.149
256	256	64	16	16	1	4	0.1	0.473	0.656	0.967	6.130
256	64	64	8	8	1	4	0.1	0.472	0.656	0.967	6.217
256	128	64	16	16	1	4	0.1	0.473	0.657	0.967	6.111
256	64	32	16	16	1	4	0.1	0.472	0.657	0.967	6.157
256	64	64	8	16	1	4	0.1	0.474	0.659	0.966	6.183
256	64	64	16	8	1	4	0.1	0.474	0.659	0.966	6.145
256	64	64	16	16	1	4	0.1	0.476	0.661	0.966	6.132
256	256	32	16	8	1	4	1	0.474	0.662	0.965	6.071
256	256	32	16	16	1	4	1	0.474	0.662	0.965	6.080
256	256	64	16	8	1	4	1	0.474	0.662	0.965	6.103

256	256	32	8	8	1	1	0.1	0.473	0.662	0.966	6.622
256	256	32	8	16	1	4	1	0.474	0.662	0.965	6.086
256	256	32	8	8	1	4	1	0.475	0.662	0.965	6.073
256	256	64	16	16	1	4	1	0.474	0.662	0.965	6.115
256	256	64	8	16	1	4	1	0.474	0.662	0.965	6.139
256	256	64	8	8	1	4	1	0.474	0.662	0.965	6.126
256	256	64	8	8	1	1	0.1	0.473	0.663	0.966	6.720
256	256	32	16	8	1	1	0.1	0.475	0.664	0.966	6.573
256	256	64	16	8	1	1	0.1	0.474	0.665	0.966	6.662
256	128	32	8	8	1	1	0.1	0.476	0.665	0.966	6.627
256	256	32	8	16	1	1	0.1	0.476	0.665	0.966	6.628
256	128	64	16	16	1	4	1	0.476	0.666	0.965	6.233
256	256	64	8	16	1	1	0.1	0.475	0.666	0.966	6.724
256	128	64	8	8	1	1	0.1	0.476	0.666	0.966	6.741
256	256	32	16	16	1	1	0.1	0.477	0.667	0.966	6.579
256	128	32	16	8	1	1	0.1	0.478	0.667	0.966	6.565
256	256	64	16	16	1	1	0.1	0.477	0.668	0.966	6.666
256	128	64	16	8	1	1	0.1	0.478	0.668	0.965	6.672
256	64	32	8	8	1	1	0.1	0.479	0.669	0.965	6.597
256	128	32	8	16	1	1	0.1	0.479	0.669	0.965	6.630
256	128	64	8	16	1	1	0.1	0.479	0.669	0.965	6.741
256	64	64	8	8	1	1	0.1	0.479	0.670	0.965	6.740
256	128	32	16	16	1	1	0.1	0.481	0.671	0.965	6.569
256	64	32	16	8	1	1	0.1	0.481	0.671	0.965	6.524
256	128	64	16	16	1	1	0.1	0.481	0.672	0.965	6.671
256	256	64	16	16	1	4	0.01	0.488	0.672	0.965	6.022
256	64	64	16	8	1	1	0.1	0.481	0.672	0.965	6.659
256	256	64	8	16	1	4	0.01	0.489	0.672	0.965	6.098
256	128	64	16	8	1	4	0.01	0.488	0.673	0.965	6.077
256	64	32	8	16	1	1	0.1	0.482	0.673	0.965	6.596
256	128	64	8	8	1	4	0.01	0.488	0.673	0.965	6.174
256	128	64	8	16	1	4	0.01	0.488	0.673	0.965	6.101
256	128	64	16	16	1	4	0.01	0.488	0.673	0.965	6.059
256	256	64	16	8	1	4	0.01	0.489	0.673	0.965	6.089
256	256	64	8	8	1	4	0.01	0.490	0.673	0.965	6.171
256	64	64	8	16	1	1	0.1	0.482	0.674	0.965	6.735
256	128	32	16	8	1	4	0.01	0.489	0.675	0.965	6.226
256	64	32	16	16	1	1	0.1	0.485	0.675	0.965	6.525
256	128	32	16	16	1	4	0.01	0.489	0.675	0.965	6.134
256	128	32	8	8	1	4	0.01	0.490	0.675	0.965	6.362
256	128	32	8	16	1	4	0.01	0.490	0.675	0.965	6.256
256	256	32	16	16	1	4	0.01	0.491	0.675	0.965	6.147
256	64	64	16	16	1	1	0.1	0.484	0.676	0.965	6.656
256	256	64	16	16	1	2	1	0.486	0.676	0.964	6.236
256	256	32	16	8	1	4	0.01	0.491	0.676	0.965	6.243
256	256	32	8	16	1	4	0.01	0.492	0.676	0.965	6.248
256	256	64	16	8	1	2	1	0.486	0.677	0.963	6.223
256	64	64	8	8	1	4	0.01	0.492	0.677	0.965	6.194
256	64	64	16	8	1	4	0.01	0.492	0.677	0.965	6.106
256	256	64	8	16	1	2	1	0.486	0.677	0.963	6.254
256	64	64	8	16	1	4	0.01	0.492	0.677	0.964	6.142
256	256	32	8	8	1	4	0.01	0.493	0.677	0.964	6.356
256	64	64	16	16	1	4	0.01	0.492	0.678	0.964	6.099

256	256	64	8	8	1	2	1	0.487	0.678	0.963	6.243
256	64	32	16	8	1	4	0.01	0.491	0.678	0.964	6.222
256	64	32	8	8	1	4	0.01	0.492	0.678	0.964	6.372
256	256	32	16	16	1	2	1	0.488	0.679	0.963	6.189
256	64	32	16	16	1	4	0.01	0.492	0.679	0.964	6.138
256	256	32	16	8	1	2	1	0.488	0.679	0.963	6.175
256	64	32	8	16	1	4	0.01	0.492	0.680	0.964	6.276
256	256	32	8	16	1	2	1	0.489	0.680	0.963	6.204
256	256	32	8	8	1	2	1	0.490	0.681	0.963	6.191
256	128	64	16	16	1	2	1	0.491	0.684	0.963	6.325
256	128	64	8	8	1	4	0.001	0.500	0.685	0.963	6.257
256	128	64	16	8	1	4	0.001	0.500	0.686	0.963	6.235
256	128	32	8	8	1	4	0.001	0.501	0.687	0.963	6.199
256	128	64	8	16	1	4	0.001	0.501	0.687	0.963	6.258
256	128	32	16	8	1	4	0.001	0.501	0.687	0.963	6.166
256	128	64	16	16	1	4	0.001	0.501	0.687	0.963	6.231
256	64	64	8	8	1	4	0.001	0.502	0.688	0.963	6.301
256	256	64	16	8	1	4	0.001	0.501	0.688	0.963	6.204
256	64	32	8	8	1	4	0.001	0.502	0.688	0.963	6.263
256	256	64	8	8	1	4	0.001	0.501	0.688	0.963	6.231
256	256	64	16	16	1	4	0.001	0.501	0.688	0.963	6.197
256	256	64	8	16	1	4	0.001	0.502	0.688	0.963	6.228
256	64	64	16	8	1	4	0.001	0.503	0.688	0.963	6.279
256	64	32	16	8	1	4	0.001	0.502	0.688	0.963	6.232
256	128	32	16	16	1	4	0.001	0.502	0.688	0.963	6.165
256	128	32	8	16	1	4	0.001	0.502	0.688	0.963	6.204
256	64	64	8	16	1	4	0.001	0.503	0.690	0.963	6.299
256	64	64	16	16	1	4	0.001	0.504	0.690	0.963	6.272
256	256	32	16	8	1	4	0.001	0.503	0.690	0.963	6.134
256	64	32	8	16	1	4	0.001	0.504	0.691	0.963	6.274
256	256	32	16	16	1	4	0.001	0.504	0.691	0.963	6.121
256	64	32	16	16	1	4	0.001	0.504	0.691	0.963	6.234
256	256	32	8	8	1	4	0.001	0.504	0.691	0.963	6.173
256	256	32	8	16	1	4	0.001	0.504	0.692	0.963	6.163
256	256	64	16	16	1	2	0.01	0.507	0.696	0.963	6.987
256	128	64	16	16	1	2	0.01	0.508	0.696	0.963	6.836
256	128	64	16	8	1	2	0.01	0.508	0.696	0.963	6.935
256	256	64	16	8	1	2	0.01	0.508	0.697	0.963	7.095
256	128	64	8	16	1	2	0.01	0.509	0.697	0.963	6.937
256	128	64	8	8	1	2	0.01	0.509	0.697	0.963	7.040
256	256	64	8	16	1	2	0.01	0.509	0.697	0.963	7.084
256	256	64	8	8	1	2	0.01	0.509	0.698	0.963	7.201
256	128	64	16	8	1	2	0.001	0.514	0.700	0.962	6.196
256	256	64	16	16	1	2	0.001	0.514	0.700	0.962	6.138
256	128	64	16	16	1	2	0.001	0.514	0.700	0.962	6.206
256	128	32	16	8	1	2	0.01	0.510	0.701	0.962	7.008
256	64	64	16	8	1	2	0.01	0.512	0.701	0.962	6.838
256	256	64	16	8	1	2	0.001	0.514	0.701	0.962	6.133
256	128	64	8	8	1	2	0.001	0.515	0.701	0.962	6.224
256	128	32	16	16	1	2	0.01	0.510	0.701	0.962	6.885
256	64	64	16	16	1	2	0.01	0.512	0.701	0.962	6.752
256	64	64	8	8	1	2	0.01	0.512	0.701	0.962	6.942
256	128	64	8	16	1	2	0.001	0.515	0.701	0.962	6.240

256	256	64	8	16	1	2	0.001	0.515	0.702	0.962	6.184
256	128	32	8	8	1	2	0.01	0.511	0.702	0.962	7.146
256	64	64	8	16	1	2	0.01	0.513	0.702	0.962	6.857
256	256	32	16	16	1	2	0.01	0.511	0.702	0.962	7.059
256	256	64	8	8	1	2	0.001	0.516	0.702	0.962	6.236
256	128	32	8	16	1	2	0.01	0.512	0.702	0.962	7.015
256	256	32	16	8	1	2	0.01	0.511	0.703	0.962	7.190
256	256	32	8	16	1	2	0.01	0.512	0.704	0.962	7.182
256	64	64	16	8	1	2	0.001	0.518	0.704	0.962	6.267
256	64	32	16	8	1	2	0.01	0.512	0.704	0.962	6.868
256	256	32	8	8	1	2	0.01	0.513	0.704	0.962	7.326
256	64	64	16	16	1	2	0.001	0.518	0.705	0.962	6.273
256	64	64	8	8	1	2	0.001	0.519	0.705	0.962	6.288
256	128	32	16	8	1	2	0.001	0.517	0.705	0.962	6.071
256	64	32	8	8	1	2	0.01	0.513	0.705	0.962	7.003
256	64	32	16	16	1	2	0.01	0.514	0.705	0.962	6.758
256	128	32	16	16	1	2	0.001	0.517	0.706	0.962	6.089
256	64	64	8	16	1	2	0.001	0.519	0.706	0.962	6.302
256	256	32	16	16	1	2	0.001	0.518	0.706	0.962	6.001
256	64	32	8	16	1	2	0.01	0.515	0.707	0.962	6.891
256	128	32	8	8	1	2	0.001	0.518	0.707	0.962	6.208
256	256	32	16	8	1	2	0.001	0.518	0.707	0.961	6.121
256	128	32	8	16	1	2	0.001	0.519	0.707	0.961	6.128
256	64	32	16	8	1	2	0.001	0.519	0.708	0.961	6.174
256	256	32	8	16	1	2	0.001	0.519	0.709	0.961	6.139
256	256	32	8	8	1	2	0.001	0.520	0.709	0.961	6.277
256	64	32	16	16	1	2	0.001	0.520	0.710	0.961	6.190
256	64	32	8	8	1	2	0.001	0.521	0.710	0.961	6.201
256	64	32	8	16	1	2	0.001	0.522	0.712	0.961	6.227
256	256	64	16	16	1	1	1	0.533	0.735	0.956	6.378
256	256	64	16	8	1	1	1	0.534	0.735	0.956	6.384
256	256	64	8	16	1	1	1	0.534	0.736	0.956	6.378
256	256	64	8	8	1	1	1	0.535	0.737	0.956	6.387
256	256	32	16	16	1	1	1	0.535	0.737	0.956	6.400
256	256	32	16	8	1	1	1	0.536	0.737	0.956	6.406
256	256	32	8	16	1	1	1	0.536	0.738	0.956	6.399
256	256	32	8	8	1	1	1	0.537	0.739	0.956	6.408
256	128	64	16	16	1	1	1	0.539	0.743	0.955	6.417
256	256	32	8	8	1	1	0.01	0.568	0.815	0.950	9.881
256	256	64	8	8	1	1	0.01	0.569	0.817	0.950	9.663
256	128	32	8	8	1	1	0.01	0.575	0.819	0.949	9.558
256	64	32	8	8	1	1	0.01	0.578	0.820	0.949	9.758
256	128	64	8	8	1	1	0.01	0.575	0.820	0.949	9.476
256	64	64	8	8	1	1	0.01	0.578	0.820	0.949	9.820
256	256	32	16	8	1	1	0.01	0.574	0.823	0.949	10.020
256	256	32	8	16	1	1	0.01	0.576	0.823	0.949	9.491
256	256	64	16	8	1	1	0.01	0.575	0.824	0.949	9.742
256	256	64	8	16	1	1	0.01	0.577	0.825	0.949	9.262
256	64	32	16	8	1	1	0.01	0.583	0.826	0.949	10.003
256	128	32	16	8	1	1	0.01	0.580	0.826	0.949	9.717
256	64	64	16	8	1	1	0.01	0.584	0.827	0.949	10.064
256	128	32	8	16	1	1	0.01	0.582	0.827	0.949	9.295
256	128	64	16	8	1	1	0.01	0.581	0.828	0.948	9.723

256	64	32	8	16	1	1	0.01	0.586	0.828	0.949	9.444
256	64	64	8	16	1	1	0.01	0.586	0.828	0.948	9.528
256	128	64	8	16	1	1	0.01	0.583	0.828	0.948	9.365
256	256	32	16	16	1	1	0.01	0.582	0.831	0.948	9.621
256	256	64	16	16	1	1	0.01	0.583	0.833	0.948	9.369
256	64	32	16	16	1	1	0.01	0.591	0.834	0.948	9.537
256	128	32	16	16	1	1	0.01	0.588	0.835	0.948	9.296
256	64	64	16	16	1	1	0.01	0.592	0.836	0.948	9.622
256	128	64	16	16	1	1	0.01	0.589	0.836	0.947	9.321
256	64	32	8	8	1	1	0.001	0.945	1.428	0.861	25.290
256	64	64	8	8	1	1	0.001	0.944	1.431	0.861	25.759
256	128	32	8	8	1	1	0.001	0.938	1.440	0.859	30.330
256	128	64	8	8	1	1	0.001	0.938	1.440	0.859	30.942
256	256	32	8	8	1	1	0.001	0.918	1.446	0.857	35.045
256	256	64	8	8	1	1	0.001	0.920	1.446	0.857	35.536
256	64	32	16	8	1	1	0.001	0.955	1.449	0.858	27.466
256	64	64	16	8	1	1	0.001	0.954	1.452	0.857	27.667
256	64	32	8	16	1	1	0.001	0.967	1.460	0.856	25.967
256	128	32	16	8	1	1	0.001	0.948	1.462	0.855	32.147
256	128	64	16	8	1	1	0.001	0.948	1.464	0.855	32.425
256	64	64	8	16	1	1	0.001	0.966	1.464	0.855	25.817
256	256	32	16	8	1	1	0.001	0.928	1.467	0.853	36.276
256	256	64	16	8	1	1	0.001	0.930	1.470	0.853	36.776
256	128	32	8	16	1	1	0.001	0.960	1.475	0.853	31.209
256	128	64	8	16	1	1	0.001	0.960	1.476	0.853	31.211
256	64	32	16	16	1	1	0.001	0.976	1.481	0.852	28.267
256	256	32	8	16	1	1	0.001	0.940	1.483	0.850	36.135
256	256	64	8	16	1	1	0.001	0.942	1.484	0.851	36.396
256	64	64	16	16	1	1	0.001	0.976	1.486	0.852	28.019
256	128	32	16	16	1	1	0.001	0.970	1.497	0.849	33.101
256	128	64	16	16	1	1	0.001	0.970	1.499	0.849	32.781
256	256	32	16	16	1	1	0.001	0.951	1.505	0.847	37.561
256	256	64	16	16	1	1	0.001	0.953	1.508	0.846	37.667

Supplementary Table 4. Optimization of kernel weights and GPR parameters (σ_n and ζ) for multi-scale kernel prediction of ^{1}H chemical shifts. The optimization was carried out on the CSD-2k set, using 3-fold cross validation. For each configuration are reported the corresponding mean absolute error (MAE), root-mean-square error (RMSE), the R-squared (R^2) coefficient and the supremum (SUP). In bold is shown the set of parameters that we selected.

Multi-Scale Kernel Weights						ζ	σ_n	MAE (ppm)	RMSE (ppm)	R^2	SUP (ppm)
r_c =2Å	r_c =3Å	r_c =4Å	r_c =5Å	r_c =6Å	r_c =7Å						
**256**	**512**	**64**	**8**	**8**	**1**	**2**	**2**	**3.38**	**4.63**	**0.99**	**32.85**
256	256	32	8	8	1	2	2	3.39	4.63	0.99	32.33
256	512	32	8	8	1	2	2	3.38	4.64	0.99	31.37
256	256	64	8	8	1	2	2	3.40	4.64	0.99	33.79
256	512	64	16	8	1	2	2	3.39	4.64	0.99	33.10
256	512	32	16	8	1	2	2	3.39	4.64	0.99	31.76
256	512	128	8	8	1	2	2	3.40	4.65	0.99	34.51
256	256	32	16	8	1	2	2	3.41	4.65	0.99	32.68
256	512	128	16	8	1	2	2	3.41	4.65	0.99	34.63
256	256	64	16	8	1	2	2	3.42	4.65	0.99	33.93
256	512	64	32	8	1	2	2	3.41	4.66	0.99	33.37
256	128	32	8	8	1	2	2	3.42	4.66	0.99	33.56

256	512	64	8	16	1	2	2	3.41	4.66	0.99	33.22
256	512	32	32	8	1	2	2	3.41	4.66	0.99	32.20
256	512	32	8	16	1	2	2	3.40	4.66	0.99	32.00
256	512	64	16	16	1	2	2	3.41	4.67	0.99	33.43
128	512	64	8	8	1	2	2	3.41	4.67	0.99	33.70
128	512	32	8	8	1	2	2	3.40	4.67	0.99	32.38
256	512	128	32	8	1	2	2	3.42	4.67	0.99	34.72
256	512	128	8	16	1	2	2	3.42	4.67	0.99	34.75
256	128	64	8	8	1	2	2	3.44	4.67	0.99	35.02
256	512	32	16	16	1	2	2	3.41	4.67	0.99	32.36
256	256	32	8	16	1	2	2	3.42	4.67	0.99	33.13
256	256	64	8	16	1	2	2	3.43	4.67	0.99	34.22
128	512	64	16	8	1	2	2	3.41	4.67	0.99	33.88
256	512	128	16	16	1	2	2	3.43	4.67	0.99	34.85
128	512	32	16	8	1	2	2	3.41	4.68	0.99	32.70
256	128	32	16	8	1	2	2	3.44	4.68	0.99	33.83
256	512	64	32	16	1	2	2	3.43	4.68	0.99	33.65
128	512	128	8	8	1	2	2	3.43	4.68	0.99	35.03
256	512	128	8	8	1	2	4	3.43	4.68	0.99	35.24
256	256	64	16	16	1	2	2	3.44	4.68	0.99	34.41
256	256	32	16	16	1	2	2	3.44	4.68	0.99	33.43
256	512	128	16	8	1	2	4	3.43	4.68	0.99	35.27
256	512	64	8	8	1	2	4	3.42	4.69	0.99	34.89
128	512	128	16	8	1	2	2	3.44	4.69	0.99	35.10
256	512	64	16	8	1	2	4	3.43	4.69	0.99	34.98
256	512	128	32	16	1	2	2	3.44	4.69	0.99	34.92
256	512	32	32	16	1	2	2	3.43	4.69	0.99	32.78
256	128	64	16	8	1	2	2	3.45	4.69	0.99	35.13
128	512	64	8	16	1	2	2	3.43	4.69	0.99	34.00
128	512	32	8	16	1	2	2	3.43	4.69	0.99	32.79
256	512	128	32	8	1	2	4	3.44	4.70	0.99	35.35
256	512	64	8	32	1	2	2	3.44	4.70	0.99	33.71
256	256	64	8	8	1	2	4	3.44	4.70	0.99	36.44
256	512	64	32	8	1	2	4	3.44	4.70	0.99	35.07
128	512	64	16	16	1	2	2	3.44	4.70	0.99	34.14
256	512	128	8	16	1	2	4	3.44	4.70	0.99	35.64
256	512	32	8	8	1	2	4	3.43	4.70	0.99	34.63
256	512	128	8	32	1	2	2	3.45	4.70	0.99	35.05
128	512	32	16	16	1	2	2	3.44	4.70	0.99	33.05
256	512	128	16	16	1	2	4	3.45	4.70	0.99	35.66
256	512	64	16	32	1	2	2	3.45	4.70	0.99	33.91
256	512	32	16	8	1	2	4	3.44	4.70	0.99	34.76
128	512	128	8	16	1	2	2	3.45	4.70	0.99	35.20
256	128	32	8	16	1	2	2	3.46	4.70	0.99	34.35
256	512	64	8	16	1	2	4	3.44	4.70	0.99	35.36
256	512	32	8	32	1	2	2	3.44	4.70	0.99	32.94
256	256	64	16	8	1	2	4	3.45	4.71	0.99	36.59
256	512	64	16	16	1	2	4	3.45	4.71	0.99	35.43
256	512	128	16	32	1	2	2	3.46	4.71	0.99	35.13
256	256	32	8	8	1	2	4	3.45	4.71	0.99	36.08

128	512	128	16	16	1	2	2	3.46	4.71	0.99	35.25
256	128	64	8	16	1	2	2	3.47	4.71	0.99	35.62
256	512	32	16	32	1	2	2	3.45	4.71	0.99	33.22
256	512	128	32	16	1	2	4	3.46	4.71	0.99	35.68
256	512	32	32	8	1	2	4	3.45	4.71	0.99	34.92
256	256	32	16	8	1	2	4	3.46	4.72	0.99	36.18
256	512	64	32	16	1	2	4	3.46	4.72	0.99	35.51
256	512	64	32	32	1	2	2	3.46	4.72	0.99	34.16
256	512	128	32	32	1	2	2	3.47	4.72	0.99	35.17
256	512	32	8	16	1	2	4	3.45	4.72	0.99	35.15
256	64	64	8	8	1	2	2	3.48	4.72	0.99	36.57
256	512	32	16	16	1	2	4	3.46	4.72	0.99	35.26
256	128	32	16	16	1	2	2	3.48	4.72	0.99	34.58
256	256	64	8	16	1	2	4	3.47	4.72	0.99	36.91
256	512	32	32	32	1	2	2	3.47	4.73	0.99	33.57
256	128	64	16	16	1	2	2	3.49	4.73	0.99	35.72
256	256	32	8	8	1	2	1	3.46	4.73	0.99	30.90
256	512	128	8	32	1	2	4	3.47	4.73	0.99	36.10
256	256	64	16	16	1	2	4	3.47	4.73	0.99	37.00
256	512	128	16	32	1	2	4	3.47	4.73	0.99	36.12
256	512	32	32	16	1	2	4	3.47	4.73	0.99	35.40
256	512	64	8	32	1	2	4	3.47	4.74	0.99	35.93
256	512	32	8	8	1	2	1	3.44	4.74	0.99	31.34
256	256	32	8	16	1	2	4	3.47	4.74	0.99	36.63
256	512	64	16	32	1	2	4	3.47	4.74	0.99	35.99
256	128	32	8	8	1	2	1	3.49	4.74	0.99	31.58
256	512	128	32	32	1	2	4	3.48	4.74	0.99	36.13
256	128	64	8	8	1	2	4	3.49	4.74	0.99	38.88
256	256	32	16	16	1	2	4	3.48	4.74	0.99	36.72
256	512	64	8	8	1	2	1	3.46	4.74	0.99	32.33
256	512	32	16	8	1	2	1	3.46	4.74	0.99	31.08
256	256	32	16	8	1	2	1	3.48	4.75	0.99	31.49
256	256	64	8	8	1	2	1	3.48	4.75	0.99	33.24
256	64	64	16	8	1	2	2	3.51	4.75	0.99	36.55
256	512	64	32	32	1	2	4	3.48	4.75	0.99	36.05
256	512	64	16	8	1	2	1	3.47	4.75	0.99	32.76
256	128	32	8	8	1	2	4	3.49	4.75	0.99	38.27
256	512	32	8	32	1	2	4	3.48	4.75	0.99	35.80
256	512	32	16	32	1	2	4	3.49	4.76	0.99	35.89
256	128	64	16	8	1	2	4	3.50	4.76	0.99	39.04
256	256	64	16	8	1	2	1	3.50	4.76	0.99	33.59
256	256	32	8	8	1	4	4	3.44	4.76	0.99	30.91
256	128	32	16	8	1	2	4	3.50	4.76	0.99	38.53
256	512	32	32	32	1	2	4	3.50	4.76	0.99	36.00
256	512	32	8	16	1	2	1	3.47	4.76	0.99	31.19
256	512	32	32	8	1	2	1	3.49	4.77	0.99	31.68
256	256	32	8	16	1	2	1	3.50	4.77	0.99	31.64
256	128	64	8	8	1	2	1	3.52	4.77	0.99	34.03
256	128	32	16	8	1	2	1	3.52	4.77	0.99	32.02
256	512	64	32	8	1	2	1	3.49	4.77	0.99	33.33

256	512	64	8	16	1	2	1	3.48	4.77	0.99	32.81
256	512	128	8	8	1	2	1	3.49	4.77	0.99	34.68
256	64	64	8	16	1	2	2	3.52	4.77	0.99	37.07
256	512	32	16	16	1	2	1	3.49	4.77	0.99	31.41
256	512	128	16	8	1	2	1	3.50	4.77	0.99	34.91
256	512	64	16	16	1	2	1	3.50	4.77	0.99	33.20
256	128	64	8	16	1	2	4	3.51	4.78	0.99	39.30
256	128	32	8	8	1	4	4	3.46	4.78	0.99	31.96
256	256	64	8	16	1	2	1	3.51	4.78	0.99	33.73
256	256	32	16	8	1	4	4	3.45	4.78	0.99	31.24
256	256	64	8	8	1	4	4	3.46	4.78	0.99	31.59
256	256	32	16	16	1	2	1	3.52	4.78	0.99	32.14
256	128	32	8	16	1	2	4	3.52	4.79	0.99	38.79
256	128	64	16	8	1	2	1	3.54	4.79	0.99	34.24
256	512	32	8	8	1	4	4	3.44	4.79	0.99	30.81
256	512	128	32	8	1	2	1	3.52	4.79	0.99	35.16
256	128	64	16	16	1	2	4	3.53	4.79	0.99	39.45
256	64	32	16	16	1	2	2	3.54	4.79	0.99	35.86
256	256	64	16	16	1	2	1	3.53	4.79	0.99	34.03
256	128	32	8	16	1	2	1	3.53	4.79	0.99	32.22
256	512	64	32	16	1	2	1	3.52	4.79	0.99	33.68
256	512	128	8	16	1	2	1	3.51	4.79	0.99	34.93
256	512	32	32	16	1	2	1	3.51	4.79	0.99	32.14
256	512	64	8	8	1	4	4	3.45	4.79	0.99	31.20
256	512	128	16	16	1	2	1	3.52	4.79	0.99	35.14
256	64	64	16	16	1	2	2	3.55	4.79	0.99	37.06
256	512	32	16	8	1	4	4	3.45	4.79	0.99	30.99
256	256	64	16	8	1	4	4	3.47	4.79	0.99	31.90
256	512	64	16	8	1	4	4	3.46	4.80	0.99	31.51
256	128	32	16	16	1	2	4	3.53	4.80	0.99	39.02
256	256	32	8	16	1	4	4	3.47	4.80	0.99	31.19
256	128	32	16	8	1	4	4	3.48	4.80	0.99	32.00
256	512	128	32	16	1	2	1	3.54	4.80	0.99	35.36
256	512	64	8	32	1	2	1	3.52	4.81	0.99	33.48
256	512	32	8	32	1	2	1	3.52	4.81	0.99	31.79
256	128	64	8	8	1	4	4	3.49	4.81	0.99	32.40
256	128	64	8	16	1	2	1	3.55	4.81	0.99	34.44
256	64	64	8	8	1	2	1	3.56	4.81	0.99	34.75
256	256	32	8	8	1	4	2	3.47	4.81	0.99	31.89
256	512	64	16	32	1	2	1	3.53	4.81	0.99	33.80
256	512	32	8	16	1	4	4	3.46	4.81	0.99	30.77
256	128	32	16	16	1	2	1	3.56	4.81	0.99	32.65
256	256	32	16	16	1	4	4	3.48	4.81	0.99	31.47
256	256	64	8	16	1	4	4	3.49	4.81	0.99	31.76
256	512	32	16	32	1	2	1	3.53	4.81	0.99	32.24
256	512	64	8	16	1	4	4	3.47	4.81	0.99	31.36
256	512	32	32	8	1	4	4	3.47	4.81	0.99	31.51
256	512	64	32	8	1	4	4	3.47	4.82	0.99	31.90
256	64	64	8	8	1	2	4	3.55	4.82	0.99	41.56
256	128	32	8	8	1	4	2	3.50	4.82	0.99	31.11

256	512	32	16	16	1	4	4	3.47	4.82	0.99	31.15
256	512	64	16	16	1	4	4	3.48	4.82	0.99	31.64
256	512	128	8	8	1	4	4	3.48	4.82	0.99	31.91
256	512	128	8	32	1	2	1	3.54	4.82	0.99	35.26
256	512	128	16	32	1	2	1	3.55	4.82	0.99	35.45
256	256	32	16	8	1	4	2	3.49	4.82	0.99	31.74
256	512	128	16	8	1	4	4	3.49	4.82	0.99	32.12
256	128	64	16	16	1	2	1	3.57	4.82	0.99	34.63
256	512	64	32	32	1	2	1	3.55	4.82	0.99	34.18
256	128	32	8	16	1	4	4	3.50	4.83	0.99	32.45
256	256	64	16	16	1	4	4	3.50	4.83	0.99	32.03
256	128	64	16	8	1	4	4	3.51	4.83	0.99	32.55
256	512	32	32	32	1	2	1	3.55	4.83	0.99	32.84
256	256	64	8	8	1	4	2	3.50	4.83	0.99	31.64
256	512	128	32	32	1	2	1	3.56	4.83	0.99	35.63
256	64	64	16	8	1	2	4	3.56	4.83	0.99	41.61
256	64	64	16	8	1	2	1	3.59	4.84	0.99	34.80
256	512	32	32	16	1	4	4	3.49	4.84	0.99	31.62
256	512	64	32	16	1	4	4	3.49	4.84	0.99	32.00
256	512	128	8	16	1	4	4	3.50	4.84	0.99	32.03
256	128	32	16	8	1	4	2	3.52	4.84	0.99	31.55
256	512	32	8	8	1	4	2	3.47	4.84	0.99	33.25
256	512	128	32	8	1	4	4	3.50	4.84	0.99	32.39
256	256	64	16	8	1	4	2	3.51	4.84	0.99	32.06
256	256	32	8	16	1	4	2	3.50	4.84	0.99	31.45
256	512	128	16	16	1	4	4	3.50	4.84	0.99	32.22
256	512	32	16	8	1	4	2	3.48	4.85	0.99	33.30
256	512	64	8	8	1	4	2	3.49	4.85	0.99	32.34
256	128	32	16	16	1	4	4	3.52	4.85	0.99	32.47
256	512	64	16	8	1	4	2	3.50	4.85	0.99	32.44
256	128	64	8	16	1	4	4	3.53	4.85	0.99	32.66
256	512	32	8	32	1	4	4	3.50	4.85	0.99	31.08
256	512	64	8	32	1	4	4	3.50	4.85	0.99	31.58
256	128	64	8	8	1	4	2	3.53	4.85	0.99	32.24
256	64	64	8	16	1	2	4	3.58	4.86	0.99	41.93
256	256	32	16	16	1	4	2	3.52	4.86	0.99	31.55
256	512	64	16	32	1	4	4	3.51	4.86	0.99	31.83
256	512	32	16	32	1	4	4	3.50	4.86	0.99	31.40
256	64	64	8	16	1	2	1	3.60	4.86	0.99	35.13
256	256	64	8	16	1	4	2	3.53	4.86	0.99	31.89
256	512	128	32	16	1	4	4	3.52	4.86	0.99	32.47
256	512	32	8	16	1	4	2	3.50	4.86	0.99	32.92
256	128	32	8	16	1	4	2	3.54	4.86	0.99	31.47
256	512	32	32	8	1	4	2	3.51	4.86	0.99	33.04
256	512	64	32	8	1	4	2	3.51	4.87	0.99	32.35
256	64	32	16	16	1	2	1	3.61	4.87	0.99	33.48
256	512	64	8	16	1	4	2	3.51	4.87	0.99	32.08
256	64	64	8	8	1	4	4	3.54	4.87	0.99	34.01
256	512	32	16	16	1	4	2	3.51	4.87	0.99	32.96
256	128	64	16	16	1	4	4	3.54	4.87	0.99	32.91

256	512	64	16	16	1	4	2	3.51	4.87	0.99	32.18
256	256	64	16	16	1	4	2	3.54	4.87	0.99	32.22
256	64	64	16	16	1	2	4	3.59	4.87	0.99	41.98
256	128	64	16	8	1	4	2	3.55	4.87	0.99	32.51
256	512	128	8	32	1	4	4	3.53	4.87	0.99	32.19
256	512	64	32	32	1	4	4	3.52	4.88	0.99	32.13
256	512	32	32	32	1	4	4	3.52	4.88	0.99	31.79
256	512	128	8	8	1	4	2	3.52	4.88	0.99	32.10
256	512	128	16	8	1	4	2	3.53	4.88	0.99	32.44
256	512	128	16	32	1	4	4	3.53	4.88	0.99	32.36
256	64	64	16	16	1	2	1	3.62	4.88	0.99	35.29
256	128	32	16	16	1	4	2	3.56	4.88	0.99	31.74
256	512	64	32	16	1	4	2	3.53	4.88	0.99	32.46
256	512	32	32	16	1	4	2	3.53	4.88	0.99	32.73
256	64	32	16	16	1	2	4	3.60	4.89	0.99	41.82
256	256	32	8	8	1	4	8	3.53	4.89	0.99	32.72
256	512	128	32	8	1	4	2	3.54	4.89	0.99	32.80
256	256	64	8	8	1	4	8	3.54	4.89	0.99	33.05
256	128	64	8	16	1	4	2	3.56	4.89	0.99	32.33
256	512	128	8	16	1	4	2	3.54	4.89	0.99	32.30
256	64	64	16	8	1	4	4	3.57	4.89	0.99	34.33
256	512	128	16	16	1	4	2	3.54	4.89	0.99	32.59
256	512	128	32	32	1	4	4	3.54	4.90	0.99	32.57
256	256	32	16	8	1	4	8	3.54	4.90	0.99	32.80
256	512	64	8	32	1	4	2	3.54	4.90	0.99	32.00
256	512	32	8	32	1	4	2	3.53	4.90	0.99	32.42
256	256	64	16	8	1	4	8	3.55	4.90	0.99	33.16
256	512	64	16	32	1	4	2	3.54	4.90	0.99	32.29
256	512	32	16	32	1	4	2	3.54	4.90	0.99	32.45
256	512	128	32	16	1	4	2	3.56	4.91	0.99	32.90
256	128	64	16	16	1	4	2	3.58	4.91	0.99	32.56
256	64	64	8	8	1	4	2	3.58	4.91	0.99	32.98
256	256	32	8	8	1	4	1	3.55	4.91	0.99	33.94
256	128	32	8	8	1	4	1	3.57	4.91	0.99	32.79
256	256	32	16	8	1	4	1	3.56	4.91	0.99	33.68
256	256	32	8	16	1	4	8	3.55	4.91	0.99	32.91
256	128	32	8	8	1	4	8	3.56	4.92	0.99	34.72
256	512	64	32	32	1	4	2	3.56	4.92	0.99	32.58
256	64	32	16	16	1	4	4	3.58	4.92	0.99	34.20
256	64	64	8	16	1	4	4	3.58	4.92	0.99	34.44
256	256	64	8	16	1	4	8	3.56	4.92	0.99	33.08
256	512	32	32	32	1	4	2	3.56	4.92	0.99	32.25
256	512	128	8	32	1	4	2	3.56	4.92	0.99	32.53
256	512	128	16	32	1	4	2	3.57	4.92	0.99	32.77
256	256	64	8	8	1	4	1	3.57	4.92	0.99	32.61
256	256	32	16	16	1	4	8	3.56	4.92	0.99	32.98
256	128	32	16	8	1	4	1	3.59	4.92	0.99	32.35
256	256	64	16	8	1	4	1	3.58	4.93	0.99	32.80
256	128	64	8	8	1	4	8	3.58	4.93	0.99	34.92
256	256	64	16	16	1	4	8	3.57	4.93	0.99	33.22

256	256	32	8	16	1	4	1	3.57	4.93	0.99	33.25
256	128	32	16	8	1	4	8	3.58	4.93	0.99	34.95
256	64	64	16	8	1	4	2	3.61	4.93	0.99	33.32
256	512	128	32	32	1	4	2	3.58	4.94	0.99	33.01
256	256	32	16	16	1	4	1	3.58	4.94	0.99	33.07
256	128	64	8	8	1	4	1	3.60	4.94	0.99	32.92
256	256	64	8	16	1	4	1	3.59	4.94	0.99	32.58
256	64	64	16	16	1	4	4	3.60	4.94	0.99	34.73
256	512	64	16	8	1	4	1	3.57	4.94	0.99	34.20
256	256	64	16	16	1	4	1	3.59	4.94	0.99	32.85
256	128	64	16	8	1	4	8	3.59	4.94	0.99	35.08
256	128	32	8	16	1	4	1	3.60	4.94	0.99	32.01
256	512	32	16	8	1	4	1	3.56	4.95	0.99	35.30
256	512	64	32	8	1	4	1	3.58	4.95	0.99	33.90
256	64	32	16	16	1	4	2	3.62	4.95	0.99	32.66
256	512	64	8	8	1	4	1	3.56	4.95	0.99	34.18
256	128	64	16	8	1	4	1	3.61	4.95	0.99	33.15
256	512	32	8	8	1	4	1	3.56	4.95	0.99	35.41
256	512	32	32	8	1	4	1	3.58	4.95	0.99	34.79
256	128	32	8	16	1	4	8	3.59	4.95	0.99	35.10
256	512	64	16	16	1	4	1	3.58	4.95	0.99	33.77
256	64	64	8	16	1	4	2	3.62	4.96	0.99	33.39
256	128	32	16	16	1	4	1	3.62	4.96	0.99	32.18
256	512	64	8	16	1	4	1	3.58	4.96	0.99	33.74
256	512	64	32	16	1	4	1	3.59	4.96	0.99	33.53
256	512	32	16	16	1	4	1	3.58	4.96	0.99	34.77
256	512	32	8	16	1	4	1	3.57	4.96	0.99	34.86
256	512	128	16	8	1	4	1	3.59	4.96	0.99	33.29
256	128	64	8	16	1	4	8	3.60	4.96	0.99	35.24
256	512	128	32	8	1	4	1	3.60	4.97	0.99	33.47
256	512	32	32	16	1	4	1	3.59	4.97	0.99	34.34
256	128	64	8	16	1	4	1	3.63	4.97	0.99	32.90
256	128	32	16	16	1	4	8	3.60	4.97	0.99	35.34
256	512	128	8	8	1	4	1	3.59	4.97	0.99	33.17
256	512	128	16	16	1	4	1	3.60	4.97	0.99	33.42
256	512	128	32	16	1	4	1	3.61	4.97	0.99	33.54
256	512	64	16	32	1	4	1	3.60	4.98	0.99	33.19
256	128	64	16	16	1	4	1	3.64	4.98	0.99	33.10
256	512	128	8	16	1	4	1	3.60	4.98	0.99	33.37
256	64	64	16	16	1	4	2	3.64	4.98	0.99	33.76
256	512	64	8	32	1	4	1	3.60	4.98	0.99	33.23
256	128	64	16	16	1	4	8	3.62	4.98	0.99	35.41
256	512	64	32	32	1	4	1	3.61	4.98	0.99	33.23
256	512	32	16	32	1	4	1	3.60	4.98	0.99	34.06
256	512	32	8	32	1	4	1	3.60	4.99	0.99	34.11
256	512	128	16	32	1	4	1	3.62	4.99	0.99	33.52
256	512	32	32	32	1	4	1	3.62	4.99	0.99	33.71
256	512	128	8	32	1	4	1	3.62	4.99	0.99	33.54
256	512	128	32	32	1	4	1	3.63	4.99	0.99	33.60
256	64	64	8	8	1	4	1	3.65	4.99	0.99	33.23

256	64	64	8	8	1	4	8	3.64	5.00	0.99	37.06
256	256	64	8	8	1	2	8	3.67	5.00	0.99	42.45
256	256	64	16	8	1	2	8	3.68	5.01	0.99	42.57
256	64	64	16	8	1	4	1	3.67	5.01	0.99	33.38
256	64	32	16	16	1	4	1	3.68	5.02	0.99	32.48
256	64	64	16	8	1	4	8	3.65	5.02	0.99	37.13
256	256	64	8	16	1	2	8	3.69	5.02	0.99	42.79
256	256	64	16	16	1	2	8	3.69	5.02	0.99	42.90
256	256	32	16	8	1	2	8	3.69	5.03	0.99	42.70
256	256	32	8	8	1	2	8	3.69	5.03	0.99	42.53
256	64	64	8	16	1	4	1	3.68	5.03	0.99	33.24
256	64	64	16	16	1	4	1	3.70	5.04	0.99	33.59
256	256	32	8	16	1	2	8	3.70	5.04	0.99	42.92
256	64	64	8	16	1	4	8	3.67	5.04	0.99	37.32
256	256	32	16	16	1	2	8	3.71	5.04	0.99	43.06
256	64	32	16	16	1	4	8	3.68	5.06	0.99	37.66
256	64	64	16	16	1	4	8	3.68	5.06	0.99	37.42
256	128	64	8	8	1	2	8	3.73	5.07	0.99	45.46
256	128	64	16	8	1	2	8	3.73	5.08	0.99	45.53
256	128	64	8	16	1	2	8	3.75	5.09	0.99	45.82
256	128	32	8	8	1	2	8	3.74	5.09	0.99	45.79
256	128	32	16	8	1	2	8	3.75	5.10	0.99	45.91
256	128	64	16	16	1	2	8	3.75	5.10	0.99	45.86
256	128	32	8	16	1	2	8	3.76	5.12	0.99	46.20
256	128	32	16	16	1	2	8	3.77	5.12	0.99	46.28
256	64	64	8	8	1	2	8	3.80	5.17	0.99	48.43
256	64	64	16	8	1	2	8	3.81	5.18	0.99	48.40
256	64	64	8	16	1	2	8	3.83	5.20	0.99	48.76
256	64	64	16	16	1	2	8	3.83	5.20	0.99	48.70
256	64	32	16	16	1	2	8	3.86	5.24	0.99	49.55
256	512	32	8	8	1	1	1	3.94	5.28	0.99	35.88
256	512	64	8	8	1	1	1	3.94	5.28	0.99	35.98
256	512	128	8	8	1	1	1	3.95	5.29	0.99	36.06
256	512	32	16	8	1	1	1	3.96	5.31	0.99	35.93
256	512	64	16	8	1	1	1	3.97	5.31	0.99	36.00
256	256	32	8	8	1	1	1	3.97	5.31	0.99	36.11
256	256	64	8	8	1	1	1	3.98	5.31	0.99	36.14
256	512	128	16	8	1	1	1	3.97	5.31	0.99	36.05
256	512	32	8	16	1	1	1	3.97	5.32	0.99	36.07
256	512	64	8	16	1	1	1	3.98	5.33	0.99	36.15
256	512	128	8	16	1	1	1	3.98	5.33	0.99	36.21
256	512	128	8	8	1	1	2	3.99	5.33	0.99	40.19
256	512	64	8	8	1	1	2	3.98	5.33	0.99	39.53
256	512	32	32	8	1	1	1	3.99	5.34	0.99	35.91
256	256	32	16	8	1	1	1	4.00	5.34	0.99	36.07
256	128	32	8	8	1	1	1	4.01	5.34	0.99	36.07
256	128	64	8	8	1	1	1	4.01	5.34	0.99	36.08
256	512	64	32	8	1	1	1	4.00	5.34	0.99	35.95
256	512	32	8	8	1	1	2	3.99	5.34	0.99	38.98
256	256	64	16	8	1	1	1	4.00	5.34	0.99	36.08

256	512	128	32	8	1	1	1	4.00	5.35	0.99	35.98
256	512	32	16	16	1	1	1	4.00	5.35	0.99	36.11
256	512	128	16	8	1	1	2	4.00	5.35	0.99	40.56
256	512	64	16	8	1	1	2	4.00	5.35	0.99	39.91
256	512	64	16	16	1	1	1	4.00	5.35	0.99	36.16
256	256	64	8	8	1	1	2	4.00	5.35	0.99	41.54
256	512	128	16	16	1	1	1	4.00	5.35	0.99	36.20
256	512	32	16	8	1	1	2	4.00	5.35	0.99	39.37
256	256	32	8	8	1	1	2	4.00	5.36	0.99	40.91
256	256	32	8	16	1	1	1	4.01	5.36	0.99	36.26
256	256	64	8	16	1	1	1	4.01	5.36	0.99	36.28
256	512	128	8	16	1	1	2	4.01	5.36	0.99	40.78
256	512	64	8	16	1	1	2	4.01	5.36	0.99	40.11
256	512	128	32	8	1	1	2	4.02	5.37	0.99	40.96
256	256	64	16	8	1	1	2	4.02	5.37	0.99	41.93
256	64	64	8	8	1	1	1	4.04	5.37	0.99	35.79
256	512	64	32	8	1	1	2	4.02	5.37	0.99	40.32
256	512	32	8	16	1	1	2	4.01	5.37	0.99	39.54
256	128	32	16	8	1	1	1	4.03	5.37	0.99	35.95
256	128	64	16	8	1	1	1	4.03	5.37	0.99	35.95
256	256	32	16	8	1	1	2	4.02	5.37	0.99	41.32
256	512	128	16	16	1	1	2	4.02	5.37	0.99	41.10
256	512	32	32	8	1	1	2	4.02	5.37	0.99	39.81
256	512	64	16	16	1	1	2	4.02	5.37	0.99	40.45
256	512	32	8	32	1	1	1	4.02	5.37	0.99	36.24
256	512	32	32	16	1	1	1	4.02	5.38	0.99	36.07
256	512	64	8	32	1	1	1	4.02	5.38	0.99	36.30
256	512	64	32	16	1	1	1	4.03	5.38	0.99	36.11
256	512	128	8	32	1	1	1	4.02	5.38	0.99	36.34
256	512	32	16	16	1	1	2	4.02	5.38	0.99	39.90
256	128	64	8	8	1	1	2	4.03	5.38	0.99	43.89
256	256	32	16	16	1	1	1	4.03	5.38	0.99	36.22
256	256	64	8	16	1	1	2	4.03	5.38	0.99	42.15
256	512	128	32	16	1	1	1	4.03	5.38	0.99	36.13
256	256	64	16	16	1	1	1	4.04	5.39	0.99	36.22
256	128	32	8	8	1	1	2	4.03	5.39	0.99	43.16
256	256	32	8	16	1	1	2	4.03	5.39	0.99	41.50
256	128	32	8	16	1	1	1	4.05	5.39	0.99	36.20
256	128	64	8	16	1	1	1	4.05	5.39	0.99	36.20
256	512	128	32	16	1	1	2	4.04	5.39	0.99	41.47
256	512	64	32	16	1	1	2	4.04	5.39	0.99	40.84
256	512	32	16	32	1	1	1	4.04	5.40	0.99	36.26
256	512	128	8	32	1	1	2	4.04	5.40	0.99	41.41
256	512	64	16	32	1	1	1	4.04	5.40	0.99	36.32
256	512	64	8	32	1	1	2	4.04	5.40	0.99	40.73
256	256	64	16	16	1	1	2	4.04	5.40	0.99	42.51
256	512	32	32	16	1	1	2	4.04	5.40	0.99	40.33
256	64	64	16	8	1	1	1	4.06	5.40	0.99	35.62
256	64	32	16	8	1	1	1	4.06	5.40	0.99	35.58
256	512	128	16	32	1	1	1	4.04	5.40	0.99	36.34

256	128	64	16	8	1	1	2	4.05	5.40	0.99	44.29
256	256	32	16	16	1	1	2	4.05	5.40	0.99	41.89
256	128	32	16	8	1	1	2	4.05	5.41	0.99	43.58
256	512	32	8	32	1	1	2	4.04	5.41	0.99	40.14
256	512	128	16	32	1	1	2	4.05	5.41	0.99	41.69
256	512	64	16	32	1	1	2	4.05	5.41	0.99	41.03
256	128	32	16	16	1	1	1	4.07	5.42	0.99	36.09
256	512	32	16	32	1	1	2	4.05	5.42	0.99	40.47
256	128	64	16	16	1	1	1	4.07	5.42	0.99	36.09
256	128	64	8	16	1	1	2	4.06	5.42	0.99	44.55
256	64	64	8	16	1	1	1	4.08	5.42	0.99	35.93
256	512	32	32	32	1	1	1	4.06	5.42	0.99	36.22
256	128	32	8	16	1	1	2	4.06	5.43	0.99	43.80
256	512	128	32	32	1	1	2	4.07	5.43	0.99	42.04
256	512	64	32	32	1	1	1	4.07	5.43	0.99	36.27
256	512	64	32	32	1	1	2	4.06	5.43	0.99	41.41
256	64	64	8	8	1	1	2	4.08	5.43	0.99	46.56
256	512	128	32	32	1	1	1	4.07	5.43	0.99	36.28
256	512	32	32	32	1	1	2	4.07	5.44	0.99	40.89
256	128	64	16	16	1	1	2	4.08	5.44	0.99	44.92
256	128	32	16	16	1	1	2	4.08	5.44	0.99	44.20
256	64	64	16	16	1	1	1	4.10	5.45	0.99	35.77
256	64	32	16	16	1	1	1	4.10	5.45	0.99	35.73
256	64	64	16	8	1	1	2	4.10	5.46	0.99	46.96
256	64	64	8	16	1	1	2	4.11	5.48	0.99	47.26
256	64	64	16	16	1	1	2	4.13	5.50	0.99	47.62
256	64	32	16	16	1	1	2	4.13	5.50	0.99	46.86
256	512	128	8	8	1	1	4	4.14	5.56	0.99	49.47
256	512	64	8	8	1	1	4	4.14	5.56	0.99	49.22
256	512	128	16	8	1	1	4	4.15	5.56	0.99	49.88
256	512	64	16	8	1	1	4	4.14	5.56	0.99	49.56
256	512	32	8	8	1	1	4	4.15	5.57	0.99	49.16
256	512	128	32	8	1	1	4	4.15	5.57	0.99	50.38
256	512	64	32	8	1	1	4	4.15	5.57	0.99	50.11
256	512	32	16	8	1	1	4	4.15	5.57	0.99	49.52
256	512	128	8	16	1	1	4	4.16	5.57	0.99	49.91
256	512	64	8	16	1	1	4	4.15	5.57	0.99	49.60
256	256	64	8	8	1	1	4	4.16	5.57	0.99	51.94
256	512	128	16	16	1	1	4	4.16	5.57	0.99	50.26
256	512	64	16	16	1	1	4	4.16	5.57	0.99	49.94
256	512	32	32	8	1	1	4	4.16	5.58	0.99	49.98
256	256	32	8	8	1	1	4	4.16	5.58	0.99	51.58
256	256	64	16	8	1	1	4	4.16	5.58	0.99	52.42
256	512	128	32	16	1	1	4	4.17	5.58	0.99	50.71
256	512	32	8	16	1	1	4	4.16	5.58	0.99	49.53
256	512	64	32	16	1	1	4	4.17	5.58	0.99	50.45
256	512	32	16	16	1	1	4	4.16	5.58	0.99	49.82
256	256	32	16	8	1	1	4	4.17	5.58	0.99	52.13
256	512	32	32	16	1	1	4	4.17	5.59	0.99	50.27
256	256	64	8	16	1	1	4	4.17	5.59	0.99	52.43

256	512	128	8	32	1	1	4	4.18	5.59	0.99	50.43
256	512	64	8	32	1	1	4	4.18	5.60	0.99	50.09
256	512	128	16	32	1	1	4	4.18	5.60	0.99	50.70
256	256	64	16	16	1	1	4	4.18	5.60	0.99	52.84
256	512	64	16	32	1	1	4	4.18	5.60	0.99	50.40
256	256	32	8	16	1	1	4	4.18	5.60	0.99	52.10
256	512	128	32	32	1	1	4	4.19	5.61	0.99	51.09
256	256	32	16	16	1	1	4	4.18	5.61	0.99	52.56
256	512	32	8	32	1	1	4	4.18	5.61	0.99	49.99
256	512	64	32	32	1	1	4	4.19	5.61	0.99	50.85
256	512	32	16	32	1	1	4	4.19	5.61	0.99	50.19
256	512	32	32	32	1	1	4	4.20	5.62	0.99	50.68
256	128	64	8	8	1	1	4	4.19	5.62	0.99	55.65
256	128	32	8	8	1	1	4	4.19	5.62	0.99	55.33
256	128	64	16	8	1	1	4	4.20	5.63	0.99	56.10
256	128	32	16	8	1	1	4	4.20	5.63	0.99	55.86
256	128	64	8	16	1	1	4	4.22	5.65	0.99	56.16
256	128	32	8	16	1	1	4	4.22	5.65	0.99	55.87
256	128	64	16	16	1	1	4	4.22	5.66	0.99	56.54
256	128	32	16	16	1	1	4	4.23	5.66	0.99	56.32
256	64	64	8	8	1	1	4	4.25	5.70	0.99	59.81
256	64	64	16	8	1	1	4	4.26	5.72	0.99	60.19
256	64	64	8	16	1	1	4	4.28	5.73	0.99	60.31
256	64	64	16	16	1	1	4	4.29	5.75	0.99	60.62
256	64	32	16	16	1	1	4	4.29	5.75	0.99	60.63
256	256	64	8	8	1	1	8	4.47	6.04	0.99	61.56
256	256	64	16	8	1	1	8	4.47	6.04	0.99	61.82
256	256	32	8	8	1	1	8	4.47	6.04	0.99	61.74
256	256	32	16	8	1	1	8	4.47	6.04	0.99	61.95
256	256	64	8	16	1	1	8	4.48	6.05	0.99	61.88
256	256	64	16	16	1	1	8	4.48	6.05	0.99	62.12
256	256	32	8	16	1	1	8	4.48	6.05	0.99	62.00
256	256	32	16	16	1	1	8	4.49	6.05	0.99	62.17
256	128	64	8	8	1	1	8	4.52	6.10	0.99	65.03
256	128	32	8	8	1	1	8	4.52	6.11	0.99	65.01
256	128	64	16	8	1	1	8	4.52	6.11	0.99	65.29
256	128	32	16	8	1	1	8	4.52	6.11	0.99	65.32
256	128	64	8	16	1	1	8	4.53	6.12	0.99	65.38
256	128	32	8	16	1	1	8	4.53	6.12	0.99	65.39
256	128	64	16	16	1	1	8	4.53	6.13	0.99	65.60
256	128	32	16	16	1	1	8	4.54	6.13	0.99	65.65
256	64	64	8	8	1	1	8	4.60	6.21	0.99	68.36
256	64	64	16	8	1	1	8	4.60	6.22	0.99	68.55
256	64	64	8	16	1	1	8	4.61	6.23	0.99	68.68
256	64	64	16	16	1	1	8	4.62	6.24	0.99	68.83
256	64	32	16	16	1	1	8	4.62	6.25	0.99	69.12

Supplementary Table 5. Optimization of kernel weights and GPR parameters (σ_n and ζ) for multi-scale kernel prediction of ^{13}C chemical shifts. The optimization was carried out on the CSD-2k set, using 3-fold cross validation. For each configuration are reported the corresponding mean absolute error (MAE), root-mean-square error (RMSE), the R-squared (R^2) coefficient and the supremum (SUP). In bold is shown the set of parameters that we selected.

Multi-Scale Kernel Weights						ζ	σ_n	MAE (ppm)	RMSE (ppm)	R^2	SUP (ppm)
r_c =2Å	r_c =3Å	r_c =4Å	r_c =5Å	r_c =6Å	r_c =7Å						
**256**	**128**	**32**	**8**	**8**	**1**	**2**	**0.1**	**9.96**	**14.25**	**0.99**	**132.00**
256	128	32	8	8	1	2	1	9.95	14.28	0.99	133.20
256	128	32	8	8	1	4	1	9.67	14.35	0.98	137.10
256	128	32	8	8	1	4	0.1	9.74	14.36	0.98	135.69
256	128	32	8	8	1	2	0.01	10.36	14.72	0.98	133.68
256	128	32	8	8	1	4	0.01	10.34	15.12	0.98	141.99
256	128	32	8	8	1	1	1	11.68	15.94	0.98	115.98
256	128	32	8	8	1	1	0.1	14.59	19.36	0.97	114.82
256	128	32	8	8	1	1	0.01	14.64	19.43	0.97	118.61

Supplementary Table 6. Optimization of kernel weights and GPR parameters (σ_n and ζ) for multi-scale kernel prediction of ^{15}N chemical shifts. The optimization was carried out on the CSD-2k set, using 3-fold cross validation. For each configuration are reported the corresponding mean absolute error (MAE), root-mean-square error (RMSE), the R-squared (R^2) coefficient and the supremum (SUP). In bold is shown the set of parameters that we selected.

Multi-Scale Kernel Weights						ζ	σ_n	MAE (ppm)	RMSE (ppm)	R^2	SUP (ppm)
r_c =2Å	r_c =3Å	r_c =4Å	r_c =5Å	r_c =6Å	r_c =7Å						
**256**	**128**	**32**	**8**	**8**	**1**	**2**	**5**	**14.80**	**20.09**	**0.99**	**117.92**
256	128	32	8	8	1	2	10	14.62	20.27	0.99	154.74
256	128	32	8	8	1	4	10	14.67	20.35	0.99	145.67
256	128	32	8	8	1	4	5	14.84	20.39	0.99	132.32
256	128	32	8	8	1	4	2	14.97	20.49	0.99	129.65
256	128	32	8	8	1	2	2	15.26	20.52	0.99	110.01
256	128	32	8	8	1	4	1	15.04	20.54	0.99	127.63
256	128	32	8	8	1	2	1	15.43	20.68	0.99	108.26
256	128	32	8	8	1	1	5	16.22	22.20	0.99	149.10
256	128	32	8	8	1	1	10	16.27	23.00	0.99	201.11
256	128	32	8	8	1	1	2	17.55	23.52	0.99	141.85
256	128	32	8	8	1	1	1	19.43	25.78	0.98	147.58

Supplementary Table 7. Optimization of kernel weights and GPR parameters (σ_n and ζ) for multi-scale kernel prediction of ^{17}O chemical shifts. The optimization was carried out on the CSD-2k set, using 3-fold cross validation. For each configuration are reported the corresponding mean absolute error (MAE), root-mean-square error (RMSE), the R-squared (R^2) coefficient and the supremum (SUP). In bold is shown the set of parameters that we selected.

Comparison to Experiments

Comparison between ^1H experimental chemical shifts and ^1H chemical shifts calculated with ShiftML were carried out analysing 68 chemical shifts obtained from 6 crystal structures. The names, IUPAC IDs, CSD reference codes (when available) and references to the experimental NMR data of the analysed crystal structures are the following:

(i) Naproxen, (2S)-2-(6-Methoxy-2-naphthyl)propanoic acid, COYRUD11, Ref. 22
(ii) Uracil, Pyrimidine-2,4(1H,3H)-dione, URACIL, Ref. 23
(iii) Co-crystal of 3,5-dimethylimidazole and 4,5-dimethylimidazole, Ref. 24
(iv) Theophylline, 1,3-Dimethyl-3,7-dihydro-1H-purine-2,6-dione, BAPLOT01, Ref. 3
(v) Cocaine, methyl (1R,2R,3S,5S)-3- (benzoyloxy)-8-methyl-8-azabicyclo[3.2.1] octane-2-carboxylate, COCAIN10, Ref. 3
(vi) AZD8329, 4-[4-(2-adamantylcarbamoyl)-5-tert-butylpyrazol-1-yl]benzoic acid, Ref. 4

The crystal structures (i-iv) were obtained from Ref. 25, where the experimentally determined crystal structures were subjected to all-atom geometry optimization with fixed lattice parameters, as described in the reference. Crystal structures (v) and (vi) were obtained from Refs. [3] and [4] respectively. All the used crystal structures are given in the Supplementary Dataset 8.

We used assigned chemical shift values and we account for rotational dynamics of the methyl groups by averaging the chemical shift values of the three ^1H positions to a single value for each methyl group. The calculated chemical shieldings σ are converted to the corresponding chemical shifts δ through the relationship $\delta = \sigma_{ref} - \beta\sigma$ (9). For each structure, we calculated the value of σ_{ref} and β by a linear regression between calculated and experimental shifts. The calculations were carried out in MATLAB using a home-written script. The chemical structures, together with the assigned experimental chemical shifts and the parameters for conversion between shieldings and shifts are shown in Supplementary Figure 9 and Supplementary Table 8.

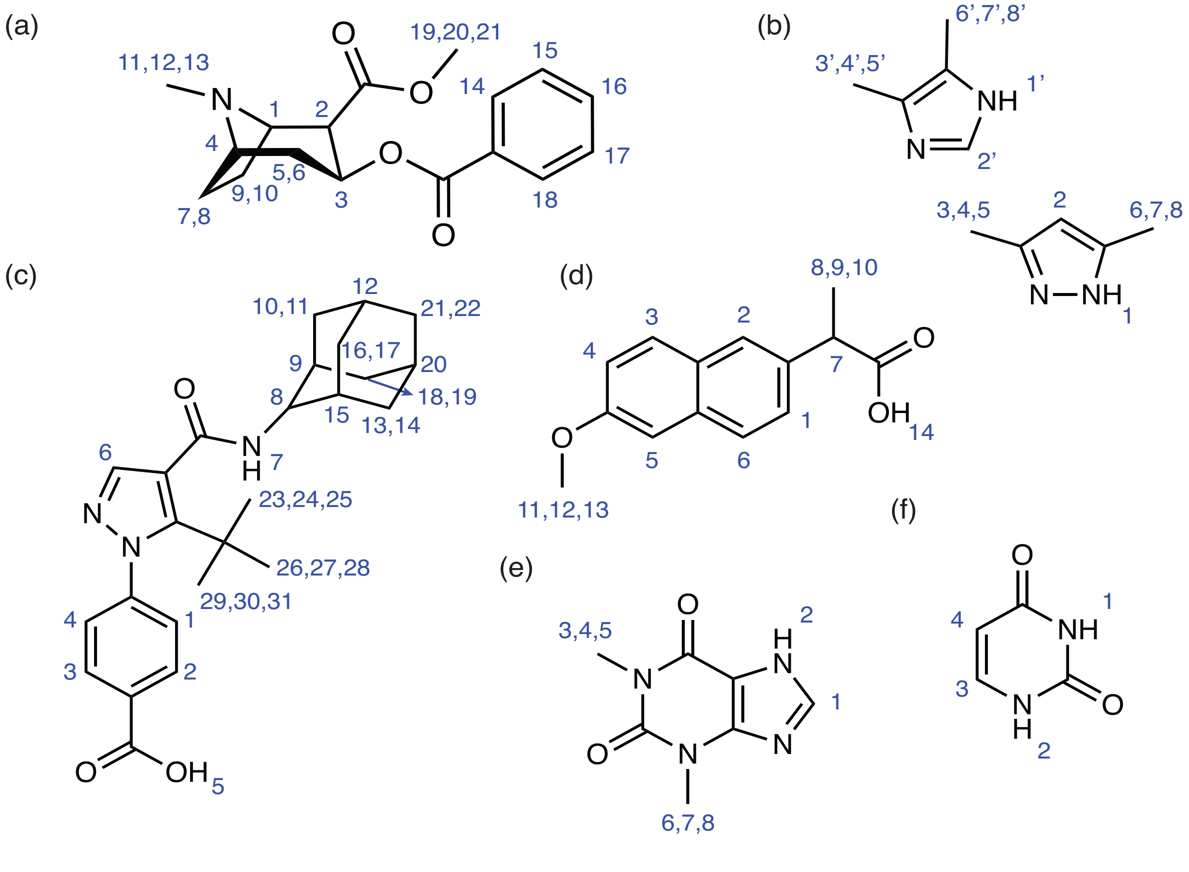

Supplementary Figure 9. Chemical structures of the compounds used for experimental comparison. In order, cocaine (a), 3,5-dimethylimidazole and 4,5-dimethylimidazole (b), AZD8329 (c), naproxen (d), theophylline (e) and uracil (f), and the labelling scheme used here.

Naproxen

Atom Label	Experimental ^1H δ (ppm)	ShiftML ^1H δ (ppm)
1	7	6.87
2	6.1	6.07
3	3.8	3.74
4	4.5	4.40
5	4.1	4.51
6	5.9	5.11
7	3.2	3.15
8,9,10	1.8	1.98
11,12,13	2.3	2.63
14	11.5	11.74
σ_{ref} 25.38	β	0.81

Uracil

Atom Label	Experimental ^1H δ (ppm)	ShiftML ^1H δ (ppm)
3	7.5	7.76
2	10.8	10.68
1	11.2	11.22
4	6	5.85
σ_{ref} 23.71	β	0.74

3,5-dimethylimidazole & 4,5-dimethylimidazole

Atom Label	Experimental ^1H δ (ppm)	ShiftML ^1H δ (ppm)
2’	4.8	5.17
6’,7’,8’	0.7	0.77
3’,4’,5’	1.4	0.91
1’	13	12.55
6’,7’,8’	1.4	1.20
3’,4’,5’	1.5	1.35
1’	15	14.92
2’	5.2	6.14
σ_{ref} 29.91	β	0.99

Theophylline

Atom Label	Experimental ^1H δ (ppm)	ShiftML ^1H δ (ppm)
2	14.6	14.57
1	7.7	7.27
3,4,5	3.4	3.22
6,7,8	3.4	3.52
σ_{ref} 25.98	β	0.83

Cocaine

Atom Label	Experimental ^1H δ (ppm)	ShiftML ^1H δ (ppm)
1	3.76	3.95
2	3.78	3.22
3	5.63	6.11
4	3.32	3.73
5	3.06	2.55
6	3.49	2.99
7	2.91	2.69
8	3.38	3.18
9	2.56	2.44
10	2.12	2.37
11,12,13	1.04	1.80

AZD8329

Atom Label	Experimental ^1H δ (ppm)	ShiftML ^1H δ (ppm)
1	6.92	6.53
2	8.69	7.85
3	9.01	9.35
4	8.47	7.91
5	15.37	15.95
6	7.73	7.60
7	9.64	9.37
8	2.90	2.79
9	1.78	1.98
10	1.88	1.79
11	1.88	2.61

14	8.01	8.40	12	1.8	1.68
15	8.01	7.39	13	1.6	1.28
15	8.01	7.66	14	0.44	0.87
17	8.01	8.09	15	1.54	1.94
18	8.01	8.03	16	1.88	2.76
19,20,21	3.78	4.28	17	1.88	1.69
			18	0.8	1.21
			19	0.8	0.43
			20	1	1.42
			21	1.74	1.47
			22	1.74	1.21
			23,24,25	0.73	0.84
			26,27,28	0.73	1.02
			29,30,31	0.73	0.14
σ_{ref} 30.04	β	0.96	σ_{ref} 28.39	β	0.91

Supplementary Table 8. Experimental and calculated chemical shifts of naproxen, uracil, the co-crystal of 3,5-dimethylimidazole and 4,5-dimethylimidazole, theophylline, cocaine and AZD8329. The labelling scheme is given in Supplementary Figure 3. When more than one atom corresponds to a single chemical shift value, their values were averaged

Supplementary Note 1: CSD-500 set

The CSD-500 set contains the structures with the following CSD Refcodes:
COBHUW, QUGWUK, MELYUY, RAVFOK, UMUKUJ, TAVJAD, DOMNEY, QEXKUA, KOGWUZ, HAMTIZ, MAHPUJ, POLJEF, DXCYTD, BUFNEV01, COWPUZ, VIMKOT, ASPARM10, JIPCUG10, AZOBEN12, GUJGEX, XOWJUP, NUYWUB, RECYIH, XOHMAI, UJUKIT, MEYBIB, POQSEU, ENIMET, QOMVUK, AHOWOL, YIMPOB, LEVSIO, PANLEZ10, WIQZOL, CORTPY, FOSLEG, CINCHO10, NOPHKN, UWEZED, RUSGOD, MENDAL01, NEQPEG, MATQOO, HODKEQ, MEHLER, EDAXOW, FADHOJ, ROHJED, KOFKAR, YAZDEI, KABKIJ, XUJKUK, OXUJUN, QUWFIZ, VAFPAV, ITINEG, LILDEP, VONNOB, AROKUN, HUVWOL, NEZFON, ONBZAM, VOGDIE, IDURIJ, WEPTIV01, CBMZPN21, AMEXOH, IVEZAK, EFIBAX, MOSLAI, AXADAF, MOBNUM, RIQSER, DASNIV, ROGRIQ, YODXAS, MEJDOU, COCYAW, SOMNIT, KUZJIA, BZAMID08, THYDIN05, XINHIL, TALVAD, SEGROL, FUPWES, VIDMAX02, YEHWUD, NANJIW, MEHNAP, DAFTAF, ITIREI, FACZIU, TOPRIB, AZIDES, ROJXOD, QIYLAM, ZEYLAS, UQOLIW, VANFEV, ZOFNUD, HUDHEU, PIHBOZ, VOCHUR, LIXQEO, SAWVET, MUBBAN, XAZYIG, ODOROO, IBOPIA, YOCWUK, KEMFIS, BUMNOM, EWOBIB, WEGPEF, WECZEJ, RULHOX, FELDOR, BUZJIR, VUHZEE, DILDUZ, AXOSOW03, ANOSAY, PACWAU, YODPAJ, QECNAP, PUMSEV, ZZZFFY01, DAJZEU, ZEMHOO, EKAHOP, PMPZOL, FIHLEO, AKUBIT, NUZPUT01, ARONOM, BEGDIB01, UNURIF, IMEQUO, IQIZEO, SIMYOE, FESNEW05, RIFZAI, COSPEG, PELXAG10, UTICIK, RUVPIJ, SUKNIW02, WIFQEI, SEFNOG, HUDYUA, ANAHII, AJIXUM, LUXSAY, ZIYYUD, UBUXOG, RAKTOO, ACRDIN07, EMODUG, IDUJEW, KOTMUB, DUTKOU, QAKDAJ, NORFUW, QQQFDJ20, AFIQUC, SAZFOO, DITZOX, MUJGEE, BIKNUE, TIWZUV, KUYWEH, EABZBU, IQIKOI, OXAROV, MAJJUD, LAFHEH, FALHIJ, XAQTUF, COLBAG, MELAMI05, ISIJIE, EMISUQ, ESESEA, HIMSUS, ZICKIF, GIZRUE, IYASUW, WEVVEZ, KUZQIG, YAWWOK, SIHZAM, CAZCOX, LOPLUZ, EBAXOW, GIDHUW, RIHFIY, EXEWEJ, MODXUZ, BEHWER, VIDDAO, OJICUF, HUVZUV, WUCVIB, SENKUR, EZISUC, PEFGIS, GAWFEQ, NCUBEB10, ORADUH, ZEBXOV, JOHKEY, VEZCUY, IBEHII, REJVAE, XASHUW, NBZOAC11, OFEVOL, JEBQOW, WAFBIQ, WULTUT, BOMSIH, VASLOR, OGIMIC, VINZUP, SOGCUN, DOTFOI, OPIZAQ, KAHJEK, GADSIO, GAQJUF, XEZFUF, ZIKQIT, BAJCIY03, SUWKEC, EVINII, BERSOG, SOYMEZ, OCIPAR, GIZFEB, UNAMOL, TICLIC, ALOSEZ, PIWBUS, AYUNEO, ECODUV, PUNFAH, BOLGOZ, UBASAT, RIMHEC, WAWQUH, MOTNUF, QEPRIO, SAJCAJ, XABFUE, XIMGAB, SEYCUU, DOVWAM, RICTIG, MISDAT, SATPUZ, UQAMIK, KIVZIZ, BASNOZ, CUTCUQ, SORFIQ, TESDOL, EMIPUM, UBUVIY, EVIHUM02, DUZLUF, HOMKIF, IPINIE, RAZVUJ, TEMKAZ, XUVBAT, VUGWAX, HUYYOP, NEVDOH, YERTIZ01, KAKHEL, SAVREN, AHOXOL, IVABEO, JOQTUE, PIJREF, ZAYPOE, WIZWOS, TOPXUT, XINLAJ, COYBOJ, REKMEZ, WOKJOV, BIXQEF, GASXON, HECNOS, RACGEK, EHAHAY, BAYPAT, ZULCEQ, UNUVEF, JAYFOF, BEDLEB01, MAQWIM23, HOMZUG, EPHEDR01, ZIWMOJ, GUFYOX, MBPHOL02, SATPEI02, QIQCOI, CIKSAQ, SUYYIV, RUCNOU, NIQTAJ, HISNII, IJEZUS, NASZAJ, APODUG, DILKIT, GIXKOP, INAVIC, BAPPUF, NURZOP, NUPPOD, RIZBAF, RACGEJ, PEPLAX, SIHCES, SIGSAD, ZOYMOP07, PILFIB, XOFFEF, GOVQOX, XULNOI, YOFTOE, SEZXIG, FEZLUT, AXAWIG, IQUBZA, VEQMUA, FEMGAF, YOXRIO, FEPTID, EXUVUP, PUPBAD01, QQQAMS02, AHATEK, ZZZBPY10, CXMTUN, WOYTAH, AQEYAW, AMUQOQ, ZOGTIY, VORMEV, JOTKIM01, ZOXYOA, QUBQOT, PUMQEV, QUYZIV, NAPTPR, XIYTIJ, GERPOJ01, PEXPEN, HIZHOP, KETYUF, PRMDIN05, KOJTOT, SUHFEH, FAJDEC, BAQNEM, DIZWEQ, EKAWAQ, VOXNOL, DOHPEV, WOBRIP, YIPPOC, QUFJUY, LUDZIT, DORKOK, BZTROP11, VILPUB, FEMXOK, CEDVIS, HUVPAR, MESQOR, IZAKOK, YIXPUR, OMSTER01, IROZIY, OPUTEA, FAHXUH, VEMBAS, TAJSOM, SULROG, NUQLES, LAVSIL, GADVAJ, EVIQEF, XANJUS, HIWYIV, YOWYOY02, ZAVXUP, OCAWOF, GUCJUK, HABNED, LUQDOS, PYAZAC, ONOTIW, TICKAT, POKKAD10, BUGQUQ, ZATDOP, MEJQEY, RULDAF, KEZNUZ, MUTWON, DOLBIR10, HIGCIK01, NOQHAE, NAJLUF, WAGTII, ZOSVEI, IQULUC, EBOVEX, ARUZUK, MALSOH, OTAKAW, QUFCEZ, HAXREE, ZIGBAS, JULGOO, JESHIZ, OHEWOP, AQAGII, ECACER, DENXUP02, LIZHEJ, EKOGAO, AWAVEZ, YUNYUC, BAWRAT, NUKSAO, XESYEA, QAMKEW, KUTKAL, HESTOO, FAHLAB, KUJZIY, WIHBEW, HONKEC, UWOCAM, PEWNIQ, APUPIK, PUWNIG, PHBZAC01, EDIZUM, RUKTAU, YEGGIA, OMABEK, DIWWEN, XACTEC, XAVQOB, VUDDUV, XOTFAN, GUTZOM, SOLGIL, SAFQAU, AFIKAC, POHCAS, EMEFOT, XIZVAD, WIGWOA, RELJAU, ROJHOP, HUMNEK, HODLOC, PUPGUD, ALEXEW, ZEMNAG, YUXCUP, EXEYUD, VETJIO, OWIWUN, EYASAZ, UCANIV, XICCAO, BOPJAS, SEBVAW, XUHZOR, UKUROJ, PEDHAJ, YIDTIQ, EVILEB, ELAWIX, AMHTAR02, OCATOC, PETRAH, BEDFUM, LADNEL.

Quantum Espresso relaxed structures and corresponding chemical shielding are given as a separate xyz file in the Supplementary Dataset 2.

Supplementary Note 2: CSD-2k Set

The CSD-2k set contains the structures with the following CSD Refcodes (in order of FPS selection):
XISJIT, COVSEM, NOETNA02, KESVOT, AMFORM, GUMMUW04, ETDIAM17, LAKRAR, ZUTKEG, CAFMIF, DAFGAS, CUPZEU, NOZKES, RIZFAI, VINYLC, NTROMA01, TILJOP, RIWTIB01, TETZOL,

DOJKUJ, FOKSUT, PARBAC03, UREAOH12, IMAZOL33, DHNAPH17, DAYHAN, EJIQEU02, JOJWOU03, BCBANN06, GLYCIN35, LEPFES, HIXHIF02, NUQPOF, CILBII, OTEBEV, CUZJEO, MOKPUX, MOSGEI, CALFOM, HUQRAM, AMACAM01, CTMTNA06, AJAPIL03, NAYPAF, KUTHAI, DOVCAT, KEMZIL01, AVAKAK, VIWYEH, TAPZAN, KICCOO11, ZIKMOV01, DIOXAZ10, OGIQEB, CITVIL, QAHVUQ, MOMFAV, TAYHOR, GANHUY, GACLEE, CAMGAA, YEKHUT, SIBVUU, VANXIS, QIQKIK, FEDCOI, WEVVOJ01, MEVDAT, XEXVEB, EMUFAU, BECJEY, QOPKUB, FUSVAQ01, LUKCIF, FUNSOW, WIDJOI, FIZZOD, KULMUA, IMAZOL15, ARCLAM03, LUVPAU, UMERAF, NAFZEC, CBOHAZ02, MALIAC02, MUYHIY, COXYAR01, INIJEW, QOXPEZ, MOZSEZ, PODHOH, QQQFGS10, CERMOB, BZDMAZ05, PHGLOL01, ZACSOO, DMDFHZ, LUVTOL, SOJMOT, DUFBOV10, QUDNOU, DAJVUG01, MOQDED, ZDGLPN, GOKNOK, UJEREG01, ZAXLIV, GEFBAV, INEZUY, TEDROL, HYDRZN11, COWHUT, CITSED10, NANQUO02, FAYBUC, CSURCD10, AHEYAO, CALVAM, TIGBIU, FESNEW15, WOSGIU, ZTNONX, UDAYUT01, ZUNTOR, SEDTUQ09, NADHOP01, SAFKAL, CIFROY, ABEWAG, BOQQUT08, PYRTHA10, MELAMI01, KECYBU02, NEPGCL02, FEKVOG, UREAXX27, NACXEU01, TICJAS, EJEHEG, QUVFIX, FOBSEU, MOYHAJ, COTPAC, GEZBAO, MUGNUX, NIKVUZ, PUBMUU23, HEHVAR, IMIWOQ, ACETAC09, EKUJUR, JAPBIL, CUPYUJ, JABCIB, SEQBUN, LETGIA10, MEOXAL, ADALAU, METACM02, CIWMAW01, KEMDOW, OXHEPA, GACGAU, CILWUP11, JEDSUG, TALSOP, EBUKUH, YARZUN03, VOQXAA, FUJTUZ, BONGIW, BICMAD, ACXMPR, ACRLAC03, TETZIB, MEPCHX, IVEZEO, JEXZUH, FEMCEF, AJAQAE, IKIGUE, UREANT03, OFUKOR, XESHOT, DUMQAF, RIZZEH, GOFPUN02, LEGZII, WOCREL, VOBDOF, WUFTUO, MUQHOW, ECADER, BUWCAX, OXAZDO, VIPKIO03, FEXLEZ, YAHCIV, MNPYDO29, RIKPEG, XUSGOI, PORPIN01, LAKDAD, GUHROQ01, JACPOS01, EFUMEY, CIKWOJ, ESIWUZ, YIFXIV, AGUSEB, ATOXAL03, BUKJOG10, ADEZUF, RAVFEA, GLYCNI, APENIU, ZEFPUX01, PIDHIU, GAQWAX, ATZCBX, YOKJOZ, SALOXM09, NUMXAV, WETGAE, NOFBIT, FORRUB, KAXWIQ, DNBOIM, PARGIZ, HEMLAM, QODBOC, ALUQIH, GLYCIN81, ESOYUI, KAYWIR01, LONJAA, QORROF, BIBVAK, OSEXEQ, MENFXN, PECYON, FOHMUK, QIGVAD, TAFKUG, FEHVUK, QONRAO, PELJOG, FORAMO, QIHHIY, YABFEO, EFOBOR, MEUREA, ZERZIH, PYRDHN01, YECVUX, AMTETZ, TURPUR01, DIHIXL10, FEPGEM, RIWJEM, WIHJAZ, SAGLAQ, WEZCAG, HONSIO, KOTWOH, GEJSET, PRMDIN20, HAMCEF, OMIWIQ, AMOXAM, FEPNAP, HIFKUC01, NTRACD, OKEBAJ, OBUPUY, URPRBN10, RIHJIB, AXOSOW, NOHTAZ, PYRDNA06, GIPPAW, IKEGAH, GEXGIZ01, NIPYAZ, JUNQIS, AFZPYM, LETGIA03, TRAZOL01, DZBASK, DUHJIB, AMTETA, IWERUX, PYRAZI06, URICAC, FAFKEC, CYAMPD01, YOVVIP, XOKVIE, IMEGIR09, ZESSOF, KUKHOP, XOCZUL, BUXWIA, GOVZEW01, UDOZUJ, LEVXOB, LUHSIR, CISPOJ, OMIWEM, KUPCOP, BIJJIP, EKUKEC, BATWOK, UNUSED, KUQTIB, NTRGUA01, QAGDAG, ITIPEG, GEDXOD, DUROQN10, YANREM01, BIDJIH, ULEZES, WIKQOX, KABBEW, KENMOF, ZAVTEV, XZBTZO, MAZGAX, HIHJEN, NEPBEQ, PINXAL, ALXANM01, ANIMUH, HOZPET01, XAYYUS, REWHOR, VAZCEG, QAKKOE, IXAQAB, THZTAZ, GELWOL, NBFURX, JEMSUP, NEHJER01, LALNIN28, CASYIG, NUJLUB, ASUWOB01, NOPPOY, BEMYEY, MAMHOX, PUFFOL01, OKUXUP, LABJON01, XIQCEF, ZUNTAD, LAGHOQ, WULYIN, NIBZAM, CREATH04, NEZNAH, EDAWIP, IFUVOW, UTAKUX, LIVZIA, GUMMUW10, IBURAM, BINROF, MTZOPZ10, XIHLIL, VITBAC, YARYAS, JUVMUI, XIBTUY, MEWSUE, VOFDEZ, PYZTAZ, WETFOR, YOTSUV, FONMEB, ONAVEF, GIVFIC, NEBPUF, YIQXAY, PIMELA08, NAXTIQ, AMMALA, EWAMIZ, CPRDCA, CYHEXO, JOHBUD, WABNEV, DEBSOS, HEHQOC, LAYSOV, MOPPUC01, UFORUE, QARWAI, DLGYAH, DUTTAN10, SAPDAQ, JOPNAD, IBOLUG, EXIGAU, DUNVEN, VOFVAN26, EBUKOB, GUKVOX, DOXXAP, HAWYIN, PYRZIN14, ARADEN10, TESDOL01, XADBOW, VEHXAH, JABLUW01, LUSNOC, SIGFOE01, XYLTOL02, VEXREV, HOYMAJ, TUQDUF, NOCNEA, HINXAF, SALOXM04, DOZXEW, PEKSAZ, KUQTEX, MSORAM10, YUZTET, DAZDOZ, QOQGOT, SOJCUP, ITIXUE01, FIBYEW, MUTVEE, TUBYIY, QAJQAV, FUMRAM01, NAPHET, BZAMID06, GUNTEP, OHAHAI, ECARBM10, TEDFEP, SUFHUY, GUHOXM02, BOXBEV, POFLAX01, OXAZIL10, GADZER, HEXDAQ, PUFJIJ, LEGLEQ, DUYREU, GIRRUV, MUDDEU, QIMFAT, SECMOE, ODEJEN, COTTAI, JORBUN, YUWMUY, QUGGAA, HUSTIY, FIBGEE, KAMFAH, SUSYAI, BUFMOG, COWJAB, DUHTUW, UCOTEM, DNITPY, AMACET, HDNBPP, KUHDIA, QEXJIN, CEVGIT, DNPMTA, BZDMAZ16, LACFON, DNPIMZ, YODHIJ01, LESLAX, LENBAH, HIVMOP, AQUHAX, JUVMOC, WOWFUL, THYMIN02, ZIPSIC, OJOLAC, DAVARO, RIVKAJ, JOWWOH, XAVZUP, METHOL04, BZCBUO, JUBSOQ, ZOVHID, BARBAD07, KOVGIL, QOYJOD06, KUQCAB, ACAMOX, ZZZJII01, KIXXEW, HXIBAC, CUMBUH, NEZMUA, KOBYOP, BISMEV10, NOACTD, VUBQAM01, CUZJOY, VODWIU, VONNET, VOFVAN23, OGOKEC, FEHDAX01, ACARBM, FIXPUX, AYAHUE, EMUPAE, GEXMED, TIJKEC, KECPIR, YUSWAM, ACEMID07, FOLVIL, OGAJOV, RATMEF01, ZEXKEU, QARVUB, ZIVTOP, JESNAX, FIMWII, WIGXAN, PUPBAD02, VUCBAW, KEPKIZ, RIGJOG, NURQEW, WEPPUE, LOXBAD, XUDRUL, SAGRUN, LUNYID, LUQPOE01, FOLXIN, POQQIW, DALGON03, OROMIT, TECNUL, NUPTIC, GAFRAJ, ZZZTXI11, GATCAG, PIKYEN, BISKET, GLYCIN18, WOQPEY, TAPHEY, WOLGIN, OREHOK, KEHYAZ, BARBAC01, TOCLUU, PEJBAJ, QAGDEK, KATKIA, HABFUL, TATNBZ, ACETAC01, ZZZRJG01, CDECOL11, CINSOH, TRIRED, PMXCDO10, ZACSII, TROPOL10, IJEPOE, DALQOW, TACQUJ, NMALAM01, ACIDOH, ZZZRTW01, KIJNUM, POWBEI, CPCOHA, PULGOU, IYINAF, MOLMUV, SAWJUW, TIQROC, NIGNOH, BELBUP02, JEMFUD, HUKROU, HEWQOQ, NOCNOK, YUYWEU, METAMI, MEQPAZ, JEZZUJ, UGEBOZ, KUCPED, OXAYAO, DACETA11, BECYAL, UVIZEG, EVIJUP, NPHDZB, UPONAQ, SOQNOC, GESNUN, QEYCOM, ZENMUA, ITEGIX01, KEPFUI, JIXGUS, XENQUD, FAQROE, SERVOA, OJUMIR, WOFVAQ, XELNAE,

VAZMEP, VANCIX, XUDSOG, TEZPDZ10, XACSOL, DOMQUS, SESCAV, CANXIA, KOMHAV, BUVBUR, BAHSUY, TIMCHX, HEWKAW, JIPFAQ, XACCOW, DEBMOM, BUYRUI02, ZOTDAP, KEHWUR, TRURET20, DIPMUK, FENCIK, FOACAM, JUVKAM, HUMNEI, KOWTEV, DIPICA10, XIMKUA, PIZGUA, JOWGAF01, DODCHD, YICMIG, QEKSOP, LSERMH15, KOHMOJ, MUHFOK, MLEICA, CIZSEL, YAGQOM, YIHHON13, PUBLON, YITBOS, JIHVEB, XEPNUD, MUKLIO, DUROQN11, TRMTRZ, DOPLOL05, POWBAE, COGDEH, NIPLED, NILQAC, QIGCIS, FUTFIJ, VOHCAY, JOHKIC, BIZBET, WAJQAC, MFZCHZ, WIKBAT, FEDCUO, IPRPOL03, YALLOO, VIOLME05, GAMGIL, ROBYAI, HABTEJ, CAKZAP, KOVHIM, UPANIJ, MALNAC02, QIWJAI, TAFKAM, BAKFUO, AJEREM, FIBWUI, IQUJEK, HAMCIJ, VASDUO, QONVIZ, ERERZY, COYZEX, FIBXUL, QONRUI, EMIMUI, QOBHIA, KIKVAC01, COHLUI, UCONIJ, LAJZAZ, HOQHAW, CAHOAM, EYOVIX, MTETAC01, CTMTNA12, AXUDED03, BUSFUQ, XESRIW, JINYIP, MISGAX, GEJCAB, TURRAZ, ECIBIB, DOGQUL, KUZWOS, KANQAR01, ISOMUS, JUXLEV, YEKGUS, VUWXAM, EFARIO, QOXMUM, WECCAK, DUSBUQ, LESZIT, VABLIT, TEOXDE01, APITIE, DOYTUG, NRURAM11, VIKVOA, SAGMOE, JUDZIR, YEXNEU01, CILZII, SAWNAH, FEDCIC, NIVBUP, FOZLIQ, DOCMEO, FIHNIV, DETYLE, UKIHON, BPHENO14, FOHFOY, FULPIM, BIGUAN, MIHJIW, MAPCIQ, XEBTEF, RAJDEM, CUFFUG, WOKPAO, KEPSED, YEKJEF, JAXVEK, LIKYOV, NIMFOE02, EDAJIE, NUZKAU, OMIZOB, HUYYAB01, XAJSAE, KOXBOO, ADMNTB04, MIYPEP, LEMVUU, PUQJOC, PUHCOL, AFEXUF, INEZIM, OGIQOL, SAZGOP, UKIHUS, NUFDOJ, QEFBUY01, GOYLOV, SUCCIN, ZERLUD, QEGSOL, CORYIR10, QAFFOS, HIWJIG, BABBUB, TAXTER, FUZYUU, EXUJAI, CAZPUQ, EJEQUF, DMOCDO10, CEZSEF, LEFYUS, VOPTOL, AHUZUZ, RATMIJ, QUDNAG, VIZWEI, QAHCUA, DAYHIV, COKJOB, XEGPII01, MINGEX01, QONROC, NTPYRO12, GOHVUU, XIVBEK, UQAFAV, NENZOX, KIWJIK, YEKJAB, NIFBOT02, OLOBIA, TALJEV, JIWQIQ, BEVXEF01, XANAZH01, TABBEE, UQALUU, IHANUB, CINMER04, TEDNIB, LIJFUG, UHUMEP01, HMHOCN02, DUFDOX10, NEHQAU, KIKTEE, BOCZIE, MAQDAJ, ZOYGUP, TUGSUL, QEXBAX, RIDKIZ, JEMGIS, FOPDUL, NADQOZ, VOTMEY, LAQSIG, ONOQUE, BAXVEB, NAYNUX01, ULAVEK, PAKMAR, QARVOV, WAZDEG, CIKRET, HATYEG02, WIGKIH, IFUWIR, CAPKIP, KUXSEC, EXIHAV, COHWIF01, RIQWIZ, EXIGOI, ELIGOX, LIKLAU, WUHYIH, GIZYIY01, VOGWAQ, VEHCUG, APENOA, HECHOO, TMZBCO10, DIYHOJ, FIPZIN01, GAVKIX, KIZCUT, AZALIX, AZASER11, CECWAK, GOTSOX, TEMDOG, IJIVOM, HIZTEQ, PEFLUI, BIGFEC, TRZPUR, YUJDOW, LIWCUP, BALNIN04, UMOBAB, AFASOQ, XURCIW, HAZQUV, YOJQUL01, LUZFUJ, MOQLUA, ZZZDUI02, BEPNIT, GLICAC01, OCPNDN, LIPWEM, NIJHUJ, DUSHAB, ZZZKAY02, SIWDAD, ZBCNON01, HARJIV01, UREWAQ, QURYUZ01, OKINAY, WUMGAO, LONMEH, RESKIK, TIPHIL, TIPVIZ, YENDEC, GOGYUX, CIRXEH, TACGIN, DATFIO, HXANTH10, YINDUU, SEYJAH, QAKNIB, GAUTAM15, CUYXAV, URNFRO, HACMUT, XODBAU, AHIMOU, EFUMAU03, WAHBOX, SETFUS, AWIHOE02, NUPVUR, KEPNOI, XEFSIK, QODQEF, UTUVAI, HOJLIC, COXXUJ, UKIKEG, NBONAN01, ACOWOE, HOHTAZ02, GIRKOI, JAXSOR, OSOLEO, PAZRUF, SIBFOA, PHENOL11, MINPUR, TUNMUL, RONHEH, AJAPOR, EKUJIF, XOZHEZ, QEDFOW, IPIPAY, IYAWAG, MOZWII, VALIDL03, TABFAG, FOJMOG01, HTMTZC, FEYLUQ01, LESPUW, HEWFEW, VALWOW, LUHPAF, UFIYUD, XUHPIB, EMIJIT, FOYGIL, BUFKIX, ATOMAY, DAFLUO, REQQUA, AMTRAZ, NOKXEQ, QEDROG, QUICNA02, RATJOM, LEPMID, KONWOB, FUQYIB, YAMFOI, ULANUS, QELLOI, FEBKAZ, MUXGIX, SAWNIP, CAKREM, VOPJEP, JUMWUL, BCBANN02, PIKDAO, QEKQOM, CROTAC, SEYDIJ02, VOBJEB, BUTGAA, QONRAN, VIGLAA, HATWII, XARYIY, SITQET, MENPDL, KAJRIY, QOSRUN, MFCBXA, LETDUJ, PEPYIS, VUWXIU, IKALAJ01, MAFXAT, HAMHUZ, CXHIMZ20, KISJEC, WOQXOR, IMUWIX, PRUVAC, CEGXES, NOKYER, NAMVEE, EJIQEU, AMPYRZ, TAZPYR, TOLDAM01, LEZJAB, VAZZEB, QAFFIM, QOTWUR, GALDEC01, IWUGIQ01, TECKAO, ZOQJAQ, UNEWUG, ODEVUQ, VOHCEC, SUCANH16, IJARES, METRIM, LASCAC12, AROMAV, HOCWAX, ZEVJAL, TIPNEN, JONPUZ, KABWUF, DUFBOV11, YOBBEW, NAKVUT, CEDMUV, ACICAR, TIVSUM, DAZFLU11, FUTLEN, IHEPIU, HOYSOF, LSERIN12, YITKAP, YUFKUF, PUHJIM, NICGEL, FABFAQ, DAYQEZ, LUVNAS, REVMIP, QAGRIC, CADBOB, AMPHOM03, CABHUL, YUHTIE, GAGSIR01, IBUWAD, RIYZEF, YEKHIH, WOLNIW, MUYBAK, SAZFIJ, BODSAO, GLYCIN52, JIXHUU, GOCHIQ, YOVJUP01, XADBAI, BOVLEF, ELULOM, JOWWIB, LURYOM, FUGLEA, FUVDOP, MABZNA01, FETNUO, LODWOR, JEVPIJ, WIQDIJ, SOBBOA, SINMEI, FATZEH, VEFMOI, MILYIP, HMTOFA07, GIQRAZ, DMEBQU01, ULOBAA, USIZOM, HIVGAW, BACRAA, XIMQUG, POHHUQ, CAZDOY, NOGHIA, WASRAJ02, COFHOW, GULHEA, KEMHOB, SUCROS27, DILSEY, JOPNOR, BIDJON, GOMJUO, CEDXUE01, MCPRAC01, LIKKUN, RAMVUW, IMAZOL19, BEJTAL, GUDSUV, DMANAP02, SAFMAQ01, VEXGOU, MOGYOX, LIJJIY, IMICUE, SEGBUB, FOVYAQ01, ZAQJEI, KULGOO, TIHHOJ, FULYEQ10, IFIJOW, NTRTAC01, WIKGEC, MEPYRZ, VAZBIJ, SEZJEN, VANCOD, NIMVAG, XOHCEE, WIGBOE, KUDZAL, COWYET, QEYFUW, SARCEU, XIDQIL, TENZOD, GUZMUK, JICLAI, GIVXUE, WURLAY, PIFZAG, LIWJOQ, DUPDAU, HEPHUF, VITNIW, IVAGUH, PAGYUS, ANOLUL, RUGJUZ, LORJAD, TUGJUB, TARCOE, PHGLYA, KEPDIU, WOCVUF, JECVUI, OJUMOX, FEFNOT08, NNAPAN10, SOLHAD, GIZSOX, IHEHIO, IZARAC, NASQED01, IMEZIL, BIDRUB11, FEMFUY01, MODYOU, KEHYED, JERJOF, HUFFEU, JUXHUF, GENZEF, TBPMTZ, KOHPUT, AZOXCH, KEPFAO, LIJLEW, KOFKIZ, AMPYRE01, GEJCEF, LOBHIV, OKESAY, COYMUY, PNOIZA, HADFEY, ACACOX, PACFIL, THYMMH, EKATOB, CAXNIY, JEVPOQ, NABMIM, CAZBAI, ZAQHAC, HFULCA01, MOWBUX, SUPVOO, TOYLUO, ENINIA, HMBQOX10, GUSVOH, TEKQAB01, DOTTUB, HISYEO, WIFKEB01, PEDLER, DMAHOX01, SOQNIW, WUDHOU,

HXOXAM10, MEGLAN01, YOGJUB, DAFPUV, DIXRIL, SOJGED01, AKEZIB05, EDOREW, KOWRUJ, TUHHUZ, JUKWIV, LIVROY, YEHPIK, NOKLOP, KOBFUD, UNUWOQ, ASOTIL, TIGKUQ, PYMDON, MOHCUI, CEBGOF06, LEMSIF, ZOBYAQ, ALOPUR, QOLRIT, EVIXEM, CODPAN, LILLEY, ROFQIO, MALEHY11, YASSIV, JOHJIB01, MAZFOK, SASPAE, MCBPCX, ZEXJAP, MUKGAA, KEPFOC, APMCOX, XOZBUK01, ZELQOW, DHPROX, IMZMAL11, VIZWAE, OCEBAA, MOVNAN, FAJFII, GEZXAK, EROGOI, LADLOT, PICANO04, POXYAC, GUNSIS, CACGUI, YITJES, REYGUZ, FUQSAN, DEDNEF01, SOWSEC, BAKQUA, UHAMEV, SOJHEE, HEVLUP, YIRDAG, GOVKEH, EMOHAQ, CAZCAJ, YILNOY, HEYQUY, FIMQAU, TUPWEI, DEZBOB, IFIDUW, GAQKOA, YIHHON09, AHXGLP, YEKSOX, ENUNOR, FADCUI, REHCAK, IBPRAC14, WEJCEU, JABCAT, XIXQUR, AYUKIP, INOSND10, TIJLIH, JENHEP, ITIZUG, BIRGEO, LOHQAA, SEKPED, PITMAH, TAHHOC, IFOSON, FUSJOT, NAYKOO, JAYRAC, PYRCOX01, ZEQVIC, VEBTIG, QIJDES, LONRAI, APENUG, QUDPOW, FADTIQ, BENZAC18, HOQHUQ, PDABZA01, KEDBAX, FOXJIN, INEZOS, AGEQIO, AHPHAL01, ZILQOA01, OBAYOF, MOVLUG, EHIXOI, IHURUA, KEQQED, DOXKOR, GEJCIJ, CEBMUR, LIZCEC, XOSJUK, YIZXOV, QAPYUC, IKASUK, XUDTIB, APITEA, POVKAO, HEXTIN02, CEDHUQ, TADQAR, GEMZAZ, TOAZCD, IYAQOP01, LUYJIZ, DATGEL, XAGWIM02, RAWBAS06, FECVEP, TAZPYD10, DOZNOV, ACOGAA, UMEREJ, ACRLAC04, ZEGDIA, ODEZEE, DIHRUH10, HODMOD, QAMSII, EFAXIS, KAYSUY, ZILNOX01, HIXLEG, VAWMAJ, WADPEX, LSERIN21, NADQUF, TANPEE, TANCUI, BEXNIC, KEVXUG, BIZTOV, DACLOL, CEVTUU, KIXWIZ, RIZBOS, RATGEZ, OHOYUH, JUTGEK, HRFPZO10, ALATIN02, NUMBII, YAXHUC, ECADIV, QQQAUJ07, COWSOY, VUHDEJ, AXOSIQ, ERETAW, LISGUR, NIWCAY, DUVXIB, MNHCHA, ZIZRAD, NIPQUA, DOJNAS, XACZIN, ISEDOB, IPABUX, QIJDAO01, BAZYAC01, ACEQIK, QEJJUK, BENZIE01, HYXBUR10, MOAZCD, TEVJIP01, MEMALA, TORQIC, MUQXIH01, XADPEA, KADPOU02, BUWJEK, GOHHER, LEZQUE, VAZBUV, DIQCEN, NERJIE, GEDYAQ, QIWJEM, COJMUJ, HMNCXC01, PUSZOU, TALHET, RAGGOX, PAPHOD, WEHCAO, XEXYII, YANVUG, XAVPOZ01, PAPJUM, WUJQEY, ANGULF, KOJZUE, BOZGIH, NIRJEE04, BODFEF, DOJHOA, FEYTOU, FACQAE, KUSJIR, NOLDOJ01, XOMWAX, HEYJOK01, ALIWID, UPAWEP, UPOMIX, XABFOY, MIOZPO01, XOWNON, MMXPDZ10, OXOGEO, MEQPON, XULDUD01, JIXCOI01, UDAZIJ, PYRZAL10, KIKNEY, KAFLEI, FEZHAV, NINHYD02, DIMEDO03, YOLQOF, POVKIW, DIKXEB, AZETAC, DHANQU02, HAXTAD, AIPMGH, DOVDIC, HEKTEW, DOKQEA, BAGQIJ, GILCIP, HISTPU, MUDVUE, APITUQ, GUBZOS, XOYWEO01, LICROE, HUZLUJ, FURDCB01, KETVEK03, SUMKIU, MORPHN, LAQDUE, SIGFEU, XOKXIE, RAMGUH01, WIKRAL, AMOXAC, COHQEV, NEYKIN, SOWREB, QUSKOG, DZHPDO, PUVPED, ESAREV, HUNWUI, HOPKUT01, BOMHOA, NANQUO, XUDQUJ, MEXZOE, NADNIP, DASTAS, DIDFAY, DUBTEA, QIZBIL, NAPDCX, DMADEN10, GOGXUV, GADCIX, DIFPEO, MOJMEE, AMXBPM10, PYRIDO04, RAQVUB, NIYMAJ, KICCOO, SODBIW, VUXYEU, OCASIV, XAZDUY, GLUTAM08, XDHURC, MUKVUJ, IFAYIZ, KAMRAS, HMBFUR, DOZSUG, XOJQUK, EFUMAU02, YIKQOY, XEBHET, HOPLAA, TCMPCQ01, WIKCAU, IJUMEG, MEUREA01, WAKSUX, GAVLUK, LOKFIA, XOVWEK, ZERMOY, QEMHIA, ZZZNWQ01, ZAWDIM, MUVDOX, ZECWOV, HOLQUU, VEMFEY01, FUFMAU, XAJHEX, QANRUT01, GAVMUM, KAHHIM02, NABKIN, TAFKIU, PELDER, FIFBAY, YIFLOP, QIRRUG, BAWPOE, AMHPYR, UBEGUF, IHINEU, XAYROG, AZOBEN04, LOZJUF, DEXBEN, KUZKOG, CIGBAV, HEQWOR, RAYLAE, DOGQOF, UREJAD, GOLDIV, TEFMEW, LAYYOB, YUWGOO, ISIBER, ZEFPAD, SIXYEE, ULAVIO, WERHUX, GAFWIV, PEDERL, DIPGIS11, KECZAU, FEMMOA, CONXIN, CIRXAD, NUMRAP, DAHMII, TAHNEV, FOMNAX, VUBZUN, DEKRUG, RAYPOY, HOFGUF, QOWFUF, EVIQAB, QUCGEC, CBOHAZ04, TEDQAU, WAWVUN, NESFIA, SEGCOW, WIRYEB02, PHALIM, RUHGEI, ROFHAV, ZIYGUL, IKIDAH, XAJTIM, NSMACM, VACTIF, YENGOP, WAHGUI, NENFES, DOXDAN, CINWEC, GILZUW, MUXGOD, PORXUQ, SUCPYR11, UHAMOF, VEDLOG, UPOCEJ, WEHXOX, ZIWXEK, VIKNIM, DELXEX, KEKHEO01, CEYJUN, FINWOO, SAQZAK, ZACLEX, ZZZNYY01, QIFDOZ, NUVXIM, ANPRAL, ZEGBIY, EXETEI, PYRZOL05, QAMSUU, PUSYIM, YODSIT, VEBFIR, BEHZIX, PTZCOC, QEHBEL, DIKQUJ, SEDTUQ06, DEZBIV, MAYXEP, PORXEA, LIWCOJ, QAKFAK, ZERJIR, SOQNUI, WAKNUS, NOLCOG, BABXUA, KAQPUN, WAJPUV, SOVGIT, HIPXAF, DAFNON, GEDXIX, ILOFIA, VIGTUA, KECXIZ, FEHVIY, EMOGUJ, XUHREX, AMIPYZ, FIJKEP, YAMXUF, TABRET, ICRFRA10, WIQCUU, NEQWEN, KIFJAK, VUGMIV, ETANOL, IJEFAG, LEYXIW, JUMXEW, PEVKUX, QINFUO, SAYBID01, ULIZAS, OPISUC, BEVYOS, BZTZAD, BUYZAW, UGECOA, SABMAL, YEJYIV, PUYTEI, UDURUH, JASLEV, ZOQWOT, QALQIE, VIZZIP, NINDOD, BOGGAH, PEHXIK, ROFFEY, VALWIQ, HYZMAC, GLYALB02, DOXHPX, LEMGEQ, DUPNOT, OMEJOG, LAGVAT, ELUFOH, ORAVUZ, OQAHEV, VETQUI, AZIGAR, VUNKEU, EWUKIQ01, LEJYIJ, PUHHOQ, JOTKUY01, ANENUD01, DOHDEJ, CUNGAT, FERDUD, CASCIJ01, DIBGEA01, BANRUE, TIMBAU, KUYPAV02, DUDXIL, CEMGIK, EGAFAU, LAJGUA, FASFAG, KOVHAE01, BUKNEA, XISNET01, ZUHKIW, WAHNUO, MAQCEN, KERPII, MDXTCU, TIHQOR, YOWLUS, LIWGUU, BAZGOY05, OTOGEL, WAPPUY, NBZOAO05, DOJLAQ, DASRIY, WEJBIW, JOSXUK, ACYGLY12, SEWHUY, SUYQEK, ZUTKAC, CIDROW, VATJIK, CAZCIR, SORBPY20, DAZVEF, IGAMEJ, FIZJON01, FIKXAY, WIKCIE, MIRTOW, CATNUI, DOLTED, XARQAK, EYIJAY, HAXMAW01, LIWGOO, ABIVUD, UGUXAW, TAQXAL, JOQTAM, XOHXUP, FIGYID01, ACSALA10, GAQSEY, QAPZAJ, JUPROB, MIPHOL, BOXGEA, UNURUS, XISPOF, DOTRAG, FENTOH01, NICGAH, HOKSOR, SILDOH, MACLEI, VARGAX, AJASEK, EPAHOT, DAJXIV, HOFLAQ01, BUGDAI, PHTHAO, ROKBOI, URAWER, HAMJEL, MNIANL11, DISBUE, LANYAA, RAQRUX, SEGCAI01, CUTWIX, ELEYOJ, NITPOL02, YODHUV, UXAZEZ,

INEJOA, FOVVUI, LENYOS, SUYWOA, TODSAG, AMCYTS, PUNLOB, KUBPOM, AJODOU, PAGVAU, KENMIA, NUYJEW, OHEMAR, REDUCA, HOTNAF, EBULAO, VAYJIO, CAXKOB11, ROGDIB, ABUMAL, AJUKIZ01, OTAKEB, SIKGIC, QUHSUJ, EROWEO, MUZNEC, FUNXER, DNBENZ15, DUHYIP, GEYSEI, DNCOOC, WALDES, QOPYOK, TOXCTD, KOYLIT, FOXJOT, BUHGOA, EHUPAZ, ETBARB, PODFIZ, CMPDZB, EQIXEI, QQQFCY01, WECRED, VADQAU, YUYSIU, AJASAG, DMXBZQ01, HAVKOE, SEPNUX, YUKFUG, FUFSIK, LUZZEN, CIQSIE, JODPIC, BUDXII, WEMQUC, POLYUM, WUFVAW, GUDKAS, CUXXAW, XIVCUC, FEHCUQ, MAXYOZ, PEHXAC, MAZMEH, HIJFIP01, ROKTAO, TIMFUS, PPYRED, NOLDUP, PHTHIB, RODLOM, UNEVOZ01, AJAKOL, CBUDCX03, EACLID, CEKVIX, SOZHUM, QQQGEM01, XATZAT, BEXGAM, MIKTOQ, COVXAN01, YIHJOP, KIBDOO, PUTTUU, DIDJUX01, CROTAM, BUBNAN, CUBRUN, BAKTOX, VEZNEV, GORXOA, EDEBUK, YIFVAL, DORKEA, AMFURZ, ERIVAC, PAZDID, LOYXAA, FIGMAJ, ZEXYOQ, LUVPEY, HAJXIB, RAZXEV, WAWWAU, MAYCEW, ECUXUW, TAZWIB, FULTEM, XUBZID, PRONAC02, GOCJAK, ROFLAZ01, NOGUNA, CONNID, EQUXOD, LAFGIL, UKIJUV, TIWHAK, JAKKIP, VUXYAO, DUMPUW, UNEPOT, LOCVEE, XUMDUE, KUSLIU, GOMXIR, ADOJEK, PUMVOJ, LAPSIG, YITKIX, CYTOSM12, ACEMID03, SALMID05, CEDSAH, EVUMOX, SIGBOZ, PICNOE, YANSEN, NURWOM03, HMALAC01.

Quantum Espresso relaxed structures and corresponding chemical shielding are given as a separate xyz file in the Supplementary Dataset 1.

Supplementary Note 3: Environments Eliminated with the Unusual Environment Detection Procedure

^{1}H

Of the 76,214 ^{1}H environments of the CSD-2k, the following 211 environments were detected as unusual (the numbering follows the FPS order listed in Supplementary Note 2):
1536, 1537, 1538, 1539, 1792, 3295, 3296, 6807, 6808, 6809, 6810, 6831, 6832, 6833, 6834, 6843, 6844, 6845, 6846, 13876, 13877, 13884, 13885, 13896, 13897, 16544, 16545, 16546, 16547, 18285, 18286, 18765, 18766, 18767, 18768, 22192, 22193, 22194, 22195, 24448, 24449, 24450, 24451, 24464, 24465, 24466, 24467, 25466, 25467, 25468, 25469, 30674, 30675, 30676, 30677, 31670, 31671, 31672, 31673, 31674, 31675, 31676, 31677, 31678, 31679, 31680, 31681, 31682, 31683, 31684, 31685, 31686, 31687, 31688, 31689, 31690, 31691, 31692, 31693, 31694, 31695, 31696, 31697, 31698, 31699, 31700, 31701, 31702, 31703, 31790, 31791, 32742, 32743, 34296, 34297, 35394, 35395, 35810, 35811,35812, 35813, 35814, 35815, 35816, 35817, 36460, 36461, 36462, 36463, 36464, 36465, 36466, 36467, 36468, 36469, 36470, 36471, 36472, 36473, 36474, 36475, 37880, 37881, 37882, 37883, 37884, 37885, 37886, 37887, 38488, 38489, 38490, 38491, 38492, 38493, 38494, 38495, 42945, 42946, 43449, 43450, 43451, 43452, 44037, 44038, 44039, 44040, 44041, 44042, 44043, 44044, 44045, 44046, 44047, 44048, 44049, 44050, 44051, 44052, 49022, 49023, 49024, 49025, 49026, 49027, 49028, 49029, 52146, 52147, 52148, 52149, 54656, 54657, 54658, 54659, 58410, 58411, 62970, 62971, 62972, 62973, 63714, 63715, 66522, 66523, 66524, 66525, 68668, 68669, 68670, 68671, 68672, 68673, 68674, 68675, 68688, 68689, 68690, 68691, 70782, 70783, 70828, 70829, 72408, 72409, 72410, 72411, 74442, 74443, 74444, 74445.
These environments belong to the structures VIWYEH, QAHVUQ, ZACSOO, PORPIN01, VOFVAN26, CEVGIT, TIJKEC, PUPBAD02, TIMCHX, UPANIJ, MTETAC01, WUHYIH, HAZQUV, YOJQUL01, MOQLUA, CIRXEH, EKUJIF, QELLOI, SEYDIJ02, MAFXAT, NAKVUT, AMPHOM03, IMEZIL, AMPYRE01, JEVPOQ, OBAYOF, BAZYAC01, KAFLEI, QEMHIA, ZEGBIY, NOLCOG, CASCIJ01, ABIVUD, KENMIA, SEPNUX, FIGMAJ.

^{13}C

Of the 58,148 ^{13}C environments of the CSD-2k, the following 1,419 environments were detected as unusual (the numbering follows the FPS order listed in Supplementary Note 2):
96, 97, 470, 471, 472, 473, 676, 677, 764, 765, 770, 771, 918, 1027, 1028, 1029, 1030, 1311, 1312, 1367, 1468, 1469, 1470, 1471, 1484, 1485, 1486, 1487, 1500, 1501, 1502, 1503, 1516, 1517, 1518, 1519, 1766, 1796, 1882, 1883, 1884, 1885, 1886, 1887, 1888, 1889, 1894, 1895, 1896, 1897, 1898, 1899, 1900, 1901, 1902, 1903, 1904, 1905, 1906, 1907, 1908, 1909, 1914, 1915, 1916, 1917, 1918, 1919, 1920, 1921, 2148, 2149, 2150, 2151, 2156, 2157, 2158, 2159, 2164, 2165, 2166, 2167, 2172, 2173, 2174, 2175, 2180, 2181, 2182, 2183, 2264, 2265, 2270, 2271, 2352, 2353, 2354, 2355, 2754, 2755, 2756, 2757, 2790, 2791, 2792, 2793, 2832, 2833, 3038, 3039, 3040, 3041, 3514, 3515, 3516, 3517, 3780, 3781, 3942, 3943, 3944, 3945, 3946, 3947, 3948, 3949, 3990, 3991, 3992, 3993, 3994, 3995, 3996, 3997, 4006, 4007, 4008, 4009, 4010, 4011, 4012, 4013, 4108, 4109, 4110, 4111, 4112, 4113, 4114, 4115, 4132, 4133, 4134, 4135, 4136, 4137, 4138, 4139, 4298, 4299, 4300, 4301, 4314, 4315, 4316, 4317, 4394, 4395, 4396, 4397, 5034, 5035, 5036, 5037, 5618, 5619, 5620, 5621, 5785, 5786, 5805, 5806, 5809, 5810, 5821, 5822, 5860, 5861, 5862, 5863, 6606, 6607, 6656, 6657, 6658, 6659, 6672, 6673, 6674, 6675, 6684, 6685, 6686, 6687, 6688, 6689, 6690,

6691, 6692, 6693, 6694, 6695, 6700, 6701, 6702, 6703, 6716, 6717, 6718, 6719, 6728, 6729, 6730, 6731, 6732, 6733, 6734, 6735, 6792, 6793, 6794, 6795, 6828, 6829, 6830, 6831, 6852, 6853, 6854, 6855, 6856, 6857, 6858, 6859, 7086, 7087, 7088, 7089, 7090, 7091, 7092, 7093, 7376, 7377, 7378, 7379, 7388, 7389, 7390, 7391, 7456, 7457, 7458, 7459, 7460, 7461, 7462, 7463, 7567, 7568, 7569, 7570, 7571, 7572, 7591, 7592, 7593, 7594, 7595, 7596, 7597, 7598, 7599, 7600, 7601, 7602, 7639, 7640, 7641, 7642, 7643, 7644, 7645, 7646, 7981, 7982, 7983, 7984, 7985, 7986, 7987, 7988, 8175, 8176, 8179, 8180, 8187, 8188, 8341, 8342, 8343, 8344, 8345, 8346, 8347, 8348, 8465, 8466, 8467, 8468, 8469, 8470, 8471, 8472, 8591, 8592, 8593, 8594, 8599, 8600, 8601, 8602, 8847, 8848, 8849, 8850, 9099, 9100, 9101, 9102, 9103, 9104, 9105, 9106, 9107, 9108, 9109, 9110, 9171, 9172, 9173, 9174, 9187, 9188, 9189, 9190, 9191, 9192, 9193, 9194, 9307, 9308, 9309, 9310, 9315, 9316, 9317, 9318, 9463, 9464, 9465, 9466, 9591, 9592, 9927, 9928, 10143, 10144, 10147, 10148, 10353, 10354, 10367, 10368, 10371, 10372, 10849, 10850, 10851, 10852, 11457, 11458, 11571, 11572, 11573, 11574, 11961, 11962, 12407, 12408, 12721, 12722, 12723, 12724, 12725, 12726, 12727, 12728, 12845, 12846, 12849, 12850, 12883, 12884, 12885, 12886, 12969, 12970, 12971, 12972, 13319, 13320, 13329, 13330, 13331, 13332, 13349, 13350, 13351, 13352, 13485, 13486, 13487, 13488, 13727, 13728, 13729, 13730, 13907, 13908, 13909, 13910, 13911, 13912, 13913, 13914, 13919, 13920, 13921, 13922, 14093, 14094, 14247, 14248, 14249, 14250, 14829, 14830, 14839, 14840, 14979, 14980, 14981, 14982, 15525, 15526, 15527, 15528, 15537, 15538, 15539, 15540, 15767, 15768, 15769, 15770, 15771, 15772, 15773, 15774, 16275, 16276, 16277, 16278, 16355, 16356, 16357, 16358, 16359, 16360, 16361, 16362, 16371, 16372, 16373, 16374, 16411, 16412, 16413, 16414, 16497, 16498, 16499, 16500, 16501, 16502, 16503, 16504, 16565, 16566, 16567, 16568, 16569, 16570, 16571, 16572, 16573, 16574, 16575, 16576, 16577, 16578, 16579, 16580, 16581, 16582, 16583, 16584, 16585, 16586, 16587, 16588, 16945, 16946, 16947, 16948, 16949, 16950, 16951, 16952, 16953, 16954, 16955, 16956, 16957, 16958, 16959, 16960, 17167, 17168, 17181, 17182, 17461, 17462, 18805, 18806, 18807, 18808, 19363, 19364, 19365, 19366, 19539, 19540, 19541, 19542, 20143, 20144, 20145, 20146, 20159, 20160, 20161, 20162, 20857, 20858, 20859, 20860, 20929, 20930, 21279, 21280, 21281, 21282, 21671, 21672, 21673, 21674, 21675, 21676, 22085, 22086, 22087, 22088, 22119, 22120, 22121, 22122, 22123, 22124, 22125, 22126, 22277, 22278, 22279, 22280, 22305, 22306, 22307, 22308, 22317, 22318, 22319, 22320, 22597, 22598, 22599, 22600, 22701, 22702, 22703, 22704, 22765, 22766, 22767, 22768, 22815, 22816, 22817, 22818, 22819, 22820, 22821, 22822, 22927, 22928, 22929, 22930, 22947, 22948, 22957, 22958, 23005, 23006, 23325, 23326, 23327, 23328, 23329, 23330, 23331, 23332, 23333, 23334, 23335, 23336, 23337, 23338, 23339, 23340, 23341, 23342, 23343, 23344, 23345, 23346, 23347, 23348, 23427, 23428, 23877, 23878, 23879, 23880, 24281, 24282, 24283, 24284, 24301, 24302, 24303, 24304, 24305, 24306, 24307, 24308, 24309, 24310, 24311, 24312, 24329, 24330, 24331, 24332, 24333, 24334, 24335, 24336, 24523, 24524, 24787, 24788, 25371, 25372, 25601, 25602, 25603, 25604, 25605, 25606, 25759, 25760, 25761, 25762, 25763, 25764, 25765, 25766, 26093, 26094, 26095, 26096, 26295, 26296, 26299, 26300, 26303, 26304, 26309, 26310, 26313, 26314, 26317, 26318, 26347, 26348, 26349, 26350, 26449, 26450, 26451, 26452, 26561, 26562, 26563, 26564, 26715, 26716, 26803, 26804, 26805, 26806, 27015, 27016, 27017, 27018, 27019, 27020, 27021, 27022, 27039, 27040, 27041, 27042, 27043, 27044, 27045, 27046, 27425, 27426, 27427, 27428, 27935, 27936, 27965, 27966, 27967, 27968, 27997, 27998, 27999, 28000, 28001, 28002, 28003, 28004, 28123, 28124, 28129, 28130, 28209, 28210, 28211, 28212, 28229, 28230, 28231, 28232, 28429, 28430, 28455, 28456, 28508, 28553, 28554, 28555, 28556, 28843, 28844, 28845, 28846, 29313, 29314, 29417, 29418, 29419, 29420, 29425, 29426, 29427, 29428, 29433, 29434, 29435, 29436, 29553, 29554, 29555, 29556, 29573, 29574, 29575, 29576, 29627, 29628, 29629, 29630, 29763, 29764, 29765, 29766, 29775, 29776, 29777, 29778, 29857, 29858, 30257, 30258, 30259, 30260, 30357, 30358, 30359, 30360, 30989, 30990, 30991, 30992, 31249, 31250, 31251, 31252, 31273, 31274, 31275, 31276, 31445, 31446, 31447, 31448, 31489, 31490, 31963, 31964, 31965, 31966, 32083, 32084, 32085, 32086, 32179, 32180, 32181, 32182, 32183, 32184, 32189, 32190, 32381, 32382, 32383, 32384, 32449, 32450, 32451, 32452, 32457, 32458, 32459, 32460, 32725, 32726, 32727, 32728, 32965, 32966, 32967, 32968, 33001, 33002, 33003, 33004, 33009, 33010, 33011, 33012, 33021, 33022, 33023, 33024, 33033, 33034, 33035, 33036, 33093, 33094, 33095, 33096, 33171, 33279, 33280, 33345, 33346, 33843, 33844, 33845, 33846, 33871, 33872, 33873, 33874, 33875, 33876, 33877, 33878, 33887, 33888, 33889, 33890, 33891, 33892, 33893, 33894, 34357, 34358, 34359, 34360, 34761, 34762, 34763, 34764, 35193, 35194, 35195, 35196, 35201, 35202, 35203, 35204, 35809, 35810, 35811, 35812, 35899, 35900, 35901, 35902, 36051, 36052, 36593, 36594, 36595, 36596, 36609, 36610, 36611, 36612, 37081, 37082, 37083, 37084, 37275, 37276, 37277, 37278, 37279, 37280, 37281, 37282, 37747, 37748, 37749, 37750, 37751, 37752, 37753, 37754, 37763, 37764, 37765, 37766, 37767, 37768, 37769, 37770, 37799, 37800, 37801, 37802, 37955, 37956, 37957, 37958, 38049, 38050, 38927, 38928, 38929, 38930, 39241, 39242, 39243, 39244, 39861, 39862, 39863, 39864, 40495, 40496, 40497, 40498, 40827, 40828, 40829, 40830, 41715, 41716, 41717, 41718, 41859, 41860, 41861, 41862, 42308, 42309, 42310, 42311, 42312, 42313, 42314, 42315, 42780, 42781, 42782, 42783, 42808, 42809, 42810, 42811, 42816, 42817, 42818, 42819, 43132, 43133, 43134, 43135, 43808, 43809, 43810, 43811, 43820, 43821, 43822, 43823, 44436, 44437, 44438, 44439, 44784, 44785, 44786, 44787, 44792, 44793, 44794, 44795, 44916, 44917, 44918, 44919, 44928, 44929, 44930, 44931, 45330, 45331, 45436, 45437, 45438, 45439, 45622, 45623, 45628, 45629, 45896, 45897, 45898,

45899, 45904, 45905, 45906, 45907, 46102, 46103, 46104, 46105, 46106, 46107, 46108, 46109, 46650, 46651, 46652, 46653, 46666, 46667, 46668, 46669, 46690, 46691, 46692, 46693, 46698, 46699, 46700, 46701, 46758, 46759, 46760, 46761, 47110, 47111, 47112, 47113, 47234, 47235, 47236, 47237, 47312, 47313, 47314, 47315, 47380, 47381, 47396, 47397, 47618, 47619, 47622, 47623, 47624, 47625, 47628, 47629, 47786, 47787, 47788, 47789, 47802, 47803, 47804, 47805, 48014, 48015, 48210, 48211, 48212, 48213, 48374, 48375, 48376, 48377, 48414, 48415, 48416, 48417, 48484, 48485, 48492, 48493, 48652, 48653, 48654, 48655, 48656, 48657, 48658, 48659, 48758, 48759, 48760, 48761, 49168, 49169, 49170, 49171, 49172, 49173, 49174, 49175, 49622, 49623, 49624, 49625, 49662, 49663, 49664, 49665, 50186, 50187, 50188, 50189, 50190, 50191, 50192, 50193, 50386, 50387, 50388, 50389, 50514, 50515, 50516, 50517, 50554, 50555, 50556, 50557, 50874, 50875, 50876, 50877, 50878, 50879, 50896, 50897, 50904, 50905, 51188, 51189, 51190, 51191, 51220, 51221, 51222, 51223, 51314, 51315, 51316, 51317, 51342, 51343, 51344, 51345, 51438, 51439, 51496, 51497, 51574, 51575, 51576, 51577, 51586, 51587, 51588, 51589, 51590, 51591, 51592, 51593, 51606, 51607, 51608, 51609, 51618, 51619, 51620, 51621, 51634, 51635, 52016, 52017, 52212, 52213, 52278, 52279, 52280, 52281, 52286, 52287, 52288, 52289, 52472, 52473, 52474, 52475, 52788, 52789, 52790, 52791, 52792, 52793, 52794, 52795, 52808, 52809, 52810, 52811, 52828, 52829, 52830, 52831, 52844, 52845, 52846, 52847, 53180, 53181, 53182, 53183, 53200, 53204, 53205, 53206, 53207, 53696, 53697, 53700, 53701, 53702, 53703, 54376, 54377, 54378, 54379, 54388, 54389, 54390, 54391, 54684, 54685, 54686, 54687, 54848, 54849, 55330, 55331, 55368, 55369, 55406, 55407, 55408, 55409, 55410, 55411, 55412, 55413, 55750, 55751, 55832, 55833, 55834, 55835, 55876, 55877, 56024, 56025, 56026, 56027, 56028, 56029, 56030, 56031, 56420, 56421, 56422, 56423, 56824, 56825, 56826, 56827, 57080, 57081, 57082, 57083, 57380, 57381, 57382, 57383, 57440, 57441, 57442, 57443, 57540, 57541, 57542, 57543.
These environments belong to the structures AMFORM, FOKSUT, JOJWOU03, LEPFES, CUZJEO, CALFOM, DIOXAZ10, QAHVUQ, GANHUY, MEVDAT, EMUFAU, UMERAF, MUYHIY, QOXPEZ, GEFBAV, CITSED10, FESNEW15, EJEHEG, JAPBIL, OXHEPA, GACGAU, VOQXAA, TETZIB, IVEZEO, RIKPEG, GAQWAX, DNBOIM, QODBOC, SAGLAQ, HONSIO, GEJSET, PRMDIN20, AXOSOW, DZBASK, AMTETA, FAFKEC, CYAMPD01, OMIWEM, ITIPEG, KABBEW, XZBTZO, NEPBEQ, NBFURX, PUFFOL01, ZUNTAD, NIBZAM, LIVZIA, XIHLIL, NEBPUF, HEHQOC, VOFVAN26, DOZXEW, POFLAX01, LEGLEQ, KAMFAH, LESLAX, BZCBUO, ZOVHID, KOVGIL, KIXXEW, OGOKEC, FEHDAX01, GEXMED, FOLVIL, PUPBAD02, LUNYID, DALGON03, HABFUL, PMXCDO10, HUKROU, BECYAL, WOFVAQ, VANCIX, XUDSOG, XACSOL, BUVBUR, TIMCHX, JUVKAM, DODCHD, YITBOS, MTETAC01, QOXMUM, DOYTUG, WOKPAO, CORYIR10, TAXTER, QAHCUA, TABBEE, NADQOZ, BAXVEB, CIKRET, WUHYIH, VEHCUG, HECHOO, AZALIX, AZASER11, TEMDOG, HAZQUV, MOQLUA, WUMGAO, CUYXAV, WAHBOX, QODQEF, QEDFOW, FOJMOG01, HEWFEW, QUICNA02, CAKREM, JUMWUL, QEKQOM, VOBJEB, XARYIY, MENPDL, MAFXAT, LEZJAB, LASCAC12, AROMAV, HOCWAX, KABWUF, YOBBEW, DAZFLU11, IHEPIU, YITKAP, CABHUL, ELULOM, FUVDOP, LODWOR, JEVPIJ, WIQDIJ, MILYIP, DMEBQU01, DILSEY, CEDXUE01, VANCOD, GUZMUK, PIFZAG, DUPDAU, SOLHAD, IZARAC, FEMFUY01, GENZEF, KOHPUT, PNOIZA, CAXNIY, JEVPOQ, ZAQHAC, MOWBUX, HMBQOX10, DOTTUB, JUKWIV, LIVROY, ZOBYAQ, MUKGAA, LADLOT, YEKSOX, FADCUI, AYUKIP, LONRAI, IHURUA, QAPYUC, UMEREJ, ACRLAC04, EFAXIS, HIXLEG, VUHDEJ, BAZYAC01, DIQCEN, NIRJEE04, HEYJOK01, BAGQIJ, GUBZOS, SOWREB, DASTAS, MOJMEE, TCMPCQ01, WIKCAU, PELDER, LOZJUF, CIGBAV, ULAVIO, GAFWIV, CONXIN, RAYPOY, TEDQAU, DOXDAN, GILZUW, DELXEX, CEYJUN, ZACLEX, QIFDOZ, PUSYIM, PTZCOC, LIWCOJ, SOVGIT, VIGTUA, FEHVIY, YAMXUF, WIQCUU, OPISUC, NINDOD, BOGGAH, PUHHOQ, TIMBAU, CEMGIK, EGAFAU, WAHNUO, WAPPUY, WEJBIW, SEWHUY, CIDROW, DAZVEF, IGAMEJ, HAXMAW01, XOHXUP, GAQSEY, DOTRAG, DAJXIV, RAQRUX, KENMIA, DNCOOC, PODFIZ, WECRED, POLYUM, GUDKAS, HIJFIP01, PPYRED, PHTHIB, CBUDCX03, BUBNAN, LOYXAA, TAZWIB, TIWHAK, VUXYAO, UNEPOT.

^{15}N

Of the 27,814 ^{13}C environments of the CSD-2k, the following 514 environments were detected as unusual (the numbering follows the FPS order listed in Supplementary Note 2):
20, 21, 26, 27, 32, 33, 1255, 1256, 1257, 1258, 1259, 1260, 1261, 1262, 1263, 1264, 1265, 1266, 1267, 1268, 1269, 1270, 1551, 1552, 1553, 1554, 1555, 1556, 1557, 1558, 1655, 1656, 1673, 1674, 1745, 1746, 1823, 1824, 1833, 1834, 1959, 1960, 1961, 1962, 2535, 2536, 2537, 2538, 2969, 2970, 2971, 2972, 2973, 2974, 2975, 2976, 3013, 3014, 3145, 3146, 3147, 3148, 3189, 3190, 3191, 3192, 3305, 3306, 3307, 3308, 3317, 3318, 3319, 3320, 3354, 3355, 3871, 3872, 3873, 3874, 4137, 4138, 4139, 4140, 4141, 4142, 4143, 4144, 4188, 4189, 4190, 4191, 4192, 4193, 4200, 4201, 4202, 4203, 4204, 4205, 4206, 4207, 4208, 4209, 4210, 4211, 4212, 4213, 4214, 4215, 4252, 4253, 4254, 4255, 4260, 4261, 4262, 4263, 4388, 4389, 4390, 4391, 4468, 4469, 4470, 4471, 4472, 4473, 4474, 4475, 4936, 4937, 4938, 4939, 4940, 4941, 4942, 4943, 5090, 5091, 5100, 5101, 5282, 5283, 5284, 5285, 5286, 5287, 5288, 5289, 5958, 5959, 5960, 5961, 5962, 5963, 5964, 5965, 5998, 5999, 6000, 6001, 6050, 6051, 6052, 6053, 6054, 6055, 6056, 6057, 6400, 6401, 6402, 6403, 6412, 6413, 6414, 6415, 6618, 6619, 6620, 6621, 7252, 7253, 7254, 7255, 7256, 7257, 8003, 8004, 8005, 8006, 8171, 8172, 8173, 8174, 8564, 8565, 8566, 8567, 8968, 8969, 8970, 8971, 9186, 9187,

9188, 9189, 9190, 9191, 9192, 9193, 9478, 9479, 9480, 9481, 9482, 9483, 9484, 9485, 9526, 9527, 9528, 9529, 9530, 9531, 9532, 9533, 9694, 9695, 9696, 9697, 9974, 9975, 10446, 10447, 10448, 10449, 10450, 10451, 10452, 10453, 11132, 11133, 11134, 11135, 11140, 11141, 11142, 11143, 12286, 12287, 12288, 12289, 12468, 12469, 12470, 12471, 12472, 12473, 12474, 12475, 13188, 13189, 13190, 13191, 13290, 13291, 13462, 13463, 13464, 13465, 13466, 13467, 13468, 13469, 13496, 13497, 13498, 13499, 13802, 13803, 13804, 13805, 13806, 13807, 13808, 13809, 14520, 14521, 14522, 14523, 14524, 14525, 14526, 14527, 14752, 14753, 14754, 14755, 14756, 14757, 15002, 15003, 15060, 15061, 15062, 15063, 15216, 15217, 15218, 15219, 15512, 15513, 15514, 15515, 15663, 15667, 15671, 15675, 15708, 15709, 15710, 15711, 16179, 16180, 16181, 16182, 16183, 16184, 16185, 16186, 16239, 16240, 16593, 16594, 16595, 16596, 16905, 16906, 16907, 16908, 16915, 16916, 17391, 17392, 17393, 17394, 17399, 17400, 17401, 17402, 17403, 17404, 17405, 17406, 18916, 18917, 19246, 19247, 19248, 19249, 19254, 19255, 19256, 19257, 19342, 19343, 19592, 19593, 19616, 19617, 19832, 19833, 19834, 19835, 20320, 20321, 20322, 20323, 20324, 20325, 20326, 20327, 20328, 20329, 20330, 20331, 20332, 20333, 20334, 20335, 20436, 20437, 20438, 20439, 20440, 20441, 20442, 20443, 20622, 20623, 20624, 20625, 20900, 20901, 20902, 20903, 21580, 21581, 21896, 21897, 21898, 21899, 22094, 22095, 22096, 22097, 22194, 22195, 22412, 22413, 22414, 22415, 23012, 23013, 23014, 23015, 23448, 23449, 23450, 23451, 23464, 23465, 23466, 23467, 23654, 23655, 23656, 23657, 23874, 23875, 23876, 23877, 23878, 23879, 23880, 23881, 24086, 24087, 24088, 24089, 24090, 24091, 24092, 24093, 24170, 24171, 24172, 24173, 24174, 24175, 24176, 24177, 24246, 24247, 24504, 24505, 24506, 24507, 24524, 24525, 24526, 24527, 24858, 24859, 24860, 24861, 25484, 25485, 25486, 25487, 25504, 25505, 25506, 25507, 25508, 25509, 25510, 25511, 25512, 25513, 25514, 25515, 25516, 25517, 25518, 25519, 25596, 25597, 25598, 25599, 25970, 25971, 26396, 26397, 26398, 26399, 27302, 27303, 27400, 27401, 27402, 27403.
These environments belong to the structures COVSEM, UMERAF, DAJVUG01, UJEREG01, ZAXLIV, CITSED10, TIGBIU, SAFKAL, TETZIB, JACPOS01, ESIWUZ, APENIU, FORRUB, DNBOIM, PRMDIN20, DZBASK, FAFKEC, CYAMPD01, YOVVIP, XOCZUL, OMIWEM, XZBTZO, IXAQAB, NOPPOY, HEHQOC, MOPPUC01, SAPDAQ, LUSNOC, PEKSAZ, BUFMOG, AYAHUE, FIMWII, TOCLUU, JEMFUD, OXAYAO, XENQUD, TIMCHX, XIMKUA, BIZBET, BUSFUQ, QUDNAG, KIWJIK, APENOA, AZASER11, HAZQUV, LUZFUJ, CUYXAV, XUHPIB, CAKREM, VUWXIU, HAMHUZ, NAMVEE, AROMAV, IHEPIU, YITKAP, FUVDOP, LODWOR, BIDJON, VANCOD, NIMVAG, IHEHIO, HEVLUP, REHCAK, AYUKIP, APENUG, DOXKOR, UMEREJ, TANPEE, YAXHUC, BAZYAC01, UDAZIJ, GUBZOS, ESAREV, QIZBIL, IFAYIZ, LAYYOB, NSMACM, VACTIF, DELXEX, PTZCOC, WAKNUS, HIPXAF, FEHVIY, BUYZAW, PUHHOQ, HAXMAW01, ABIVUD, GAQSEY, PUNLOB, PODFIZ, XUBZID, NOGUNA.

^{17}O

Of the 25,924 ^{13}C environments of the CSD-2k, the following 441 environments were detected as unusual (the numbering follows the FPS order listed in Supplementary Note 2):
232, 233, 234, 235, 308, 309, 310, 311, 312, 313, 314, 315, 378, 379, 380, 381, 1390, 1391, 1392, 1393, 1442, 1443, 1550, 1551, 1552, 1553, 1910, 1911, 1912, 1913, 1914, 1915, 1916, 1917, 2058, 2059, 2060, 2061, 2066, 2067, 2068, 2069, 2320, 2321, 2322, 2323, 2540, 2541, 2542, 2543, 2760, 2761, 2762, 2763, 3048, 3049, 3300, 3301, 3440, 3441, 3442, 3443, 3908, 3909, 3910, 3911, 3912, 3913, 3914, 3915, 3968, 3969, 3970, 3971, 4036, 4037, 4042, 4043, 4264, 4268, 4270, 4272, 4276, 4278, 4592, 4593, 4594, 4595, 4596, 4597, 4598, 4599, 4600, 4601, 4602, 4603, 4798, 4799, 4800, 4801, 4802, 4803, 4804, 4805, 5244, 5245, 5246, 5247, 5544, 5545, 5546, 5547, 5856, 5857, 5858, 5859, 6886, 6887, 6888, 6889, 7206, 7207, 7208, 7209, 7350, 7351, 7352, 7353, 7478, 7479, 7480, 7481, 7660, 7661, 7662, 7663, 7762, 7763, 7764, 7765, 8028, 8348, 8349, 8350, 8351, 8352, 8353, 8354, 8355, 8426, 8427, 8748, 8749, 8750, 8751, 8752, 8753, 8754, 8755, 9636, 9637, 9660, 9661, 9662, 9663, 10006, 10007, 10158, 10159, 10160, 10161, 10162, 10163, 10164, 10165, 10288, 10289, 10290, 10291, 10292, 10293, 10294, 10295, 10324, 10325, 10326, 10327, 10712, 10713, 11158, 11159, 11160, 11161, 11558, 11559, 11560, 11561, 11562, 11563, 11564, 11565, 11590, 11591, 11592, 11593, 11594, 11595, 11596, 11597, 11598, 11599, 11600, 11601, 12656, 12657, 12658, 12659, 12802, 12803, 12804, 12805, 12806, 12807, 12808, 12809, 12810, 12811, 12812, 12813, 12814, 12815, 12816, 12817, 12946, 12947, 12948, 12949, 12960, 12961, 12962, 12963, 12964, 12965, 12966, 12967, 13108, 13109, 13110, 13111, 13140, 13141, 13142, 13143, 13160, 13161, 13162, 13163, 13164, 13165, 13166, 13167, 13168, 13169, 13170, 13171, 13438, 13439, 13444, 13445, 13450, 13451, 13452, 13453, 13458, 13459, 13460, 13461, 13502, 13503, 13504, 13505, 13628, 13629, 13630, 13631, 13709, 13712, 13715, 13718, 13765, 13766, 13767, 13768, 14839, 14840, 14841, 14842, 14991, 14992, 14993, 14994, 15325, 15326, 15327, 15328, 15553, 15554, 15555, 15556, 15609, 15610, 15611, 15612, 15891, 15892, 15893, 15894, 15915, 15916, 15917, 15918, 15919, 15920, 15921, 15922, 15935, 15936, 16011, 16012, 16013, 16014, 16015, 16016, 16017, 16018, 16685, 16686, 17157, 17158, 17159, 17160, 17219, 17220, 17411, 17412, 17413, 17414, 17437, 17438, 18213, 18214, 18215, 18216, 18587, 18588, 18589, 18590, 18615, 18616, 18617, 18618, 18619, 18620, 18621, 18622, 18725, 18726, 18727, 18728, 19111, 19112, 19113, 19114, 19115, 19116, 19117, 19118, 20180, 20181, 20182, 20183, 20494, 20495, 20496, 20497, 20708, 20709, 21810, 21811, 21812, 21813, 23088, 23089, 23090, 23091, 23108, 23109, 23110, 23111, 23412, 23413, 23414, 23415, 23532, 23533, 23534, 23535, 23688, 23689, 23690,

23691, 23694, 23695, 24258, 24259, 24260, 24261, 24552, 24553, 24554, 24555, 24556, 24557, 24558, 24559, 24792, 24793, 24794, 24795, 24796, 24797, 24798, 24799, 25144, 25145, 25430, 25431, 25556, 25557, 25558, 25559.

These environments belong to the structures PARBAC03, BCBANN06, NUQPOF, TEDROL, CITSED10, FESNEW15, NIKVUZ, MEOXAL, BONGIW, OFUKOR, RIKPEG, PARGIZ, ZERZIH, HONSIO, UDOZUJ, OMIWEM, EKUKEC, ZAVTEV, PUFFOL01, UTAKUX, JOPNAD, PEKSAZ, GADZER, ZEXKEU, NUPTIC, PEJBAJ, TRIRED, BELBUP02, OXAYAO, CANXIA, JUVKAM, XIMKUA, NILQAC, DUSBUQ, DOYTUG, YEKJEF, PUQJOC, CORYIR10, QAFFOS, TABBEE, IFUWIR, LIWCUP, HAZQUV, HEWFEW, QUICNA02, CAKREM, BCBANN02, KAJRIY, MFCBXA, LETDUJ, PEPYIS, TECKAO, ZOQJAQ, IJARES, KABWUF, IHEPIU, YITKAP, VANCOD, LIWJOQ, IHEHIO, KOHPUT, GEJCEF, HMBQOX10, TEKQAB01, DOTTUB, HXOXAM10, POXYAC, FADCUI, AYUKIP, LONRAI, QUDPOW, TANPEE, DOJNAS, ISEDOB, XADPEA, NIRJEE04, IFAYIZ, HOLQUU, FIFBAY, FINWOO, TIMBAU, JOSXUK, FIZJON01, HAXMAW01, KUBPOM, DUHYIP, DMXBZQ01, CEKVIX, RAZXEV, NOGUNA.

Supplementary Note 4: Structures and Chemical Shifts of the CSD-6 Set

For all of the structures in CSD-6 we removed atoms with partial occupations, following the same procedure as for the CSD-61k set, leaving only one conformation in the structure file. Missing Hydrogen atoms were added with the program IQmol. Prior to the chemical shift calculations all the coordinates of the structures were DFT optimized using the same parameters as for the CSD-2k set. Chemical shieldings predicted for each structures are given as separate .xyz files in the Supplementary Dataset 9, named according to the corresponding CSD Refcodes. For each atom we report: atom type, Cartesian coordinates and predicted isotropic chemical shielding.

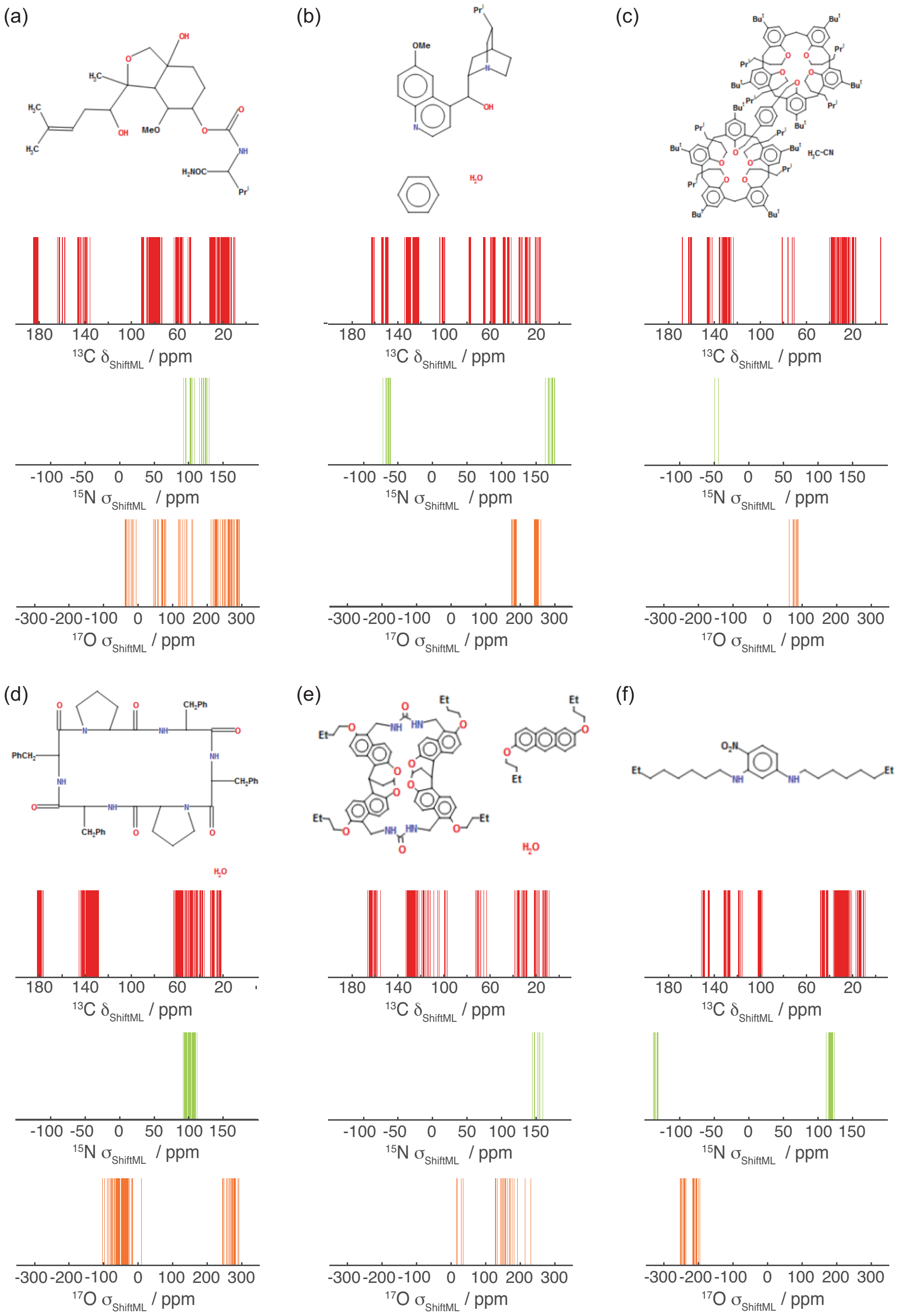

Supplementary Figure 10. Chemical formula and corresponding ^{13}C, ^{15}N and ^{17}O NMR spectra predicted using ShiftML of the six large molecular crystals with CSD Refcodes. (a) CAJVUH,[26] N_{atoms} = 828, (b) RUKTOI,[27] N_{atoms} = 768, (c) EMEMUE,[28] N_{atoms} = 860, (d) GOKXOV,[29] N_{atoms} = 945, (e) HEJBUW,[30] N_{atoms} = 816, (f) RAYFEF,[31] N_{atoms} = 1,584.

Supplementary References

1 Groom, C. R., Bruno, I. J., Lightfoot, M. P. & Ward, S. C. The Cambridge structural database. *Acta Crystallogr., Sect. B: Struct. Sci., Cryst. Eng. Mater.* **72**, 171-179, doi:10.1107/S2052520616003954 (2016).
2 Day, G. M., Motherwell, W. S. & Jones, W. A strategy for predicting the crystal structures of flexible molecules: the polymorphism of phenobarbital. *Phys. Chem. Chem. Phys.* **9**, 1693-1704, doi:10.1039/b612190j (2007).
3 Baias, M. *et al.* Powder crystallography of pharmaceutical materials by combined crystal structure prediction and solid-state 1H NMR spectroscopy. *Phys. Chem. Chem. Phys.* **15**, 8069-8069, doi:10.1039/c3cp41095a (2013).
4 Baias, M. *et al.* De novo determination of the crystal structure of a large drug molecule by crystal structure prediction-based powder NMR crystallography. *J. Am. Chem. Soc.* **135**, 17501-17507, doi:10.1021/ja4088874 (2013).
5 Pickard, C. J. & Mauri, F. All-electron magnetic response with pseudopotentials: NMR chemical shifts. *Phys. Rev. B* **63**, doi:ARTN 245101 DOI 10.1103/PhysRevB.63.245101 (2001).
6 Yates, J. R., Pickard, C. J. & Mauri, F. Calculation of NMR chemical shifts for extended systems using ultrasoft pseudopotentials. *Phys. Rev. B* **76**, 024401, doi:ARTN 024401 10.1103/PhysRevB.76.024401 (2007).
7 Clark, S. J. *et al.* First principles methods using CASTEP. *Zeitschrift Fur Kristallographie* **220**, 567-570, doi:DOI 10.1524/zkri.220.5.567.65075 (2005).
8 Giannozzi, P. *et al.* QUANTUM ESPRESSO: a modular and open-source software project for quantum simulations of materials. *J. Phys.: Condens. Matter* **21**, 395502, doi:10.1088/0953-8984/21/39/395502 (2009).
9 Giannozzi, P. *et al.* Advanced capabilities for materials modelling with Quantum ESPRESSO. *J. Phys.: Condens. Matter* **29**, 465901, doi:10.1088/1361-648X/aa8f79 (2017).
10 Lejaeghere, K. *et al.* Reproducibility in density functional theory calculations of solids. *Science* **351**, aad3000 (2016).
11 Perdew, J. P., Burke, K. & Ernzerhof, M. Generalized gradient approximation made simple. *Phys. Rev. Lett.* **77**, 3865, doi:DOI 10.1103/PhysRevLett.77.3865 (1996).
12 Grimme, S. Semiempirical GGA-type density functional constructed with a long-range dispersion correction. *J. Comput. Chem.* **27**, 1787-1799, doi:10.1002/jcc.20495 (2006).
13 Monkhorst, H. J. & Pack, J. D. Special points for Brillouin-zone integrations. *Phys. Rev. B* **13**, 5188, doi:DOI 10.1103/PhysRevB.13.5188 (1976).
14 Rasmussen, C. E. & Williams, C. K. *Gaussian processes for machine learning*. Vol. 1 (MIT press Cambridge, 2006).
15 Bartók, A. P., Kondor, R. & Csányi, G. On representing chemical environments. *Phys. Rev. B: Condens. Matter Mater. Phys.* **87**, 1-16, doi:10.1103/PhysRevB.87.184115 (2013).
16 De, S., Bartók, A. P., Csányi, G. & Ceriotti, M. Comparing molecules and solids across structural and alchemical space. *Phys. Chem. Chem. Phys.* **18**, 13754-13769, doi:10.1039/C6CP00415F (2016).
17 Bartok, A. P. *et al.* Machine learning unifies the modeling of materials and molecules. *Sci. Adv.* **3**, e1701816, doi:ARTN e1701816 10.1126/sciadv.1701816 (2017).
18 Ceriotti, M., Tribello, G. A. & Parrinello, M. Demonstrating the transferability and the descriptive power of sketch-map. *J. Chem. Theory Comput.* **9**, 1521-1532, doi:10.1021/ct3010563 (2013).
19 Campello, R. J., Moulavi, D., Zimek, A. & Sander, J. Hierarchical density estimates for data clustering, visualization, and outlier detection. *ACM Trans. Knowl. Discov. Data* **10**, 5, doi:Artn 5 10.1145/2733381 (2015).
20 Salager, E. *et al.* Powder Crystallography by Combined Crystal Structure Prediction and High-Resolution H-1 Solid-State NMR Spectroscopy. *J. Am. Chem. Soc.* **132**, 2564-+, doi:10.1021/ja909449k (2010).
21 Hofstetter, A. & Emsley, L. Positional Variance in NMR Crystallography. *J. Am. Chem. Soc* **139**, 2573-2576, doi:10.1021/jacs.6b12705 (2017).
22 Carignani, E., Borsacchi, S., Bradley, J. P., Brown, S. P. & Geppi, M. Strong intermolecular ring current influence on 1H chemical shifts in two crystalline forms of naproxen: a combined solid-state NMR and DFT study. *The Journal of Physical Chemistry C* **117**, 17731-17740 (2013).
23 Uldry, A.-C. *et al.* Quantifying weak hydrogen bonding in uracil and 4-Cyano-4 '-ethynylbiphenyl: a combined computational and experimental investigation of NMR chemical shifts in the solid state. *J. Am. Chem. Soc.* **130**, 945-954 (2008).
24 Sardo, M. *et al.* Diazole-based powdered cocrystal featuring a helical hydrogen-bonded network: Structure determination from PXRD, solid-state NMR and computer modeling. *Solid State Nucl Mag* **65**, 49-63 (2015).
25 Hartman, J. D., Kudla, R. A., Day, G. M., Mueller, L. J. & Beran, G. J. O. Benchmark fragment-based H-1, C-13, N-15 and O-17 chemical shift predictions in molecular crystals. *Phys. Chem. Chem. Phys.* **18**, 21686-21709, doi:10.1039/c6cp01831a (2016).
26 Arico-Muendel, C. C. *et al.* Orally Active Fumagillin Analogues: Transformations of a Reactive Warhead in the Gastric Environment. *ACS Med. Chem. Lett.* **4**, 381-386, doi:10.1021/ml3003633 (2013).
27 Dao, H. T., Li, C., Michaudel, Q., Maxwell, B. D. & Baran, P. S. Hydromethylation of Unactivated Olefins. *J. Am. Chem. Soc.* **137**, 8046-8049, doi:10.1021/jacs.5b05144 (2015).
28 Garozzo, D. *et al.* Inclusion networks of a calix[5]arene-based exoditopic receptor and long-chain alkyldiammonium ions. *Org. Lett.* **5**, 4025-4028, doi:10.1021/ol035310b (2003).
29 Bats, J. W. *CSD Communication* (2010).
30 Huang, G. B. *et al.* Selective recognition of aromatic hydrocarbons by endo-functionalized molecular tubes via C/N-H center dot center dot center dot pi interactions. *Chin. Chem. Lett.* **29**, 91-94, doi:10.1016/j.cclet.2017.07.005 (2018).
31 Plater, M. J., Harrison, W. T. A., de los Toyos, L. M. M. & Hendry, L. The consistent hexameric paddle-wheel crystallisation motif of a family of 2,4-bis(n-alkylamino)nitrobenzenes: alkyl = pentyl, hexyl, heptyl and octyl. *J. Chem. Res.*, 235-238, doi:10.3184/174751917x14902201357356 (2017).
