# Supplementary material for: Chemical shifts in molecular solids by machine learning
Source: Nat Commun. 2018 Oct 29;9:4501. doi: 10.1038/s41467-018-06972-x (PMC6206069; doi:10.1038/s41467-018-06972-x)
Supplement: Supplementary file 2 — Description of Additional Supplementary Files [file 41467_2018_6972_MOESM2_ESM.pdf]

Description of Additional Supplementary Files

File Name: Supplementary Data 1
Description: The file Supplementary_Dataset_1.xyz contains the Quantum Espresso relaxed geometries and the corresponding DFT chemical shieldings of the structures in the CSD-2k set. For each atom in the xyz file we report: atom type, Cartesian coordinates and GIPAW calculated isotropic chemical shielding.

File Name: Supplementary Data 2
Description: The file Supplementary_Dataset_2.xyz contains the Quantum Espresso relaxed geometries and the corresponding DFT chemical shieldings of the structures in the CSD-500 set. For each atom in the xyz file we report: atom type, Cartesian coordinates, GIPAW calculated isotropic chemical shielding and ShiftML calculated isotropic chemical shielding.

File Names: Supplementary Data 3 and 5
Description: The folders Supplementary_Dataset_3 and Supplementary_Dataset_5 contain the 1H chemical shieldings calculated with GIPAW and ShiftML for AZD8329 and cocaine candidate crystal structures respectively. The geometries were relaxed using Castep, and the relaxed geometries are available from doi:10.1039/c3cp41095a and doi:10.1021/ja4088874. The chemical shift values are given as separate .cs files, named according to the corresponding structure. Each file contains the 1H chemical shift calculated with GIPAW (first column) and predicted with ShiftML (second column) ordered according to Supplementary Table 1.

File Names: Supplementary Data 4 and 6
Description: The folders Supplementary_Dataset_4 and Supplementary_Dataset_6 contain the 1H chemical shieldings calculated with GIPAW and ShiftML for AZD8329 and cocaine candidate crystal strucutres respectively. The geometries were relaxed using Quantum Espresso and the relaxed geometries are given as xyz files. The chemical shift values are given as separate .cs files, named according to the corresponding structure. Each file contains the 1H chemical shift calculated with GIPAW (first column) and predicted with ShiftML (second column) ordered according to Supplementary Table.

File Name: Supplementary Data 7
Description: The folder Supplementary_Dataset_7 contains all the data used for Figure 5 and Supplementary Figure 4.

File Name: Supplementary Data 8
Description: The folder Supplementary_Dataset_8 contains the 1H chemical shieldings calculated with GIPAW and ShiftML for the crystal structures used for the comparison of ShiftML to experimentally measured shifts. The chemical shift values are given together with the geometries in the .xyz files named according to the corresponding structure. For each atom in the xyz file we report: atom type, Cartesian coordinates, GIPAW calculated isotropic chemical shielding and ShiftML calculated isotropic chemical shielding.

File Name: Supplementary Data 9
Description: The Supplementary_Dataset_9 folder contains Quantum Espresso relaxed geometries of

the CSD-6 set. The geometries are given as separate files named accordingly to the CSD refcode. For each atom in the xyz files we report: atom type, Cartesian coordinates and isotropic chemical shielding predicted with ShiftML.
